# A Spatial Modeling Approach to Predicting the Secondary Spread of Invasive Species Due to Ballast Water Discharge

**DOI:** 10.1371/journal.pone.0114217

**Published:** 2014-12-03

**Authors:** Jennifer L. Sieracki, Jonathan M. Bossenbroek, W. Lindsay Chadderton

**Affiliations:** 1 Lake Erie Center, Department of Environmental Sciences, University of Toledo, Oregon, Ohio, United States of America; 2 Great Lakes Project, The Nature Conservancy, c/o Environmental Change Initiative, University of Notre Dame, South Bend, Indiana, United States of America; Institut Maurice Lamontagne, Canada

## Abstract

Ballast water in ships is an important contributor to the secondary spread of invasive species in the Laurentian Great Lakes. Here, we use a model previously created to determine the role ballast water management has played in the secondary spread of viral hemorrhagic septicemia virus (VHSV) to identify the future spread of one current and two potential invasive species in the Great Lakes, the Eurasian Ruffe (*Gymnocephalus cernuus*), killer shrimp (*Dikerogammarus villosus*), and golden mussel (*Limnoperna fortunei*), respectively. Model predictions for Eurasian Ruffe have been used to direct surveillance efforts within the Great Lakes and DNA evidence of ruffe presence was recently reported from one of three high risk port localities identified by our model. Predictions made for killer shrimp and golden mussel suggest that these two species have the potential to become rapidly widespread if introduced to the Great Lakes, reinforcing the need for proactive ballast water management. The model used here is flexible enough to be applied to any species capable of being spread by ballast water in marine or freshwater ecosystems.

## Introduction

Invasive species have been identified as one of the major threats to the biodiversity of freshwater ecosystems, including the Laurentian Great Lakes [1], [2]. Since the opening of the St. Lawrence Seaway in 1959, ballast water has increasingly become the dominant pathway for non-native species to enter the Great Lakes [3], [4] and an important vector of secondary spread (i.e. spread that occurs upon invading a new location) of invasive species and diseases [5]–[7]. However, despite ongoing regulatory efforts to prevent transoceanic introductions of species via ballast water, ships are not being regulated within the Great Lakes. At the same time, there is renewed interest in establishing basin wide surveillance programs to detect introductions early in the invasion process, in part generated by the potential of new genomic detection tools [8]. In order to focus detection and monitoring efforts and plan prevention, response, and containment, it is important to predict locations of potential introduction and patterns of spread within the Great Lakes. The purpose of our study was to create a dynamic spatial model that predicts the secondary spread of invasive species by ballast water. In particular, we report the results of predictions made for one established, but localized, Great Lakes invader (Eurasian Ruffe, *Gymnocephalus cernuus*), and two predicted future invaders (killer shrimp, *Dikerogammarus villosus*, and golden mussel, *Limnoperna fortunei*). These three species were prioritized by Great Lakes resource managers and scientists as species whose spread around the Great Lakes may be enhanced by movement of ballast water. The species chosen are representative of probable future invasion management challenges in the region, but our approach may be applied to any species that may be moved via ballast water and to any ecosystem that may experience invasions due to commercial shipping.

**Figure 1:**
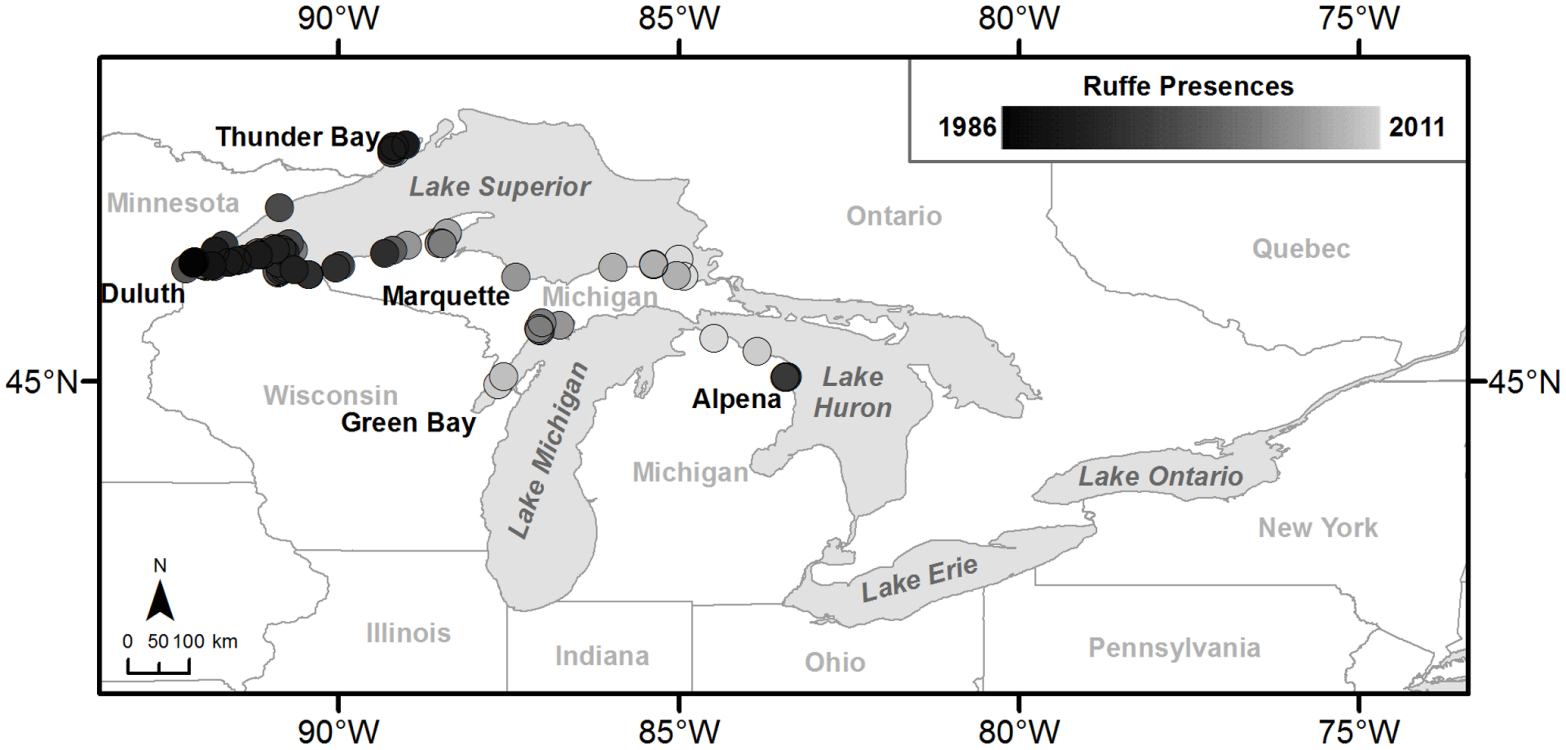
Eurasian Ruffe presences from 1986 to 2011. Ruffe data were obtained from the Nonindigenous Aquatic Species (NAS) database (USGS 2009).

**Figure 2:**
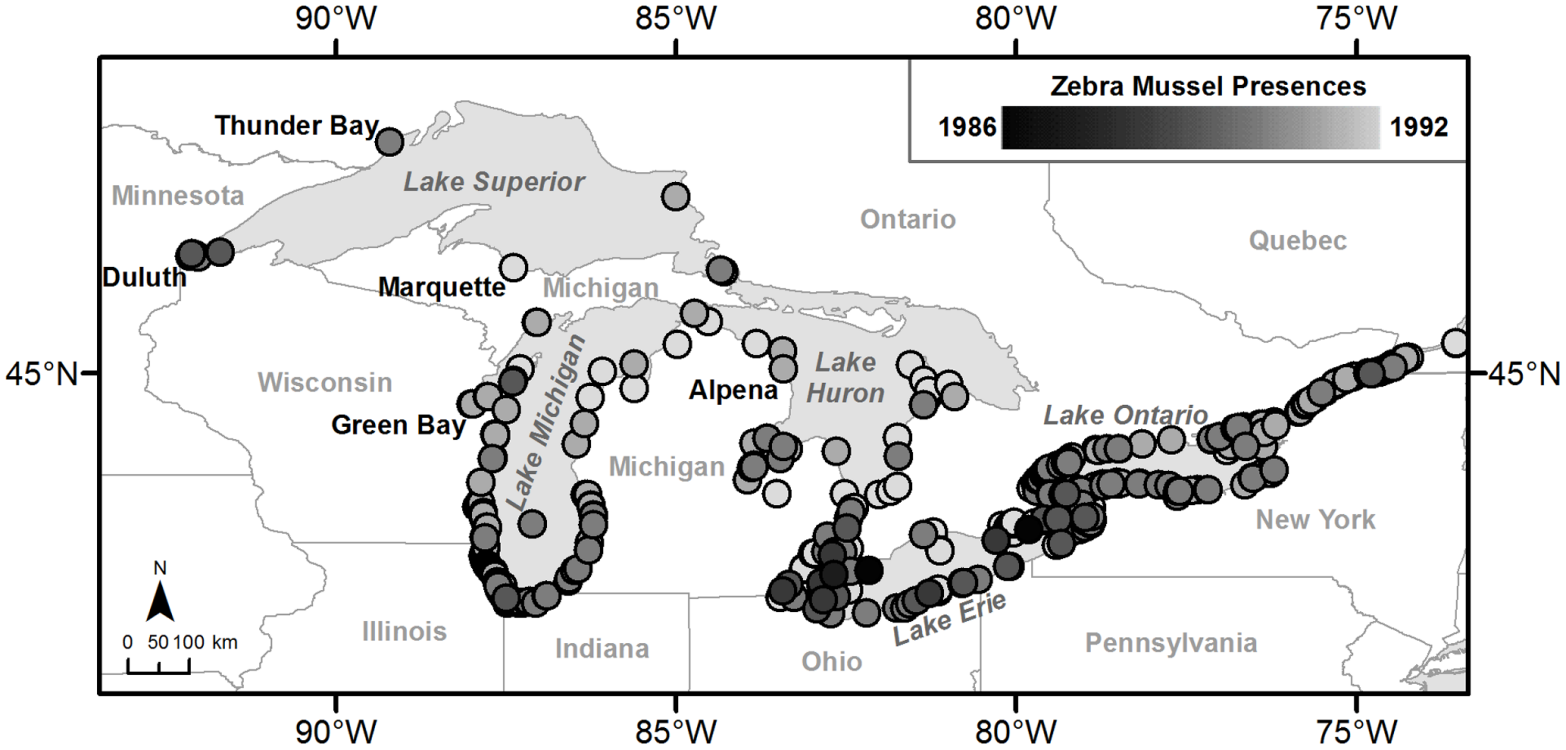
Zebra mussel presences from 1986 to 1992. Zebra mussel data were obtained from the Nonindigenous Aquatic Species (NAS) database (USGS 2009).

To date, of the species we considered, only Eurasian Ruffe have been detected in the Great Lakes. Ruffe is a species of fish from Eurasia with a Great Lakes distribution limited to Lake Superior and the northern portions of Lakes Michigan and Huron [9], [10] (Figure 1). The potential spread of ruffe is of concern because it is capable of competing with yellow perch, a native species of commercial importance [11]–[13]. On the other hand, golden mussel and killer shrimp have not been detected in the Great Lakes. Golden mussel is a species of bivalve from Southeast Asia that has invaded Hong Kong, Japan, and South America [14]–[18]. Golden mussel is very similar to zebra mussel (*Dreissena polymorpha*), which is already widespread in the Great Lakes (Figure 2). Like the zebra mussel, it has the potential to generate similar economic and ecological costs [19]. Finally, killer shrimp is a species of amphipod from the Ponto-Caspian region that has already invaded parts of Europe via the Rhine-Main-Danube canal system [20]–[23] and more recently the United Kingdom [24]. Concern about an invasion by killer shrimp stems from its indiscriminate predation habits and ability to outcompete smaller, native amphipods [20], [25], [26]. It has been reported that killer shrimp will at times kill prey as large as larval fish and do not always consume organisms upon killing them [20].

Predictive models are increasingly being used to identify how human-mediated vectors spread invasive species. For instance, Schneider et al. (1998) and Bossenbroek et al. (2007) used gravity models to identify lakes that were most at risk for future invasion of zebra mussels [27], [28]. On the other hand, Drake and Mandrak (2010) used least-cost transportation networks to identify how anglers may potentially spread invasive species throughout Ontario [29]. Predictive models that include a human-mediated vector have also been applied to terrestrial invasive species. Prasad et al. (2010) used a spatially explicit cell-based model to identify the risk of emerald ash borer (*Agrilus planipennis*) spread in Ohio due to both natural and human-mediated vectors [30]. Outside North America, Carrasco et al. (2010) discovered that both domestic and international human-mediated vectors were important in explaining the past spread of western corn rootworm (*Diabrotica virgifera* ssp. *virgifera*) in Austria [31]. Previously, we explained past patterns of spread of the fish disease viral hemorrhagic septicemia virus (VHSV) in the Great Lakes, using a dynamic spatial model that incorporated the number of ballast water discharge events a location receives and species invasion probability [7]. Our model differs from the examples listed here in that rather than identifying the pattern of spread by quantifying the “attractiveness” or likelihood of an area to become infested based on its characteristics, we used recent ballast water discharge data to establish a network of ballast water movement in the Great Lakes.

Ballast water discharge data has been used before to conduct risk assessments for ports in the Great Lakes and throughout North America. For example, Ruiz et al. (2013) used the number of ship trips and amount of ballast water discharged at U.S. ports to determine if nonnative species richness is related to shipping activity [32]. Their results found no difference in species richness between those areas with high and low shipping activity, indicating that such data would not provide for an accurate assessment of risk. Nonetheless, Ruiz et al. (2013) suggested that the inclusion of ballast water source data may have allowed for the differentiation of species richness between sites [32]. Some risk assessments have included source information covering a variety of geographic extents to not only identify the probability that a port will be invaded in the future, but to also summarize from where that risk is likely to originate [5], [33]–[35]. Unlike the risk assessments described here, we sought to create a ballast water spread model that identified the potential path of spread that a specific species could travel once it was introduced into the Great Lakes. Furthermore, unlike previous studies, our model not only includes site-specific source-discharge information, but also takes into consideration the results of species risk assessments and expert judgments, species biological requirements and behavior, known distribution of high risk invaders in source ports, and ballast water trip-specific information.

For this study, we adapt our dynamic spatial model to predict the future spread of Eurasian Ruffe, golden mussel, and killer shrimp. We used backcasting of the historic invasion pattern of zebra mussels and ruffe to identify the most important parameters and values that predicted their spread. We then predicted localities most at risk of future invasion by ruffe using the best parameter values that backcast historic ruffe spread, and those parameters that backcast historic zebra mussel dispersal were used to forecast the spread of golden mussel and killer shrimp. Based on the results of our models, we make recommendations for the future management of invasive species and ballast water in the Great Lakes.

## Methods

### Site Description

**Figure 3:**
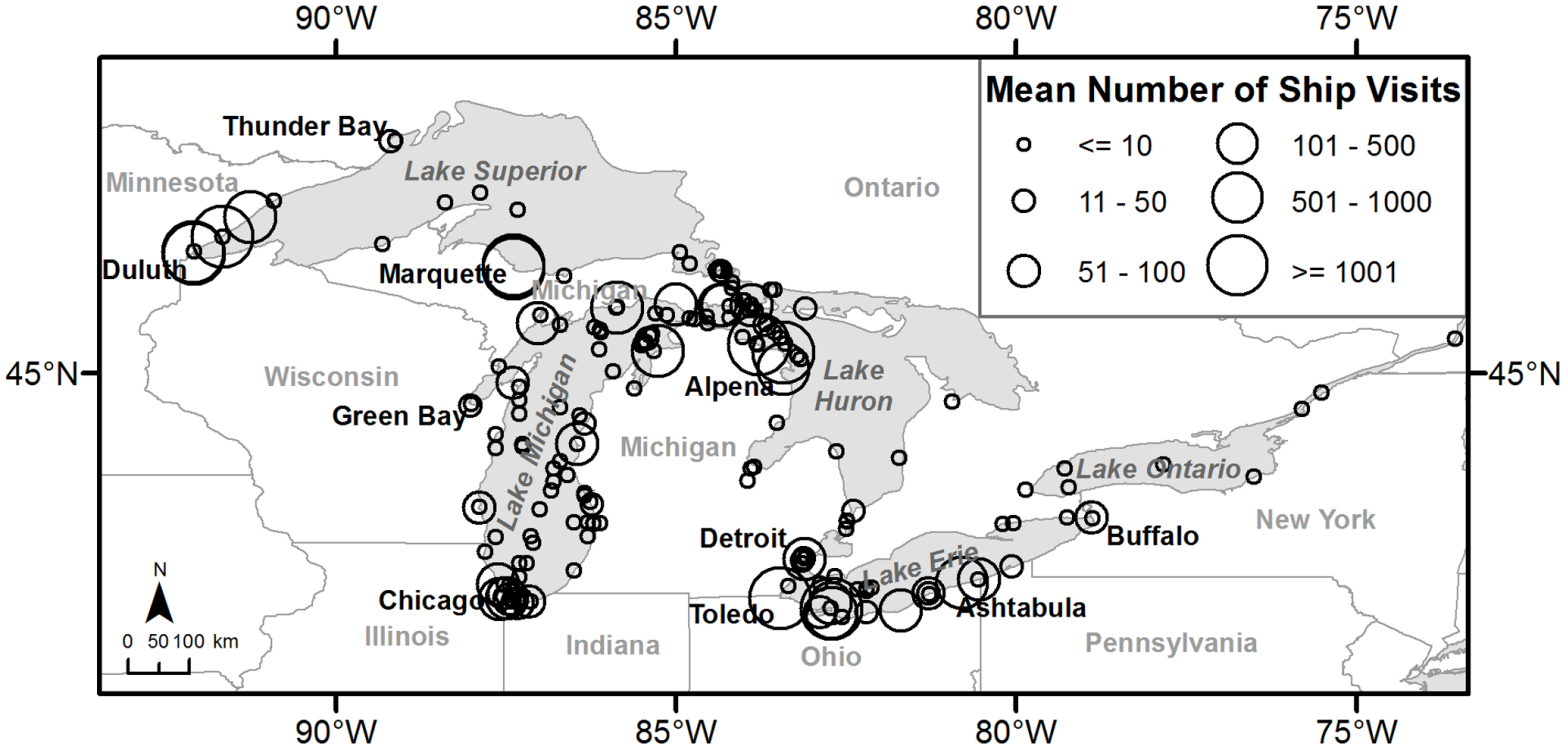
Mean number of discharging ship visits per year for each discharge location. Means between 0 and 1 were rounded up to 1. Ship visit data were obtained for ships visiting U.S. ports between 2004 and 2010 from the National Ballast Information Clearinghouse (Smithsonian Environmental Research Center and USCG 2009).

The Great Lakes and St. Lawrence Seaway were the water bodies of interest for this study. The St. Lawrence Seaway was defined as the portion of the St. Lawrence River from Lake Ontario downstream to the western tip of Anticosti Island. The study area included Lake St. Clair and Niagara, Detroit, St. Clair, and St. Marys Rivers, as well. Despite water in the St. Lawrence Seaway flowing eastward towards the Atlantic Ocean, the trend of ballast water movement is westward, with Duluth-Superior Harbors, Minnesota-Wisconsin, USA receiving the most ballast water each year (Figure 3). As identified by data in the National Ballast Information Clearinghouse for the years 2004 to 2010, the top 5 U.S. ballast water discharge sources are: Nanticoke, Ontario, Canada (Lake Erie), Indiana Harbor, Indiana, USA (Lake Michigan), Gary, Indiana, USA (Lake Michigan), St. Clair, Michigan, USA (St. Clair River), and Detroit, Michigan, USA (Detroit River), top 5 U.S. discharge locations are: Superior, Wisconsin, USA (Lake Superior), Two Harbors, Minnesota, USA (Lake Superior), Duluth, Minnesota, USA (Lake Superior), Calcite, Michigan, USA (Lake Huron), and Marquette, Michigan, USA (Lake Superior) [36].

### Backcasting

We parameterized our models by backcasting the spread of two invasive species that already occur in the Great Lakes, zebra mussel and Eurasian Ruffe. Zebra mussel was backcast as a surrogate for golden mussel and killer shrimp, because golden mussel have life history traits and use habitats similar to zebra mussel [19], [37], and killer shrimp have similar physical and chemical tolerances [38]. The three models based on Sieracki et al. (2013), a “random”, “location”, and “propagule pressure”, were developed for each of the two backcast species [7]. The models have the same basic structure: (1) new infestation locations are selected for each year simulated, (2) an area of infestation is identified around each new location, and (3) the invasion front is further expanded given a possible rate of local spread that may occur each year. However, the three models differ in how new infestations (Step 1) are selected.

In order to determine if ballast water was contributing to species spread we compared the location model with a random model. The random model acts as the null model, and the location model needed to perform better than the random model in order to be able attribute spread to ballast water movement. The random model does not take into consideration other invasion pathways (e.g. recreational boating, sale of live organisms, etc.) that also contribute to spread in the Great Lakes. Both the random and location models selected the number of new annual infestations by randomly selecting from a Poisson distribution. The means and variances (λ) for the distributions were set equal to the mean number of new invasions potentially due to ballast water. For zebra mussel, λ was calculated as the mean number of occurrences per year for 1986 to 1992 as identified from records in the Nonindigenous Aquatic Species (NAS) database, thus λ = 4 [39]. Unlike zebra mussel, most Eurasian Ruffe occurrences identified in the NAS database appear to be due to natural spread by the fish themselves, particularly the spread that occurred along the south shore of Lake Superior. However, four independent invasion events that were potentially due to human-mediated spread were identified from the occurrence data. These independent invasions were determined to be “human-mediated”, since they were long-distance (>50-km from the nearest infestation) and occurred in locations where large amounts of ballast water had been discharged in the past [36], [39]. Therefore mean number of invasions per year for Eurasian Ruffe was calculated as λ = 0.2. Whereas the number of infestations per year were selected using the same method for both models, each model selected the location of each new infestation differently. The “random” model identified the location of each of the newly selected infestations randomly within the Great Lakes. The “location” model randomly selected infestation locations only from known ballast water discharge locations. The results of the models allowed us to determine whether or not species infestations were correlated with ballast water locations.

Upon determining if past infestations were correlated with ballast water discharge locations, the third model, the “propagule pressure” model, was used to determine if infestation locations could better be identified if ship trip information was included. First, ballast water source locations that occurred within an infested area were identified. Next, locations that received ballast water from those infested locations were selected. To determine if the selected discharge locations actually became infested upon receiving ballast water from infested sources, the potential invasion result was selected from a binomial distribution. A result of 0 meant a trip did not end in infestation, and a result of 1 meant a trip did lead to infestation. The number of trials, n, was equal to the number of trips made to a discharge location that year by ships carrying infested ballast water. The probability of infestation for each day of the trip was varied for each species to identify the best value for the parameter. Probabilities of 0.000001, 0.0001, and 0.01 were tested for Eurasian Ruffe, and 0.05, 0.25, 0.50, and 0.75 for zebra mussels (Table 1). A single probability of invasion was used as opposed to multiple probabilities representing the rates of uptake, trip survival, and establishment in order to create a simple model that can be applied to multiple species despite the level of information available on biological and physical tolerances and habitat preferences. Probabilities for the two species differed in magnitude due to their differences in expected larval survival rates and the length of time that individuals are expected to become entrained in ballast water during a given year. Additional probabilities of infestation were tested; however, as these did not improve model accuracies, they were not included in this study. The length of the trip was determined by calculating the median of the trip lengths recorded between the source and discharge location. If at least one of the trips resulted in a binomial value of 1, then the discharge location was then considered infested.

**Table 1:** Model runs conducted in backcasting the spread of Eurasian Ruffe and zebra mussels.

			Species	
Models	Spread Distance	Probability of Infestation	Eurasian Ruffe	Zebra Mussel
**Random**	5-km	NA		X
	10-km		X	X
	20-km			X
	25-km		X	
**Location**	5-km	NA		X
	10-km		X	X
	20-km			X
	25-km		X	
**Propagule Pressure**	5-km	0.05		X
		0.25		X
		0.50		X
		0.75		X
	10-km	0.000001	X	
		0.0001	X	
		0.01	X	
		0.05		X
		0.25		X
		0.50		X
		0.75		X
	20-km	0.5		X
		0.25		X
		0.50		X
		0.75		X
	25-km	0.000001	X	
		0.0001	X	
		0.01	X	
	**Total # of Models:**		**10**	**18**

Once infestation locations were selected for a year, the dispersal of the species from the initial invasion point was then identified for all models. First, an infestation area was identified from the new invasive species occurrence. Coordinates for ballast water discharge and source locations in the National Ballast Information Clearinghouse (NBIC) were recorded with a precision no less than one one-hundredths of a degree. We calculated that in the Great Lakes, the difference between two points that were one one-hundredths of a degree apart was approximately 1.4-km. This was identified as the estimated difference that could occur between the actual and recorded discharge locations due to rounding error, and was used as the radius of the area of infestation, since the species could have potentially been discharged anywhere within that circle. To identify the rate of natural spread that could occur upon being introduced to a new location, a second radius was used to expand the area of infestation. For ruffe, the natural spread distance was identified from the rate of secondary spread along the south shore of Lake Superior that was most likely due to fish dispersal. As identified from the occurrences recorded in the NAS database, the dispersal distance was most commonly ∼25-km along the south shore of Lake Superior [39]. In addition to the 25-km distance, a 10-km spread distance was tested to determine if shorter dispersals were more common (Table 1). On the other hand, zebra mussels only have limited swimming capabilities in the larval stages; however, veligers are capable of being carried great distances in water currents [40]. Natural spread distances of 5-, 10- and 20-km were tested for the invasive bivalve (Table 1). The resulting areas of infestation were limited by lake depths identified as being inhabitable by ruffe (≤90-m) or zebra mussel (≤35-m) based on the maximum depth of occurrence locations obtained from the NAS Database for each of these species [39].

Invasive species occurrences were required to run all three models, and ballast water data were needed for the “location” and “propagule pressure” models. Zebra mussel and Eurasian Ruffe presence locations for 1986 to 1992 and 1986 to 2011 respectively were obtained from the NAS Database [39]. The NAS Database is mostly compiled from U.S. occurrence records; however, does include some data for Canada, as well. For the years prior to species detection, the species was considered to be absent from that location. Ballast water source, discharge, and trip data for the years 2004 to 2010 were obtained from the NBIC [36]. Commercial ships that visit U.S. ports are required to report ballasting operations to the NBIC. Discharges at some Canadian ports are included, as the last discharge location prior to arriving at a U.S. port was not necessarily conducted in the U.S. The mean number of visits to discharge locations from each source location for 2004 to 2010 (Figure 3) and median number of trip days were calculated from the NBIC data. The limited amounts of Canadian data identified in the ANS Database and NBIC were included, since Canadian locations potentially served as ballast water sources for U.S. discharge locations, and some Canadian species occurrences were captured by the natural spread distance.

The models were developed in Python to be run in ArcGIS (see Information S1). Scripting the models as opposed to creating them in ArcGIS ModelBuilder, as was done for the VHSV study [7], allowed for flexibility in the number of years the model could simulate and allowed for more specific trip information to be included for each source-discharge combination. The zebra mussel models were run to simulate secondary spread for 1986 to 1992, since they were widespread in the Great Lakes by 1992. The Eurasian Ruffe models simulated secondary spread for 1986 to 2011, because their rate of spread has been slow and their distribution in the Great Lakes is currently limited. Each of the models was run 100 iterations.

The model results were analyzed by calculating the overall, presence, and absence accuracies for each iteration of the model [41], [42]. The means of each of the accuracies were calculated for each of the 28 models. The best fit model was selected as having the highest overall accuracy. Where overall accuracies were similar between models, the model with the highest presence accuracy was selected, unless absence accuracies were particularly low. Then, the model with the higher absence accuracy was used as an alternative model to capture a better range of predictions.

Additionally, the length of time that would be required to spread the full extent of the current area invaded by each species if only natural spread is considered was identified. This was done by applying the largest spread distances tested above, 20-km for zebra mussel and 25-km for ruffe, to the initial introduction locations detected in 1986 for each species. The invasion front was identified for each year and was limited to the areas identified as being inhabitable by the species of interest.

### Forecasting

Upon identifying the best fit model, the next step was to predict the future secondary spread of invasive species that either already occur in the Great Lakes or may occur in the Great Lakes in the future. The three species that predictions were simulated for were the Eurasian Ruffe, golden mussel, and killer shrimp.

Prediction models differed from the backcasting models in that the current Great Lakes distribution or possible initial introduction locations were used as the initial sources of infestation for each species. Also, instead of comparing the final distributions of the model predictions to the actual occurrences of the invasive species, the total number of model iterations a port was predicted to be invaded in the future was calculated. For each model iteration, once a port was identified as invaded in a given year, it continued to be invaded for all subsequent years. Each model simulated 10 time-steps of future invasion, and each simulation was run for 100 iterations. Time-steps were used in lieu of years, as the lag between a species introduction, establishment and potential for spread is uncertain. That uncertainty is also compounded by ballast water best management practices, recently required by the U.S. Coast Guard (USCG; 2012) and U.S. Environmental Protection Agency's (USEPA) General Vessel Permit (2013), that are thought to reduce the likelihood of uptake and secondary spread within the basin [43], [44], [45]. The probability of that location becoming infested was calculated based on the 100 iterations.

The initial introduction locations, natural spread distances, and probability of infestation were different for each species. Unlike the other two species being modeled, Eurasian Ruffe already occurs in the Great Lakes. The actual occurrences of this species were used as the initial starting locations for future secondary spread. The best fit values for natural spread distance and probability of infestation were identified from the results of the backcasting exercise described above. For golden mussel and killer shrimp, the potential initial invasion locations were identified as those Great Lakes ports that received ballast water from international ports within the species' known current distribution. International ballast water source-discharge patterns were identified from the NBIC for 2004 to 2010 (Table 2) [36]. Predictions for both species were made using the parameters identified from the zebra mussel backcasting results; however, because we were uncertain as to how far killer shrimp would travel in the water column, no natural spread distance was used in forecasting this species. Further, by not including a natural spread distance, we were able to identify the secondary spread that was entirely due to the linkages between ballast water source and discharge locations, and not spread upon being discharged. Also, in the absence of a clear lower depth limit, no depth restrictions were placed on the killer shrimp models.

**Table 2:** Ports identified as having received ballast water from killer shrimp and golden mussel infested locations.

	# Ship Visits
**Killer Shrimp**	
Duluth, Minnesota, USA	147
Toledo, Ohio, USA	47
Superior, Wisconsin, USA	17
Ogdensburg, New York, USA	8
Green Bay, Wisconsin, USA	7
Goderich, Ontario, Canada	4
Detroit, Michigan, USA	1
**Golden Mussel**	
Bay City, Michigan, USA	9
Duluth, Minnesota, USA	3

The number of visits made by ships with potentially infested ballast water at each Great Lakes port was calculated from the NBIC data for 2004 to 2010 (Smithsonian Environmental Research Center and USCG, 2009).

## Results

### Backcasting

**Figure 4:**
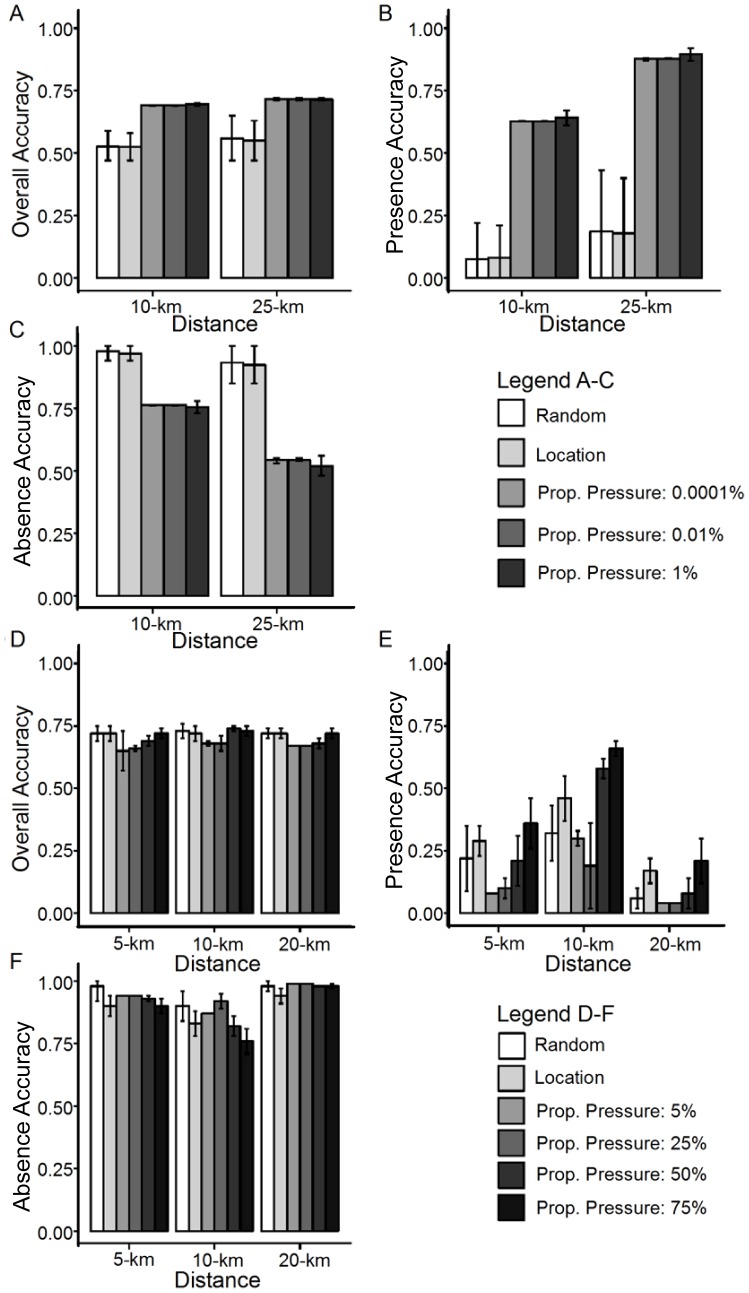
Backcasting results for Eurasian Ruffe and zebra mussel. Graphs A–C illustrate the results for Eurasian Ruffe, and graphs D–E illustrate the results for zebra mussel. Graphs A and D depict the overall accuracy of the models tested. Graphs B and E depict the presence accuracy, or ability to correctly identify presences correctly. Graphs C and F display the absence accuracy, or ability to correctly identify absences correctly. Error bars represent standard deviations.

Results of the Eurasian Ruffe backcasting identified the propagule pressure models as performing best overall, with mean overall accuracies between 0.69 and 0.72 (Figure 4A). Despite identifying absences at greater rates than the propagule pressure models, the random and location models identified very few ruffe presences, suggesting that these models would not be able to adequately predict the future spread of invasive species. (Figure 4B–C). Among the propagule pressure models, the 25-km models produced the highest presence accuracies (Figure 4B); however, also had the lowest absence accuracies (Figure 4C), suggesting that the model was over-predicting the spread of ruffe. On the other hand, the 10-km propagule pressure models produced presence accuracies that were somewhat lower than those for the 25-km model (Figure 4B), but still much higher than the location and random models. The 10-km propagule pressure models also produced higher absence accuracies than the 25-km models (Figure 4C), suggesting that these models are somewhat more conservative. Overall, the 25-km 0.0001 probability propagule pressure model performed best, but only at a rate of 0.02 over the next best performing model, the 10-km 0.01 probability propagule pressure model, so both models were identified as best fit. Further, if Eurasian Ruffe had only spread naturally at a rate of 25-km per year, it would have taken 55 years to reach the furthest extent of current invasion rather than the observed 26 years (Figure 1), signifying that the chosen models provided the most likely scenario for the secondary spread of Eurasian Ruffe.

The propagule pressure models were also the best performing in backcasting the spread of zebra mussel. Overall, the random and location models performed as well or nearly as well as the best performing propagule pressure models (Figure 4D); however, the addition of ballast water information increased the presence accuracy for each natural spread distance tested (Figure 4E). Furthermore, the probability of infestation proved to be an important parameter in backcasting zebra mussel. At the lower values tested, it reduced the ability of the model to predict presences, whereas at the highest value of 0.75 the presence accuracy was increased at all spread distances tested (Figure 4E). Despite an increase in presence accuracy generally leading to a decrease in absence accuracy, the lowest absence accuracy was still greater than 0.75, indicating that while some models may have been under-predicting occurrences, they were not over-predicting them (Figure 4F). Additionally, it would take 83 years (opposed to four) for zebra mussels to naturally disperse at a rate of 20-km per year (assuming they could spread upstream unaided – which seems unlikely) to reach the western most edge of their known 1992 extent (Figure 2). This suggests that zebra mussels spread much more rapidly than would be expected due to natural dispersal, and that the best fit model explained zebra mussel spread better when ballast water information was included.

### Forecasting

**Figure 5:**
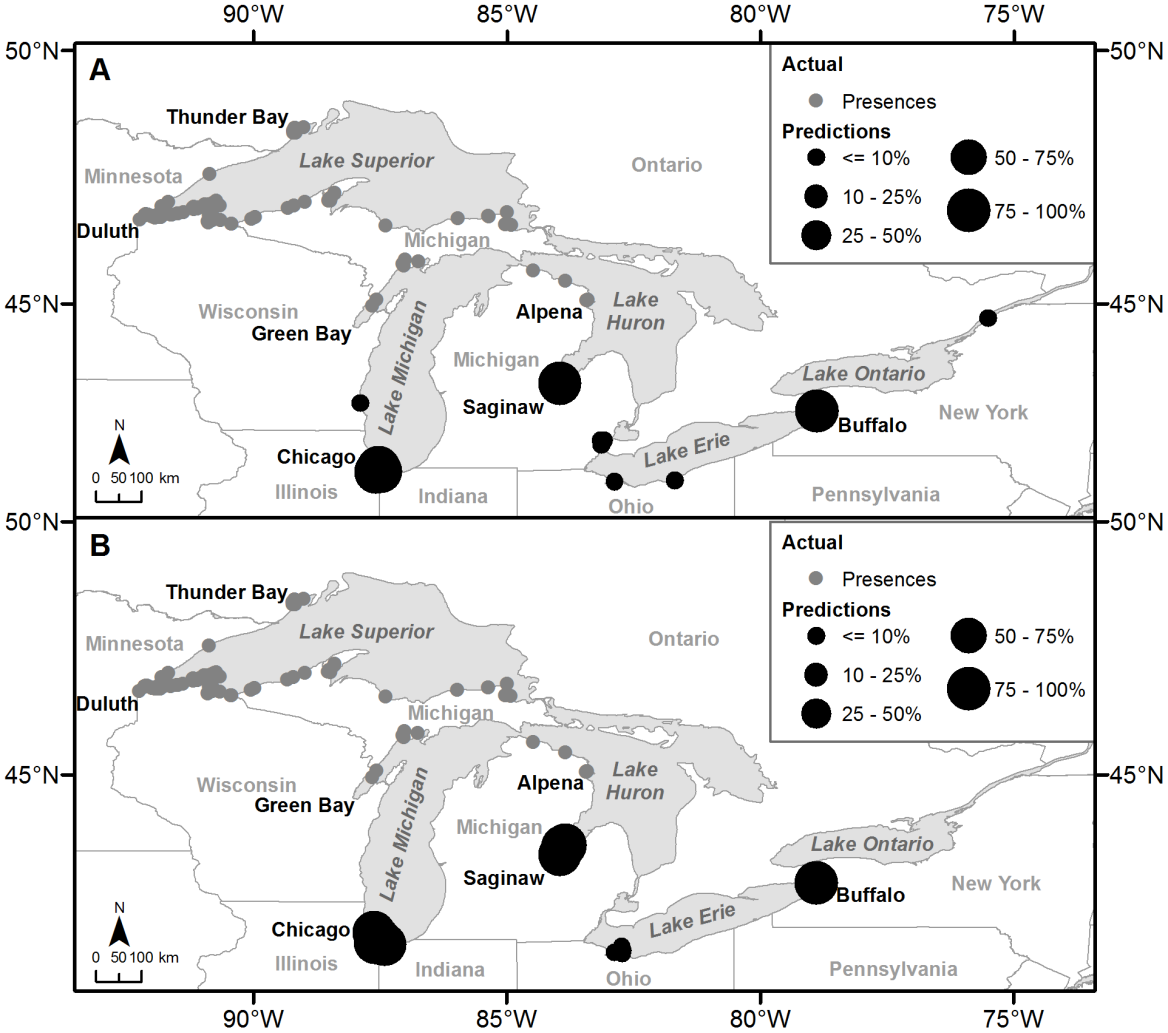
Eurasian Ruffe prediction results. The maps illustrate the results of the Eurasian Ruffe prediction models with Figure 5A dispersal distance  = 10-km and probability of infestation  = 0.01 and Figure 5B dispersal distance  = 25-km and probability of infestation  = 0.0001. The maps depict the next likely invaded locations from estimated presences.

In order to capture a range of possible outcomes for the future spread of Eurasian Ruffe, both models identified by Eurasian Ruffe backcasting above (10-km 0.01 probability and 25-km 0.001 probability) were used to forecast future secondary spread. The predictions made based on the two models depict relatively similar patterns of spread (Table 3; Figure 5A–B). Both models predict that Buffalo, New York, USA the Chicago, Illinois, USA area, and the Saginaw Bay of Lake Huron are the next most likely locations to be invaded by Eurasian Ruffe (Table 3; Figure 5A–B). The ports predicted within the Chicago area varied for each model, but potentially include the Ports of Calumet, Illinois, USA, Whiting, Indiana, USA, and Chicago, Illinois, USA among others (Table 3). The Sandusky, Ohio, USA area is also predicted by both models to have a small chance of becoming invaded. Milwaukee, Wisconsin, USA, the Detroit, Michigan, USA area, Cleveland, Ohio, USA, and Prescott, Ontario, Canada were predicted to become invaded by Eurasian Ruffe in less than 10% of the model simulations.

**Table 3:** Prediction results for the top 25 U.S. ports receiving the most visits by de-ballasting ships.

				Ruffe: 10-km 1%	Ruffe: 25-km 0.01%	Golden Mussel: 10-km 75%		Killer Shrimp: 75%					
Rank	Port	State	Waterbody			Bay City	Duluth	Duluth	Toledo	Ogdensburg	Green Bay	Goderich	Detroit
1	Superior	WI	Superior	—	—	100	100	100	99	0	99	71	99
2	Two Harbors	MN	Superior	—	—	99	100	100	99	0	99	0	99
3	Calcite	MI	Huron	—	—	100	25	0	99	0	99	96	100
4	Marquette	MI	Superior	—	—	100	99	98	99	0	99	0	100
5	Duluth	MN	Superior	—	—	100	—	—	99	23	64	55	100
6	Presque Isle	MI	Superior	—	—	100	99	0	99	0	99	0	99
7	Toledo	OH	Erie	0	0	94	15	0	—	0	0	72	99
8	Stoneport	MI	Huron	95	0	100	11	0	99	0	99	64	99
9	Marblehead	OH	Erie	0	1	75	15	0	99	50	0	0	100
10	Silver Bay	MN	Superior	95	97	NA	NA	100	99	0	99	0	99
11	Sandusky	OH	Erie	0	1	75	15	0	96	53	0	61	99
12	Ashtabula	OH	Erie	0	0	0	84	85	99	98	0	0	100
13	Port Inland	MI	Michigan	95	97	100	0	0	97	0	99	0	98
14	Alpena	MI	Huron	—	—	99	100	100	99	0	99	79	100
15	Charlevoix	MI	Michigan	0	0	0	0	0	0	0	99	0	98
16	Port Dolomite	MI	Huron	95	100	100	12	0	99	0	99	0	100
17	Drummond Island	MI	Huron	16	2	NA	NA	0	99	0	99	62	100
18	Conneaut	OH	Erie	0	0	100	100	60	99	0	41	0	100
19	Escanaba	MI	Michigan	—	—	91	0	0	21	0	99	0	99
20	Chicago	IL	Michigan	0	97	68	49	0	99	0	0	0	29
21	Cleveland	OH	Erie	3	0	93	0	0	99	80	0	0	100
22	Calumet	IL	Michigan	95	97	100	100	0	27	0	0	0	1
23	Cedarville	MI	Huron	95	100	100	12	0	99	0	99	0	98
24	Whiting	IN	Michigan	95	97	100	100	0	93	0	85	0	19
25	Detroit	MI	Detroit River	4	0	100	100	0	99	0	0	0	—

Numbers represent the number of iterations out of 100 that were predicted to become invaded in the first year modeled. Ports that were outside of the area considered habitable for a species are indicated by NA.

In order to forecast the secondary spread of golden mussel and killer shrimp, the best performing model and parameters identified by backcasting zebra mussel were used. Since none of the models were found to over-predict zebra mussel occurrences, the model with the highest presence accuracy, the 20-km propagule pressure model with a probability of infestation of 0.75, was chosen. This model also had one of the highest overall accuracies.

**Figure 6:**
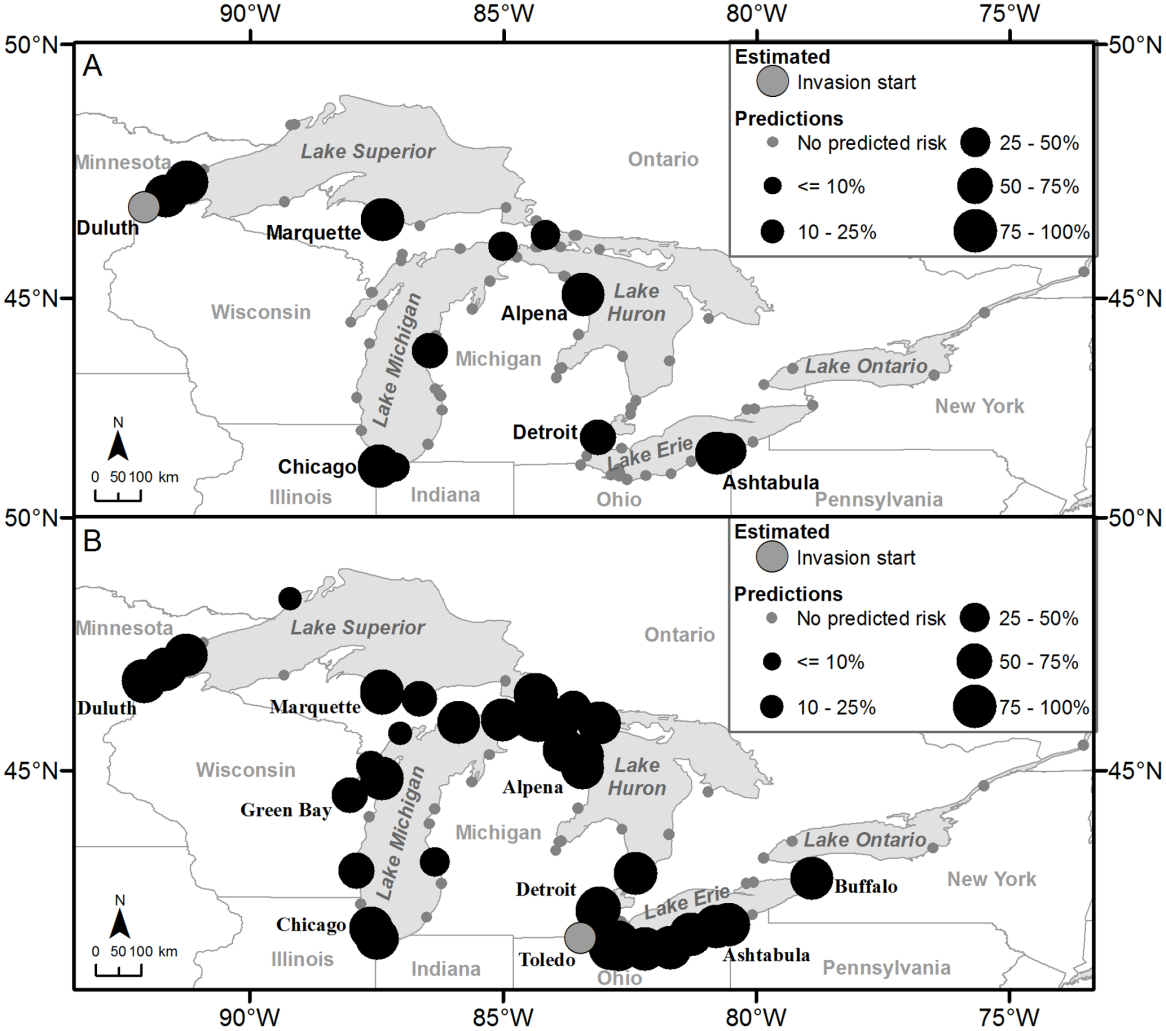
Killer shrimp prediction results. The maps illustrate the results of the killer shrimp prediction models with probability of infestation  = 0.50 and no dispersal distance. Invasions were started from Figure 6A Duluth, Minnesota, USA and Figure 6B Toledo, Ohio, USA. The maps depict the next likely invaded locations from current observed presences.

Our analysis of the NBIC 2004–2010 data indicated seven ports historically received shipping from the global range of killer shrimp. Forecasting results predict that killer shrimp could become widespread within three to four time-steps of invasion. If the species invades Duluth, Minnesota, USA first, it is predicted to most likely spread to Two Harbors (100 out of 100 model iterations) and Silver Bay (100), Minnesota, USA, Marquette (98) and Alpena (100), Michigan, USA, Indiana Harbor (93), Indiana, USA, and Ashtabula (85), Ohio, USA next (Table 3; Figure 6A). By the second and third time-steps after invasion, it is predicted to have a high probability of being widespread in Lakes Michigan, Huron, and Erie, and is predicted to invade Prescott, Ontario, Canada 74 out of 100 model iterations. By the fourth time-step killer shrimp is predicted to be widespread throughout the Great Lakes. If the initial invasion location for killer shrimp is Toledo, Ohio, USA, by the first time-step it is predicted to invade Duluth (99 out of 100 times), Two Harbors (99) and Silver Bay (99), Minnesota, USA, much of the Upper Peninsula of Michigan (21–99), USA, Alpena (99) and the Detroit area (99) in Michigan, USA, Sturgeon Bay (87), Wisconsin, USA, the Chicago area (27–99) in Illinois and Indiana, USA, and Sarnia (96), Ontario, Canada (Table 3; Figure 6B). By the second time-step, killer shrimp is predicted to be widespread in Lakes Superior, Michigan, Huron, and Erie, and is predicted to invade Hamilton, Ontario, Canada (53) in Lake Ontario and Prescott, Ontario, Canada (73) in the St. Lawrence River. By the third time-step, killer shrimp is predicted to be widespread in the Great Lakes. Maps with the results of all predictions for the remaining invasion locations and years are included in Information S2.

**Figure 7:**
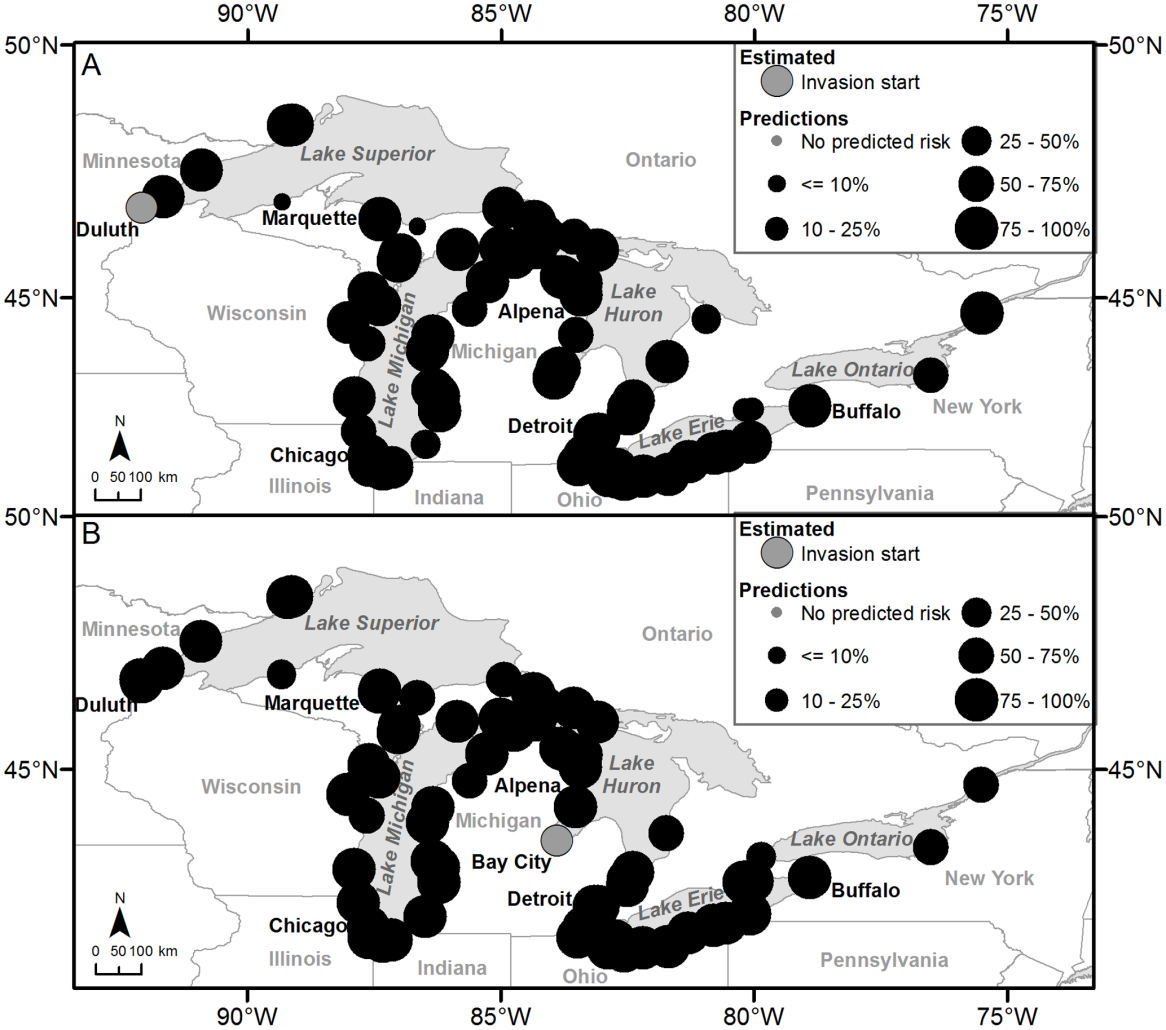
Golden mussel prediction results. The maps illustrate the results of the golden mussel prediction models with dispersal distance  = 20-km and probability of infestation  = 0.50. Invasions were started from Figure 7A Duluth, Minnesota, USA and Figure7B Bay City, Michigan, USA. The maps depict the next likely invaded locations from estimated presences.

Results of golden mussel forecasting indicate that regardless of whether Duluth, Minnesota, USA or Bay City, Michigan, USA (the two U.S. ports receiving ships from invaded international ports) are invaded first, this invasive species will spread rapidly throughout the Great Lakes, much as zebra mussel did (Figure 2). By the first time-step, golden mussel is predicted to be found in all of the Great Lakes except Lake Ontario (Figure 7A). If golden mussel invades Duluth first, it is predicted to spread to Marquette (99 out of 100 model iterations), Ludington (99), Alpena (100), Saint Clair (93), and Detroit (100), Michigan, USA, the Chicago area (49–100) in Illinois and Indiana, USA, and Conneaut (100) and Ashtabula (84), Ohio, USA (Table 3; Figure 7A). By the second time-step, golden mussel could potentially be widespread throughout the Great Lakes with predictions for invading Prescott, Ontario, Canada (78), and Oswego, New York, USA (54). If golden mussel invades Bay City first, the species will become more widespread by the first time-step than if it were to invade Duluth first (Figure 7B). Locations that were predicted to be invaded by the first time-step include Duluth (100) and Two Harbors (99), Minnesota, USA, Superior (100), Wisconsin, USA, Marquette (100), Ludington (100), Detroit (100), Michigan, USA, the northern portions of Lakes Michigan (91–100) and Huron (99–100) in Michigan, USA, the Chicago area (68–100) in Illinois and Indiana, USA, and Toledo (94), Cleveland (93), Conneaut (100), and Sandusky (75), Ohio, USA (Table 3; Figure 7B). By the second time-step, Oswego, New York, USA and Prescott, Ontario, Canada both are predicted to be invaded 57 and 70 model iterations out of 100, respectively.

## Discussion

Our ballast water model simulates the potential spread of invasive species once they become established in the Great Lakes; whereas, previous assessments have focused on identifying the first ports of introduction to the basin. By applying source- and species-specific data to generate spread predictions, we were able to attribute ballast water as a vector of spread. Ruiz et al. (2013) previously found that there was no relationship between nonnative species richness and ballast water volume and number of ship arrivals at U.S. ports when data on ballast source locations were not considered [32]. However, the risk of invasive species introductions from ballast water discharge varies, with the greatest risk posed from environmentally similar sources that also support harmful organisms [32], [35]. Additionally, transit time likely affects whether a species will be released alive [32]. Although researchers have used broad source categories to assess the risk of invasion for ports in North America, few have analyzed the potential invasion risk from specific regions of the world [5], [33], [46]. Those researchers that have identified risk from more specific source locations have not attempted to simulate the potential spread of specific species between source and discharge locations [34], [35]. For these reasons, our modelling efforts are unique in that they not only include source- and species-specific information as a means to reduce the limitations of ballast water data as an effective predictor of invasion, but that they also may be used to establish the pattern of spread as opposed to identifying a location's risk to becoming invaded by any of a number of species in the future.

The inclusion of source information in predicting the spread of invasive species was important in identifying ports that may become invaded in the future. For instance, despite not being amongst the top 25 ports receiving the most visits by discharging ships (Table 3), both Saginaw Bay and Buffalo, New York, USA were predicted to become invaded next by Eurasian Ruffe, even though their ballast water discharge history differ. Buffalo receives a sizeable amount of ballast water with an average of 73 ship visits a year (Figure 3), whereas Saginaw Bay receives very few ship visits. Nonetheless, the ballast water discharged in Saginaw Bay is frequently sourced from areas that are closer and identified as infested with Eurasian Ruffe, increasing the likelihood that each ballast discharge will contain live ruffe propagules. Another unusual prediction that our model made was the potential for Prescott, Ontario, Canada on the St. Lawrence River, to become invaded by Eurasian Ruffe three out of 100 model iterations. Even though Prescott is a small port that receives few ship visits, during the course of our ballast water discharge time series it did receive a single ship visit from Alpena, which was enough for the model to predict the location to become invaded three times. Further predictions of invasion of killer shrimp and golden mussel for Prescott (73 and 78 iterations, respectively) were also driven by the earlier invasion of Alpena. The invasion of Prescott highlights the importance of including source information in our ballast water spread model, because if we had not, we may have overlooked a number of places within the Great Lakes with the potential of being invaded in the future.

The ability to predict the future spread of invasive species is an important part of any biosecurity surveillance and response program. Although prevention of new species invasions is expected to be the least expensive option for managing invasive species, early detection, containment, and eradication is the next best option when prevention has failed [47], [48]. Delimiting the full extent of a recently discovered introduction is critical to the success of any incursion response [49], but can be particularly problematic in aquatic environments where detection of rare organisms can be challenging [8]. Here we demonstrate how a ballast water spread model can be used to predict locations where a newly introduced invader is most likely to be spread, enabling what are usually limited surveillance resources to be focused onto a subset of high priority locations. Such information increases the probability that outlying populations can be identified, contained, and potentially eradicated [50].

The importance of prediction as part of a surveillance and response program is best illustrated by our predicted spread of Eurasian Ruffe across the remaining parts of the Great Lakes basin, namely southern Lakes Michigan and Huron, and Lakes Erie and Ontario. Our predictions identified three locations at high risk for invasion, and six additional sites with lower invasion risk based on current ballast water movement patterns (Figure 5A and B). These outputs can and have already been used to inform ruffe surveillance efforts across the Great Lakes Basin, and monitoring efforts motivated by our research has resulted in the detection of Eurasian Ruffe environmental DNA (eDNA) in Calumet Harbor in Chicago, Illinois, USA (Andrew Tucker, The Nature Conservancy, pers. comm.), which was predicted 95–97% of the time to be invaded next. Based on the remaining predictions modeled, shipping may potentially speed the spread of this invasive fish into regions of the Great Lakes that would otherwise not be affected for many years.

Unlike Eurasian Ruffe, killer shrimp and golden mussels have not been detected in the Great Lakes; however, if they are introduced, they are predicted to spread rapidly. Golden mussel has life history traits similar to zebra mussel [19], and we would expect spread to match that of zebra mussels, indicating that this species could become widespread within two years of introduction. Given that killer shrimp produce fewer young per individual compared to zebra mussels, the amount of time each time-step represents is uncertain. However, this species tends to be female-biased and reproduce early and frequently throughout the year [51], suggesting that it could potentially spread as quickly as zebra mussels did. Nonetheless, there is greater uncertainty in the timeframe of killer shrimp spread predictions due to a lack of knowledge as to how differences in its reproductive and diel behavior affect length of model time steps and its ability to become entrained in ballast water. Further limitations on our predictions for killer shrimp and golden mussel include increased uncertainty in the zebra mussel occurrence data, as opposed to the Eurasian Ruffe data, and rapid speed with which zebra mussels spread. Because detection of zebra mussels in the Great Lakes was at least two years behind actual invasion and occurred so rapidly, the actual pattern of spread is difficult to ascertain. In fact, the species was recorded in all Great Lakes within two years of its first detection, suggesting the data that our model is based upon may not be a fully accurate picture of how the actual spread occurred [39], [52]. However, model results were still able to capture a large proportion of past spread for zebra mussel, suggesting that it is capable of predicting future spread with enough accuracy to inform management decisions. Our results for killer shrimp and golden mussel further emphasize the need for protective binational (i.e. the United State and Canada) ballast water treatment measures that minimize the potential for introduction of these and other species into the Great Lakes.

Shipping is the most important pathway of introduction and spread of invasive species in marine, freshwater, and estuarine environments [4], [35], [37], [53]–[55]. Globally there is increasing emphasis being placed on establishment of national port surveillance programs to detect incipient invasions from this pathway [56], but these approaches need to be coupled with dynamic spread models because of the limitations of detecting species in aquatic environments [8], [57]. Additionally, limited resources typically constrain surveillance sampling efforts and periodicity, increasing the likelihood that secondary spread will have occurred by the time an incipient invasion is detected. The dynamic spatial model described here could easily be modified for new geographies. It has been built to run in ArcGIS, a commonly used program by government agencies and universities, is relatively easy to run, and requires few inputs, including the natural spread distance and probability of invasion. Further, other data can be readily added to the model in the future, such as habitat information. The model can also be retrofitted to run predictions for any aquatic system receiving ballast water discharges, so long as ballast water data exists. To date, ships visiting U.S. ports are required to submit ballast water management reports; however, many other countries do not collect this information. In fact, the predictions presented in this paper are incomplete as Canada does not require the reporting of ballast water discharge events for ships that only travel within Canadian waters, and any ballast water data that is collected is not readily available [5]. Without this data, we were unable to predict the spread of each species along the Canadian coast. Even those Canadian ports for which we made some prediction of risk do not consider potential sources of propagules from other Canadian locations; therefore, do not provide an appropriate level of guidance for managing invasive species in the Canadian Great Lakes. If governing units are to make sound decisions about ballast water management, it is important that this information be made available in the future.

A further limitation to the model we have described here is the lack of rigorous occurrence data for invasive species. There is a tendency for aquatic species occurrence records to only be collected in port and marina locations; however, the spread of occurrences that we obtained from the NAS database are not limited to these areas, though some port bias may exist (Figures 1 and 2) [39]. However, our goal was to identify the spread of invasive species due to ballast water alone. With this in mind, we were able to attribute a large portion of species occurrences using ballast water as the lone long-distance vector of dispersal. There is potential that other vectors of spread may contribute to the infestation of an area; however, ballast water would always serve as a potential disperser regardless of how the species was actually introduced to a port. We hypothesize that the larger issue with the data is the lack of timely detection, as illustrated by the spread of zebra mussel and VHSV [7], and the trend of not reporting absences. Because of these issues, it is difficult to fully capture the pattern of spread of an invasive species. We expect that our ballast water model will help to improve monitoring of secondary spread within the basin, and improved dispersal occurrence data should, in turn, enable model re-calibration and more accurate predictions.

The creation of a dynamic, spatial model simulating the secondary spread of invasive species due to ballast water in the Great Lakes has allowed us to identify the links between ballast water source and discharge locations. This information is already informing invasive species managers and policy-makers, motivating surveillance efforts, and illustrating the need to proactively manage ballast water to prevent or slow the spread of current and future invaders. With the model predictions for Eurasian Ruffe, we were able to identify the most likely locations where this invasive fish will invade next. For golden mussel and killer shrimp, we show that prevention is still the best policy for these species, as they both are expected to spread rapidly upon invasion. Also, given surveillance limitations, proactive management of intra-basin movement of ballast water is advisable if there is to be any hope that a new invader can be contained and eradicated.

## Supporting Information

Information S1 **Model Code.** Included is the Python code for the models used to backcast the spread of Eurasian Ruffe and zebra mussel and to forecast the spread of Eurasian Ruffe, killer shrimp, and golden mussel. The code has been modified so as to include generic file names. The random and location models both include code from a random selection tool (RandomSelection.tbx) that was downloaded from the ESRI website: http://arcscripts.esri.com/details.asp?dbid=15441 (last accessed: 3/29/2011) and modified.
(PDF)

Information S2 **Prediction Maps.** Included are the resulting predictions modeled for Eurasian Ruffe, killer shrimp, and golden mussel. Ten time-steps were modeled from each of the invasion start locations for each species. Results are also included for both sets of parameter values used to predict the future spread of Eurasian Ruffe. Killer shrimp spread predictions were not modeled from Superior, Wisconsin, USA due to its proximity to Duluth, Minnesota, USA.
(PDF)
