# Supplementary material for: A Spatial Modeling Approach to Predicting the Secondary Spread of Invasive Species Due to Ballast Water Discharge
Source: PLoS One. 2014 Dec 3;9(12):e114217. doi: 10.1371/journal.pone.0114217 (PMC4254998; doi:10.1371/journal.pone.0114217)
Supplement: Information S1 — Model Code. Included is the Python code for the models used to backcast the spread of Eurasian Ruffe and zebra mussel and to forecast the spread of Eurasian Ruffe, killer shrimp, and golden mussel. The code has been modified so as to include generic file names. The random and location models both include code from a random selection tool (RandomSelection.tbx) that was downloaded from the ESRI website: http://arcscripts.esri.com/details.asp?dbid=15441 (last accessed: 3/29/2011) and modified. (PDF) [file pone.0114217.s001.pdf]

SUPPORTING INFORMATION 1: MODEL CODE

Following is the Python code for the models used to backcast the spread of Eurasian ruffe and zebra mussel and to forecast the spread of Eurasian ruffe, killer shrimp, and golden mussel. The code has been modified so as to include generic file names. The random and location models both include code from a random selection tool (RandomSelection.tbx) that was downloaded from the ESRI website: http://arcscripts.esri.com/details.asp?dbid=15441 (last accessed: 3/29/2011) and modified.

Table of Contents

Backcasting Models
Random Model 2
Location Model 7
Propagule Pressure Model 12

Forecasting Model
Prediction Model 17

BACKCASTING MODELS

Random Model

```
# Import the arcpy module
import arcpy, os, sys, traceback, subprocess
import random
import numpy.random
from numpy.random import poisson

# Set Geoprocessing environments
arcpy.env.scratchWorkspace = "\\scratch_data"
arcpy.env.workspace = "in_memory"
arcpy.env.overwriteOutput = True

# Script arguments
Spreaddistance = arcpy.GetParameterAsText(0)
Spreadunits = arcpy.GetParameterAsText(1)
stStartyear = arcpy.GetParameterAsText(2)
stEndyear = arcpy.GetParameterAsText(3)
Finallayer = arcpy.GetParameterAsText(4)

#Local Variables
v_scratchworkspace = "%scratchworkspace%"
randinfpoints = "\\rand_inf_points"
speciesboundary = "\\species_boundary"
iStartyear = int(stStartyear)
iEndyear = int(stEndyear)
global iyear
iyear = iStartyear
global styear
styear = str(iyear)
global inewyear
inewyear = iyear + 1
global stnewyear
stnewyear = str(inewyear)
global iyear2
iyear2 = int(stStartyear)
global styear2
styear2 = str(iyear2)
```

```
global iPreviousyear
iPreviousyear = iyear2 - 1
global stPreviousyear
stPreviousyear = str(iPreviousyear)
Bufdist = Spreaddistance + " " + Spreadunits


#Process: Clip--clip randomly generated points to species boundary
arcpy.Clip_analysis(randinfpoints, speciesboundary, "in_memory\memSpeciesRand_" + styear)

#Create a layer of random points that have not been previously selected for next year
while int(iyear) <= int(iEndyear):

    #Block of code that selects and maps random points.
    #Created from RandomSelection.py by Leah Saunders, ESRI Inc.
    #With major modification by Stephen Lead.
    ranct = numpy.random.poisson(lam=4)
    desc = arcpy.Describe("in_memory\memSpeciesRand_" + styear)
    recct = int(arcpy.GetCount_management("in_memory\memSpeciesRand_" + styear).getOutput(0))
    if ranct <= recct:
        numValues = (ranct)
    else:
        numValues = (recct)
    arcpy.AddMessage("Selecting " + str(numValues) + " random features")

    if numValues > 0:

        inList = []
        randomList = []
        fldname = desc.OIDFieldName
        rows = arcpy.SearchCursor("in_memory\memSpeciesRand_" + styear)
        row = rows.next()
        arcpy.AddMessage ("Loading all IDs into a list")
        while row:
            id = row.getValue(fldname)
            inList.append(id)
            row = rows.next()

        selpnts = 0
        arcpy.AddMessage("Creating the list of randomly selected features")
        while len(randomList) < numValues:
```

```
            selpnts += 1
            selItem = random.choice(inList)
            randomList.append(selItem)
            inList.remove(selItem)

        theLen = len(str(randomList))
        sqlexp = '"' + fldname + '"' + " in " + "(" + str(randomList)[1:theLen - 1] + ")"
        arcpy.MakeFeatureLayer_management("in_memory\memSpeciesRand_" + styear, "\\SpeciesRand_" + styear
        + "_selection.lyr", sqlexp)
        arcpy.SaveToLayerFile_management("\\SpeciesRand_" + styear + "_selection.lyr", "\\SpeciesInf_"
        + styear + ".lyr")
    else:
        arcpy.MakeFeatureLayer_management("\\false_point", "\\SpeciesInf_" + styear + ".lyr")

    #Process: Merge--merge layer of randomly selected points with fake point in case of empty layer
    arcpy.Merge_management(["\\SpeciesInf_" + styear + ".lyr", "\\false_point"], "in_memory\memSpeciesInf_"
    + styear)

    #Process: Add field--add prediction field
    arcpy.AddField_management("in_memory\memSpeciesInf_" + styear, "pred", "SHORT")

    #Process: Calculate field--calculate prediction field
    arcpy.CalculateField_management("in_memory\memSpeciesInf_" + styear, "pred", "1")

    #Process: Select--select predicted points
    arcpy.Select_analysis("in_memory\memSpeciesInf_" + styear, "in_memory\memSpeciesInf_" + styear + "_2",
    "\"pred\" = 1")

    #Process: Buffer--Buffer infested locations so they will not be re-selected
    arcpy.Buffer_analysis("in_memory\memSpeciesInf_" + styear + "_2", "in_memory\memSpeciesInf_" + styear +
    "_3", "1 Feet", "FULL", "ROUND", "ALL")

    #Process: Erase--Erase infested areas from random points layer to prevent re-selection
    arcpy.Erase_analysis("in_memory\memSpeciesRand_" + styear, "in_memory\memSpeciesInf_" + styear + "_3",
    "in_memory\memSpeciesRand_" + stnewyear)

    #Process: Delete prediction field
    arcpy.DeleteField_management("in_memory\memSpeciesRand_" + stnewyear, "pred")

    global iyear
    iyear += 1
```

```
    global styear
    styear = str(iyear)

    global inewyear
    inewyear = int(iyear + 1)

    global stnewyear
    stnewyear = str(inewyear)

fc_list = arcpy.ListFeatureClasses("memSpeciesRand*")

arcpy.AddMessage("List of Feature Classes:" + str(fc_list))

for fc in fc_list:

    #Process: Merge--merge infested points with random point in case of empty layer
    arcpy.Merge_management(["in_memory\memSpeciesInf_" + styear2 + "_2", "\\false_point"],
    "in_memory\memSpeciesInfPtAll_" + styear2)

    #Process: Buffer--buffer to create infested area
    arcpy.Buffer_analysis("in_memory\memSpeciesInfPtAll_" + styear2, "in_memory\memSpeciesPy_" + styear2,
    "1.4 Kilometers", "FULL", "ROUND", "ALL")

    if iyear2 > iStartyear:

        #Process: Union--union previous years' infestations
        arcpy.Union_analysis(["in_memory\memSpeciesPy_" + styear2, "in_memory\memSpeciesInfYes_" +
        stPreviousyear], "in_memory\memSpeciesPyAll_" + styear2, "All", "", "GAPS")

    else:

        #Process: Copy Features--create feature class to be buffered when iyear = iStartyear
        arcpy.CopyFeatures_management("in_memory\memSpeciesPy_" + styear2, "in_memory\memSpeciesPyAll_" +
        styear2)

    #Process: Buffer--buffer to simulate natural spread
    arcpy.Buffer_analysis("in_memory\memSpeciesPyAll_" + styear2, "in_memory\memSpeciesInf_" + styear2,
    Bufdist, "FULL", "ROUND", "ALL")

    #Process: Clip--clip to species boundary
```

```
arcpy.Clip_analysis("in_memory\memSpeciesInf_" + styear2, speciesboundary,
"in_memory\memSpeciesInfYes_" + styear2)

#Process: Add field--Add prediction field
arcpy.AddField_management("in_memory\memSpeciesInfYes_" + styear2, "pred", "SHORT")

#Process: Calculate field--Calculate prediction field
arcpy.CalculateField_management("in_memory\memSpeciesInfYes_" + styear2, "pred", "1")

#Process: Identity--Combine prediction results and actual data
arcpy.Identity_analysis("\\species_" + styear2, "in_memory\memSpeciesInfYes_" + styear2,
"in_memory\memSpeciesPred_" + styear2, "ALL")

#Process: Add field--Add iteration count field
arcpy.AddField_management("in_memory\memSpeciesPred_" + styear2, "Iter", "LONG")

#Process: Calculate field--Calculate iteration count
arcpy.CalculateField_management("in_memory\memSpeciesPred_" + styear2, "Iter", "%n%")

#Process: Append--Append results to final layer
arcpy.Append_management("in_memory\memSpeciesPred_" + styear2, Finallayer, "NO_TEST")

global iyear2
iyear2 += 1
iyear2 = int(iyear2)

global styear2
styear2 = str(iyear2)

global iPreviousyear
iPreviousyear = int(iyear2 - 1)

global stPreviousyear
stPreviousyear = str(iPreviousyear)
```

Location Model

```
# Import the arcpy module
import arcpy, os, sys, traceback, subprocess
import random
import numpy.random
from numpy.random import poisson

# Set Geoprocessing environments
arcpy.env.scratchWorkspace = "\\scratch_data"
arcpy.env.workspace = "in_memory"
arcpy.env.overwriteOutput = True

# Script arguments
Spreaddistance = arcpy.GetParameterAsText(0)
Spreadunits = arcpy.GetParameterAsText(1)
stStartyear = arcpy.GetParameterAsText(2)
stEndyear = arcpy.GetParameterAsText(3)
Finallayer = arcpy.GetParameterAsText(4)

# Local Variables
gldischarge = "\\gl_discharge"
speciesboundary = "\\species_boundary"
iStartyear = int(stStartyear)
iEndyear = int(stEndyear)
global iyear
iyear = iStartyear
global styear
styear = str(iyear)
global inewyear
inewyear = iyear + 1
global stnewyear
stnewyear = str(inewyear)
global iyear2
iyear2 = int(stStartyear)
global styear2
styear2 = str(iyear2)
rand_point = "\\rand_point"
global iPreviousyear
iPreviousyear = iyear2 - 1
global stPreviousyear
```

```
stPreviousyear = str(iPreviousyear)
Bufdist = Spreaddistance + " " + Spreadunits

#Process: Clip--clip discharge data to species boundary
arcpy.Clip_analysis(gldischarge, speciesboundary, "in_memory\memSpeciesDischarge_" + styear)

#Create a discharge location of points that have not been previously selected for next year
while int(iyear) <= int(iEndyear):

    #Block of code that selects and maps random points.
    #Created from RandomSelection.py by Leah Saunders, ESRI Inc.
    #With major modification by Stephen Lead.
    ranct = numpy.random.poisson(lam=4)
    desc = arcpy.Describe("in_memory\memSpeciesDischarge_" + styear)
    recct = int(arcpy.GetCount_management("in_memory\memSpeciesDischarge_" + styear).getOutput(0))
    if ranct <= recct:
        numValues = (ranct)
    else:
        numValues = (recct)
    arcpy.AddMessage("Selecting " + str(numValues) + " random features")

    if numValues > 0:

        inList = []
        randomList = []
        fldname = desc.OIDFieldName
        rows = arcpy.SearchCursor("in_memory\memSpeciesDischarge_" + styear)
        row = rows.next()
        arcpy.AddMessage ("Loading all IDs into a list")
        while row:
            id = row.getValue(fldname)
            inList.append(id)
            row = rows.next()

        selpnts = 0
        arcpy.AddMessage("Creating the list of randomly selected features")
        while len(randomList) < numValues:
            selpnts += 1
            selItem = random.choice(inList)
            randomList.append(selItem)
            inList.remove(selItem)
```

```
    theLen = len(str(randomList))
    sqlexp = '"' + fldname + '"' + " in " + "(" + str(randomList)[1:theLen - 1] + ")"
    arcpy.MakeFeatureLayer_management("in_memory\memSpeciesDischarge_" + styear,
    "\\species_discharge_" + styear + "_selection.lyr", sqlexp)
    arcpy.SaveToLayerFile_management("\\species_discharge_" + styear + "_selection.lyr",
    "\\species_inf_" + styear + ".lyr")
else:
    arcpy.MakeFeatureLayer_management("\\false_point", "\\species_inf_" + styear + ".lyr")

#Process: Merge--merge with fake point in case of empty layer
arcpy.Merge_management(["\\species_inf_" + styear + ".lyr", "\\false_point.lyr"],
"in_memory\memSpeciesInf_" + styear)

#Process: Add field--add prediction field
arcpy.AddField_management("in_memory\memSpeciesInf_" + styear, "pred", "SHORT")

#Process: Calculate field--calculate prediction field
arcpy.CalculateField_management("in_memory\memSpeciesInf_" + styear, "pred", "1")

#Process: Join field--Join infected locations with the discharge locations
arcpy.JoinField_management("in_memory\memSpeciesDischarge_" + styear, "Location",
"in_memory\memSpeciesInf_" + styear, "Location", "pred")

#Process: Select--Select locations that have not been infested yet
arcpy.Select_analysis("in_memory\memSpeciesDischarge_" + styear, "in_memory\memSpeciesDischarge_" +
stnewyear, "\"pred\" IS NULL")

#Process: Delete prediction field
arcpy.DeleteField_management("in_memory\memSpeciesDischarge_" + styear, "pred")
arcpy.DeleteField_management("in_memory\memSpeciesDischarge_" + stnewyear, "pred")

global iyear
iyear += 1

global styear
styear = str(iyear)

global inewyear
inewyear = int(iyear + 1)
```

```
    global stnewyear
    stnewyear = str(inewyear)

fc_list = arcpy.ListFeatureClasses("memSpeciesDischarge*")

arcpy.AddMessage("List of Feature Classes:" + str(fc_list))

for fc in fc_list:

    #Process: Merge--merge with random point in case of empty layer
    arcpy.Merge_management(["in_memory\memSpeciesInf_" + styear2, "\\false_point"],
    "in_memory\memSpeciesInfPtAll_" + styear2)

    #Process: Buffer--buffer to create infestation area
    arcpy.Buffer_analysis("in_memory\memSpeciesInfPtAll_" + styear2, "in_memory\memSpeciesPy_" +
    styear2, "1.4 Kilometers", "FULL", "ROUND", "ALL")

    if iyear2 > iStartyear:

        #Process: Union--union previous years' infestations
        arcpy.Union_analysis(["in_memory\memSpeciesPy_" + styear2, "in_memory\memSpeciesInfYes_" +
        stPreviousyear], "in_memory\memSpeciesPyAll_" + styear2, "All", "", "GAPS")

    else:

        #Process: Copy Features--create feature class to be buffered when iyear = iStartyear
        arcpy.CopyFeatures_management("in_memory\memSpeciesPy_" + styear2, "in_memory\memSpeciesPyAll_"
        + styear2)

    #Process: Buffer--buffer to simulate natural spread
    arcpy.Buffer_analysis("in_memory\memSpeciesPyAll_" + styear2, "in_memory\memSpeciesInf_" + styear2,
    Bufdist, "FULL", "ROUND", "ALL")

    #Process: Clip--clip to species boundary
    arcpy.Clip_analysis("in_memory\memSpeciesInf_" + styear2, speciesboundary,"in_memory\memSpeciesInfYes_"
    + styear2)

    #Process: Add field--Add prediction field
    arcpy.AddField_management("in_memory\memSpeciesInfYes_" + styear2, "pred", "SHORT")

    #Process: Calculate field--Calculate prediction field
```

```
arcpy.CalculateField_management("in_memory\memSpeciesInfYes_" + styear2, "pred", "1")

#Process: Identity--Combine prediction and actual data
arcpy.Identity_analysis("\\species_" + styear2, "in_memory\memSpeciesInfYes_" + styear2,
"in_memory\memSpeciesPred_" + styear2, "ALL")

#Process: Add field--Add iteration count field
arcpy.AddField_management("in_memory\memSpeciesPred_" + styear2, "Iter", "LONG")

#Process: Calculate field--Calculate iteration count
arcpy.CalculateField_management("in_memory\memSpeciesPred_" + styear2, "Iter", "%n%")

#Process: Append--Append results to final layer
arcpy.Append_management("in_memory\memSpeciesPred_" + styear2, Finallayer, "NO_TEST")

global iyear2
iyear2 += 1
iyear2 = int(iyear2)

global styear2
styear2 = str(iyear2)

global iPreviousyear
iPreviousyear = int(iyear2 - 1)

global stPreviousyear
stPreviousyear = str(iPreviousyear)
```

Propagule Pressure Model

```
# Import the arcpy module
import arcpy, os, sys, traceback

# Set Geoprocessing environments
arcpy.env.scratchWorkspace = "\\scratch_data"
arcpy.env.workspace = “in_memory"
arcpy.env.overwriteOutput = True

# Script arguments
Spread_distance = arcpy.GetParameterAsText(0)
Spread_units = arcpy.GetParameterAsText(1)
stStart_year = arcpy.GetParameterAsText(2)
stEnd_year = arcpy.GetParameterAsText(3)
Survival = arcpy.GetParameterAsText(4)
Final_layer = arcpy.GetParameterAsText(5)

#Local Variables
v_scratchworkspace = "%scratchworkspace%"
istartyear = int(stStart_year)
iendyear = int(stEnd_year)
fsurvival = float(Survival)
discharge = "\\gl_discharge"
speciesboundary = "\\species_boundary"
speciesdischarge = "\\species_discharge"
Bufdist = Spread_distance + " " + Spread_units
speciesstartyear = "\\species_" + stStart_year
global iyear
iyear = istartyear
global styear
styear = str(iyear)
pred = "Pred"
glsource = "\\gl_discharge_source"
sourcedischargetrips= "\\source_discharge_trips"
falsepoint = "\\tb_ppfalse_point"
infvisits = "inf_visits"
global iyear
inewyear = iyear + 1
global stnewyear
```

```
stnewyear = str(inewyear)
itera = "iter"


#Process: Copy Features--Copy feature classes into memory
arcpy.CopyFeatures_management(discharge, "in_memory\memDischarge")
arcpy.CopyFeatures_management(speciesboundary, "in_memory\memSpeciesBoundary")

#Process: Clip--Clip discharge locations to species boundary
arcpy.Clip_analysis("in_memory\memDischarge", "in_memory\memSpeciesBoundary",
"in_memory\memSpeciesDischarge")

#Process: Select--Select initial species locations
arcpy.Select_analysis(speciesstartyear, "in_memory\memSpeciesInitial", "\"Actual\" = 1")

#Process: Buffer--Buffer initial infestation area
arcpy.Buffer_analysis("in_memory\memSpeciesInitial", "in_memory\memSpeciesInit_" + styear, "1.4
Kilometers", "FULL", "ROUND", "ALL")

#Process: Buffer--Buffer to simulate natural spread
arcpy.Buffer_analysis("in_memory\memSpeciesInit_" + styear, "in_memory\memSpeciesPy_" + styear, Bufdist,
"FULL", "ROUND", "ALL")

#Process: Clip--Clip to species boundary
arcpy.Clip_analysis("in_memory\memSpeciesPy_" + styear, speciesboundary, "in_memory\memSpeciesInf_" +
styear)

#Process: Add field--Add prediction field
arcpy.AddField_management("in_memory\memSpeciesInf_" + styear, "Pred", "SHORT")

#Process: Calculate field--Calculate prediction field
arcpy.CalculateField_management("in_memory\memSpeciesInf_" + styear, "Pred", "1")

global iyear
iyear = istartyear

while iyear <= iendyear:

    #Process: Identity—Identify source locations within infestation areas
    arcpy.Identity_analysis(glsource, "in_memory\memSpeciesInf_" + styear, "in_memory\memSourceInf_" +
    styear)
```

```
#Process: Merge--Merge false point in case of empty feature class
arcpy.Merge_management(["in_memory\memSourceInf_" + styear, "\\false_point"],
"in_memory\memSourceInf2_" + styear)

#Process: Select--Select infested sources
arcpy.Select_analysis("in_memory\memSourceInf2_" + styear, "in_memory\memSourceYes_" + styear,
'\"Pred\" = 1')

#Process: Join field--Join frequency fields
arcpy.JoinField_management(sourcedischargetrips, "Source_Location", "in_memory\memSourceYes_" + styear,
"Location", ["Location", "Pred"])

#Process: Table Select--Select source locations that are infested
arcpy.TableSelect_analysis(sourcedischargetrips, "in_memory\memSpeciesInfSource_" + styear,
'\"Location\" Is Not NULL')

#Process: Delete Field--Delete prediction field
arcpy.DeleteField_management(sourcedischargetrips, ["Location", "Pred"])

#Process: Add field--Add survival field
arcpy.AddField_management("in_memory\memSpeciesInfSource_" + styear, "surv_rate", "DOUBLE")

#Process: Calculate field--Calculate survival field
arcpy.CalculateField_management("in_memory\memSpeciesInfSource_" + styear, "surv_rate", fsurvival)

#Process: Add field--Add trip survival field
arcpy.AddField_management("in_memory\memSpeciesInfSource_" + styear, "trip_surv", "DOUBLE")

#Process: Calculate field--Calculate trip survival
arcpy.CalculateField_management("in_memory\memSpeciesInfSource_" + styear, "trip_surv",
"[surv_rate]^[trip_med]")

#Process: Add field--Add infestation field to discharge locations
arcpy.AddField_management("in_memory\memSpeciesInfSource_" + styear, "inf_discharge", "LONG")

#Process: Table Select--Select discharge locations that received visits
arcpy.TableSelect_analysis("in_memory\memSpeciesInfSource_" + styear,
"in_memory\memSpeciesInfDischarge_" + styear, '"trip_countperyear" > 0')

#Process: Calculate field--Determine infestation status for each trip
```

```
arcpy.CalculateField_management("in_memory\memSpeciesInfDischarge_" + styear, "inf_discharge",
"numpy.random.binomial(n=!trip_countperyear!, p=!trip_surv!)", "PYTHON_9.3",
"import numpy.random\nfrom numpy.random import binomial")

#Process: Table Select--Select infested discharge locations
arcpy.TableSelect_analysis("in_memory\memSpeciesInfDischarge_" + styear,
"in_memory\memSpeciesInfNewDis_" + styear, '"inf_discharge" > 0')

#Process: Add field--Add prediction field
arcpy.AddField_management("in_memory\memSpeciesInfNewDis_" + styear, "Pred", "SHORT")

#Process: Calculate field--Calculate prediction field
arcpy.CalculateField_management("in_memory\memSpeciesInfNewDis_" + styear, "Pred", "1")

#Process: Join field--Join source and discharge locations
arcpy.JoinField_management("in_memory\memSpeciesDischarge", "Location",
"in_memory\memSpeciesInfNewDis_" + styear, "Discharge_Location", ["Discharge_Location", "Pred"])

#Process: Select--Select infested points
arcpy.Select_analysis("in_memory\memSpeciesDischarge", "in_memory\memSpeciesDischargeYes_" + styear,
'\"Discharge_Location\" Is Not NULL')

#Process: Delete field--Delete fields from join above
arcpy.DeleteField_management("in_memory\memSpeciesDischarge", ["Discharge_Location", "Pred"])

#Process: Buffer--Buffer to create infestation area
arcpy.Buffer_analysis("in_memory\memSpeciesDischargeYes_" + styear, "in_memory\memSpeciesInit_" +
stnewyear, "1.4 Kilometers", "FULL", "ROUND", "LIST", "Pred")

#Process: Select--Select infested areas
arcpy.Select_analysis("in_memory\memSpeciesInit_" + stnewyear, "in_memory\memSpeciesPred_" + stnewyear,
'\"Pred\" = 1')

#Process: Union--Union to previous year of infestation
arcpy.Union_analysis(["in_memory\memSpeciesPred_" + stnewyear, "in_memory\memSpeciesInf_" + styear],
"in_memory\memSpeciesPredYes_" + stnewyear, "ALL", "", "GAPS")

#Process: Buffer--Buffer with spread distance
arcpy.Buffer_analysis("in_memory\memSpeciesPredYes_" + stnewyear, "in_memory\memSpeciesPy_" +
stnewyear, Bufdist, "FULL", "ROUND", "ALL")
```

```
#Process: Clip--Clip to species boundary
arcpy.Clip_analysis("in_memory\memSpeciesPy_" + stnewyear, "in_memory\memSpeciesBoundary",
"in_memory\memSpeciesInf_" + stnewyear)

#Process: Add field--Add prediction field
arcpy.AddField_management("in_memory\memSpeciesInf_" + stnewyear, "Pred", "SHORT")

#Process: Calculate field--Calculate prediction field
arcpy.CalculateField_management("in_memory\memSpeciesInf_" + stnewyear, "Pred", "1")

#Process: Identity--Combine prediction results with actual data
arcpy.Identity_analysis("\\species_" + stnewyear, "in_memory\memSpeciesInf_" + stnewyear,
"in_memory\memSpeciesPred_" + stnewyear)

#Process: Add field--Add iteration count field
arcpy.AddField_management("in_memory\memSpeciesPred_" + stnewyear, itera, "LONG")

#Process: Calculate field--Calculate iteration count
arcpy.CalculateField_management("in_memory\memSpeciesPred_" + stnewyear, itera, "%n% + 99")

#Process: Append--Append to final layer
arcpy.Append_management("in_memory\memSpeciesPred_" + stnewyear, Final_layer, "NO_TEST")

global iyear
iyear += 1

global styear
styear = str(iyear)

global inewyear
inewyear = iyear + 1

global stnewyear
stnewyear = str(inewyear)
```

FORECASTING MODEL

Prediction Model

```
# Import the arcpy module
import arcpy, os, sys, traceback

# Set Geoprocessing environments
arcpy.env.scratchWorkspace = "\\scratch_data"
arcpy.env.workspace = "in_memory"

# Script arguments
Spread_distance = arcpy.GetParameterAsText(0)
Spread_units = arcpy.GetParameterAsText(1)
stStart_year = arcpy.GetParameterAsText(2)
stEnd_year = arcpy.GetParameterAsText(3)
Survival = arcpy.GetParameterAsText(4)
Final_layer = arcpy.GetParameterAsText(5)

#Local Variables
v_scratchworkspace = "%scratchworkspace%"
istartyear = int(stStart_year)
iendyear = int(stEnd_year)
fsurvival = float(Survival)
speciesinitial = "\\species_initial"
discharge = "\\gl_discharge"
speciesboundary = "\\species_boundary"
speciesdischarge = "\\species_discharge"
Bufdist = Spread_distance + " " + Spread_units
global iyear
iyear = istartyear
global styear
styear = str(iyear)
pred = "Pred"
glsource = "\\gl_discharge_source"
sourcedischargetrips= "\\source_discharge_trips"
falsepoint = "\\tb_ppfalse_point"
infvisits = "inf_visits"
global iyear
inewyear = iyear + 1
```

```
global stnewyear
stnewyear = str(inewyear)
itera = "iter"
predyear = "predyear"


#Process: Copy Features--Copy feature classes into memory
arcpy.CopyFeatures_management(discharge, "in_memory\memDischarge")
arcpy.CopyFeatures_management(speciesboundary, "in_memory\memSpeciesBoundary")

#Process: Clip--Clip discharge locations to species boundary
arcpy.Clip_analysis("in_memory\memDischarge", "in_memory\memSpeciesBoundary",
"in_memory\memSpeciesDischarge")

#Process: Buffer--Buffer initial infestation area
arcpy.Buffer_analysis(speciesinitial, "in_memory\memSpeciesInit_" + styear, "1.4 Kilometers", "FULL",
"ROUND", "ALL")

#Process: Buffer--Buffer to simulate natural spread
arcpy.Buffer_analysis("in_memory\memSpeciesInit_" + styear, "in_memory\memSpeciesPy_" + styear, Bufdist,
"FULL", "ROUND", "ALL")

#Process: Clip--Clip to species boundary
arcpy.Clip_analysis("in_memory\memSpeciesPy_" + styear, "in_memory\memSpeciesBoundary",
"in_memory\memSpeciesInf_" + styear)

#Process: Add field--Add prediction field
arcpy.AddField_management("in_memory\memSpeciesInf_" + styear, "Pred", "SHORT")

#Process: Calculate field--Calculate prediction field
arcpy.CalculateField_management("in_memory\memSpeciesInf_" + styear, "Pred", "1")

global iyear
iyear = istartyear

while iyear <= iendyear:

    #Process: Identity--Combine infestation area with ballast sources
    arcpy.Identity_analysis(glsource, "in_memory\memSpeciesInf_" + styear, "in_memory\memSourceInf_" +
    styear)
```

```
#Process: Merge--Merge false point in case of empty feature class
arcpy.Merge_management(["in_memory\memSourceInf_" + styear, "\\false_point"],
"in_memory\memSourceInf2_" + styear)

#Process: Select--Select infested sources
arcpy.Select_analysis("in_memory\memSourceInf2_" + styear, "in_memory\memSourceYes_" + styear,
'\"Pred\" = 1')

#Process: Join field--Join frequency fields
arcpy.JoinField_management(sourcedischargetrips, "Source_Location", "in_memory\memSourceYes_" + styear,
"Location", ["Location", "Pred"])

#Process: Table Select--Select source locations that are infested
arcpy.TableSelect_analysis(sourcedischargetrips, "in_memory\memSpeciesInfSource_" + styear,
'\"Location\" Is Not NULL')

#Process: Delete Field--Delete prediction field
arcpy.DeleteField_management(sourcedischargetrips, ["Location", "Pred"])

#Process: Add field--Add survival field
arcpy.AddField_management("in_memory\memSpeciesInfSource_" + styear, "surv_rate", "DOUBLE")

#Process: Calculate field--Calculate trip survival field
arcpy.CalculateField_management("in_memory\memSpeciesInfSource_" + styear, "surv_rate", fsurvival)

#Process: Add field--Add survival field
arcpy.AddField_management("in_memory\memSpeciesInfSource_" + styear, "trip_surv", "DOUBLE")

#Process: Calculate field--Calculate trip survival
arcpy.CalculateField_management("in_memory\memSpeciesInfSource_" + styear, "trip_surv",
"[surv_rate]^[trip_med]")

#Process: Add field--Add discharge infestation field
arcpy.AddField_management("in_memory\memSpeciesInfSource_" + styear, "inf_discharge", "LONG")

#Process: Table Select--Select discharge locations that received visits
arcpy.TableSelect_analysis("in_memory\memSpeciesInfSource_" + styear,
"in_memory\memSpeciesInfDischarge_" + styear, '"trip_countperyear" > 0')

#Process: Calculate field--Calculate infestation status for each trip
arcpy.CalculateField_management("in_memory\memSpeciesInfDischarge_" + styear, "inf_discharge",
```

```
"numpy.random.binomial(n=!trip_countperyear!, p=!trip_surv!)", "PYTHON_9.3",
"import numpy.random\nfrom numpy.random import binomial")

#Process: Table Select--Select infested discharge locations
arcpy.TableSelect_analysis("in_memory\memSpeciesInfDischarge_" + styear,
"in_memory\memSpeciesInfNewDis_" + styear, '"inf_discharge" > 0')

#Process: Add field--Add prediction field
arcpy.AddField_management("in_memory\memSpeciesInfNewDis_" + styear, "Pred", "SHORT")

#Process: Calculate field--Calculate prediction field
arcpy.CalculateField_management("in_memory\memSpeciesInfNewDis_" + styear, "Pred", "1")

#Process: Join field--Join source and discharge locations
arcpy.JoinField_management("in_memory\memSpeciesDischarge", "Location",
"in_memory\memSpeciesInfNewDis_" + styear, "Discharge_Location", ["Discharge_Location", "Pred"])

#Process: Select--Select infested points
arcpy.Select_analysis("in_memory\memSpeciesDischarge", "in_memory\memSpeciesDischargeYes_" + styear,
'\"Discharge_Location\" Is Not NULL')

#Process: Delete field--Delete fields from above
arcpy.DeleteField_management("in_memory\memSpeciesDischarge", ["Discharge_Location", "Pred"])

#Process: Buffer--Buffer to create infestation area
arcpy.Buffer_analysis("in_memory\memSpeciesDischargeYes_" + styear, "in_memory\memSpeciesInit_" +
stnewyear, "1.4 Kilometers", "FULL", "ROUND", "LIST", "Pred")

#Process: Select--Select infected areas
arcpy.Select_analysis("in_memory\memSpeciesInit_" + stnewyear, "in_memory\memSpeciesPred_" + stnewyear,
'\"Pred\" = 1')

#Process: Union--Union to previous year of infestation
arcpy.Union_analysis(["in_memory\memSpeciesPred_" + stnewyear, "in_memory\memSpeciesInf_" + styear],
"in_memory\memSpeciesPredYes_" + stnewyear, "ALL", "", "GAPS")

#Process: Buffer--Buffer with spread distance
arcpy.Buffer_analysis("in_memory\memSpeciesPredYes_" + stnewyear, "in_memory\memSpeciesPy_" +
stnewyear, Bufdist, "FULL", "ROUND", "ALL")

#Process: Clip--Clip to species boundary
```

```
    arcpy.Clip_analysis("in_memory\memSpeciesPy_" + stnewyear, "in_memory\memSpeciesBoundary",
    "in_memory\memSpeciesInf_" + stnewyear)

    #Process: Add field--Add prediction field
    arcpy.AddField_management("in_memory\memSpeciesInf_" + stnewyear, "Pred", "SHORT")

    #Process: Calculate field--Calculate prediction field
    arcpy.CalculateField_management("in_memory\memSpeciesInf_" + stnewyear, "Pred", "1")

    #Process: Identity--Combine with prediction results with actual data
    arcpy.Identity_analysis("in_memory\memSpeciesDischarge", "in_memory\memSpeciesInf_" + stnewyear,
    "in_memory\memSpeciesPred_" + stnewyear)

    #Process: Add field--Add iteration count field
    arcpy.AddField_management("in_memory\memSpeciesPred_" + stnewyear, itera, "LONG")

    #Process: Calculate field--Calculate iteration count
    arcpy.CalculateField_management("in_memory\memSpeciesPred_" + stnewyear, itera, "%n%")

    #Process: Add field--Add year field
    arcpy.AddField_management ("in_memory\memSpeciesPred_" + stnewyear, predyear, "LONG")

    #Process: Calculate field--Calculate year
    arcpy.CalculateField_management ("in_memory\memSpeciesPred_" + stnewyear, predyear, iyear)

    #Process: Append--Append to final layer
    arcpy.Append_management("in_memory\memSpeciesPred_" + stnewyear, Final_layer, "NO_TEST")

    global iyear
    iyear += 1

    global styear
    styear = str(iyear)

    global inewyear
    inewyear = iyear + 1

    global stnewyear
    stnewyear = str(inewyear)

else:
```

```
print iendyear
```
