# Supplementary material for: A Spatial Modeling Approach to Predicting the Secondary Spread of Invasive Species Due to Ballast Water Discharge
Source: PLoS One. 2014 Dec 3;9(12):e114217. doi: 10.1371/journal.pone.0114217 (PMC4254998; doi:10.1371/journal.pone.0114217)

SUPPORTING INFORMATION 2: PREDICTION MAPS

Following are the resulting predictions modeled for Eurasian ruffe, killer shrimp, and golden mussel. Ten time-steps were modeled from each of the invasion start locations for each species. Results are also included for both sets of parameter values used to predict the future spread of Eurasian ruffe. Killer shrimp spread predictions were not modeled from Superior, Wisconsin due to its proximity to Duluth, Minnesota.

Table of Contents

Eurasian Ruffe: 10-km, 0.01	2
Eurasian Ruffe: 25-km, 0.0001	7
Killer Shrimp: Duluth, MN, USA	12
Killer Shrimp: Toledo, OH, USA	17
Killer Shrimp: Ogdensburg, NY, USA	22
Killer Shrimp: Green Bay, WI, USA	27
Killer Shrimp: Goderich, ON, Canada	32
Killer Shrimp: Detroit, MI, USA	37
Golden Mussel: Bay City, MI, USA	42
Golden Mussel: Duluth, MI, USA	47

Eurasian Ruffe
Dispersal Distance = 10-km and Probability of Infestation = 0.01

Time-step 1

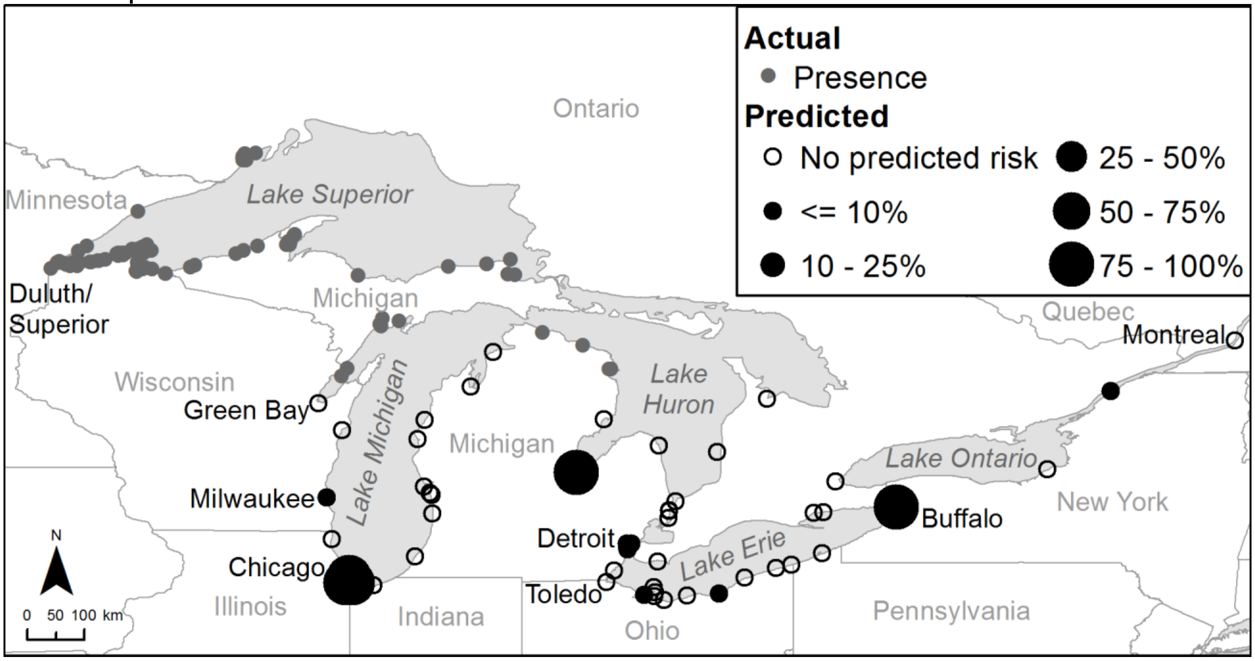

Time-step 2

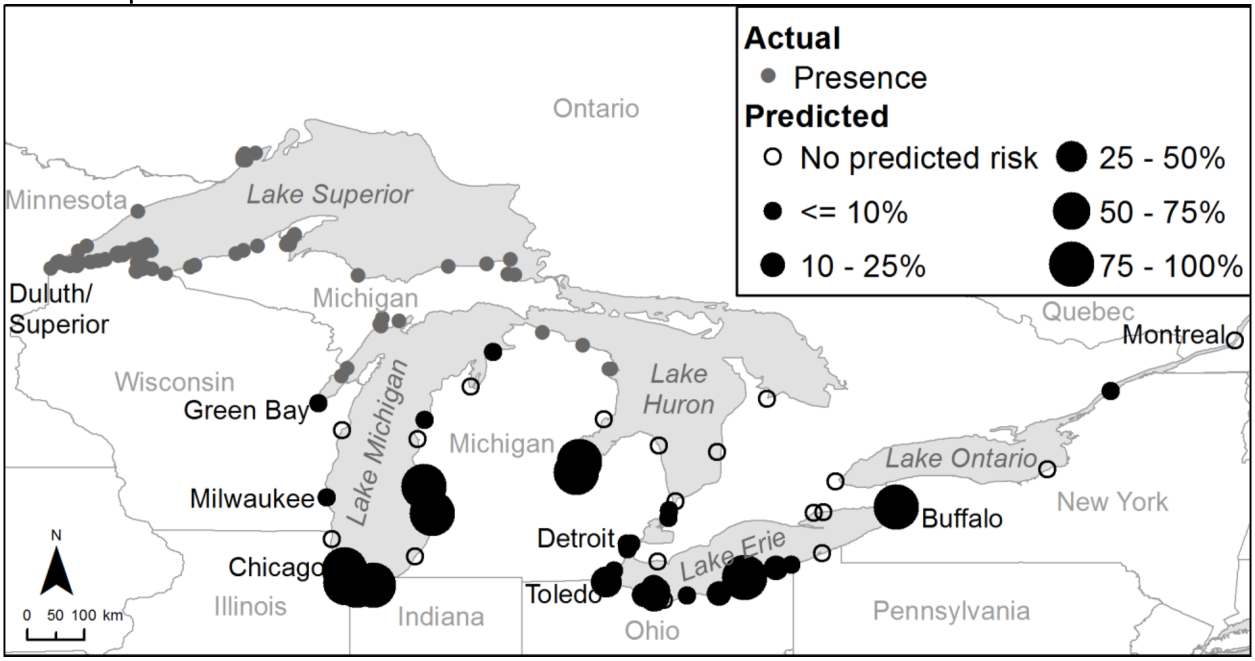

Time-step 3

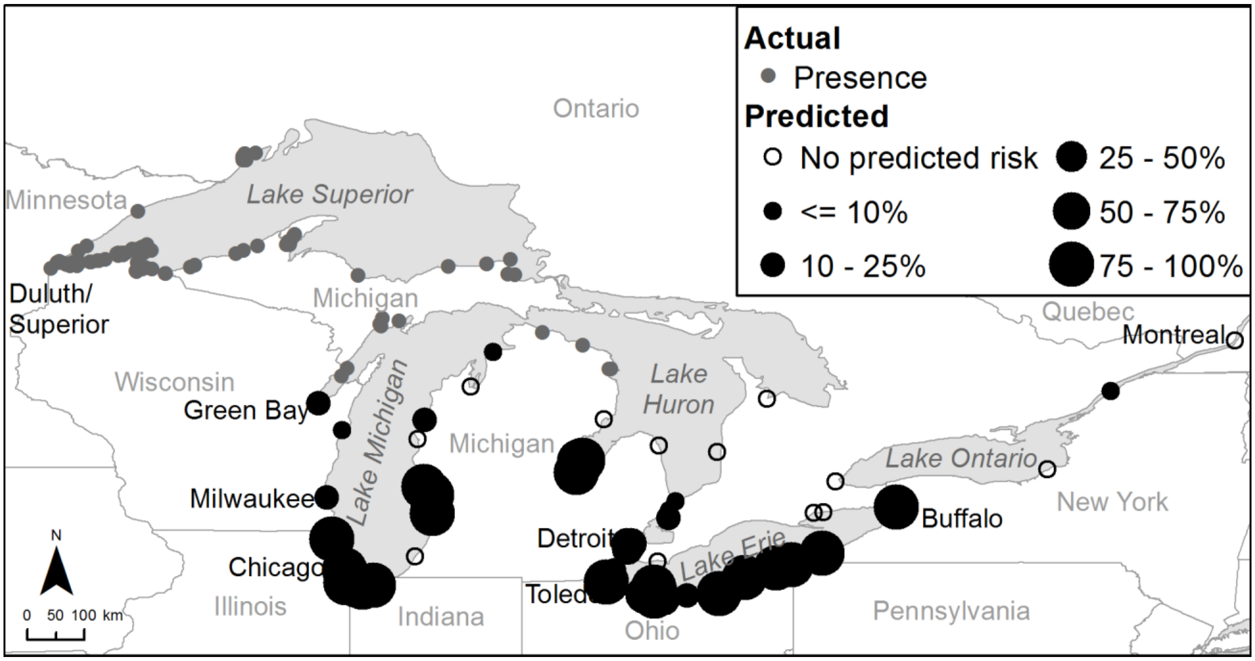

Time-step 4

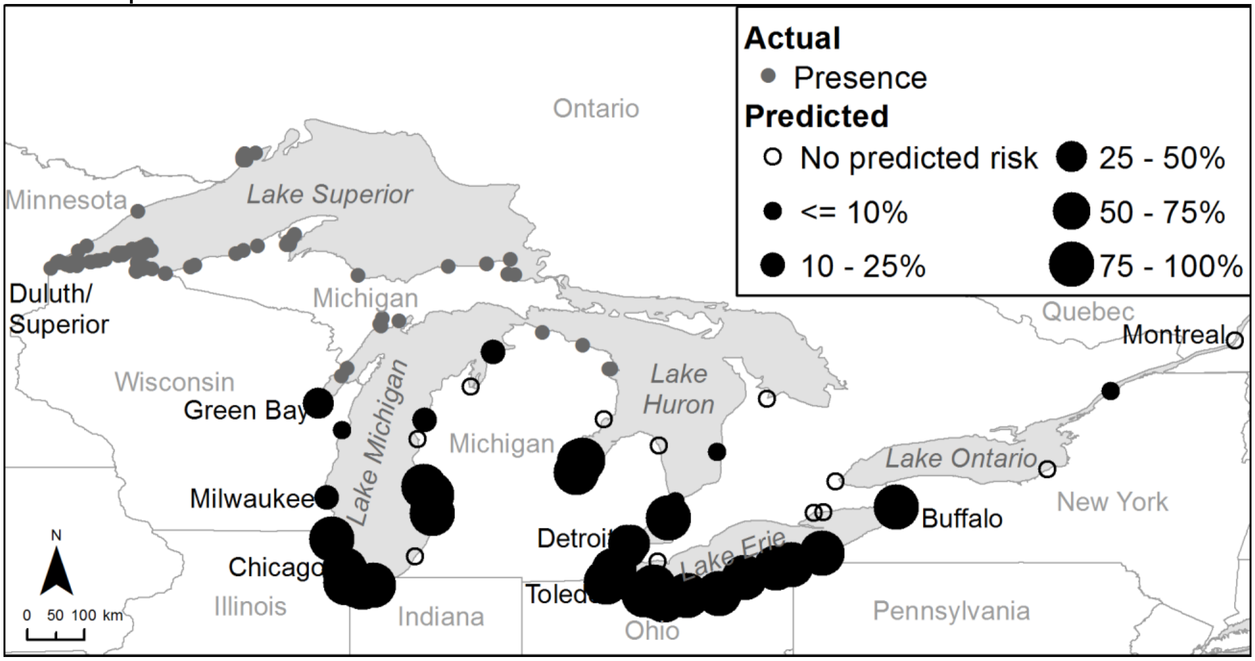

Time-step 5

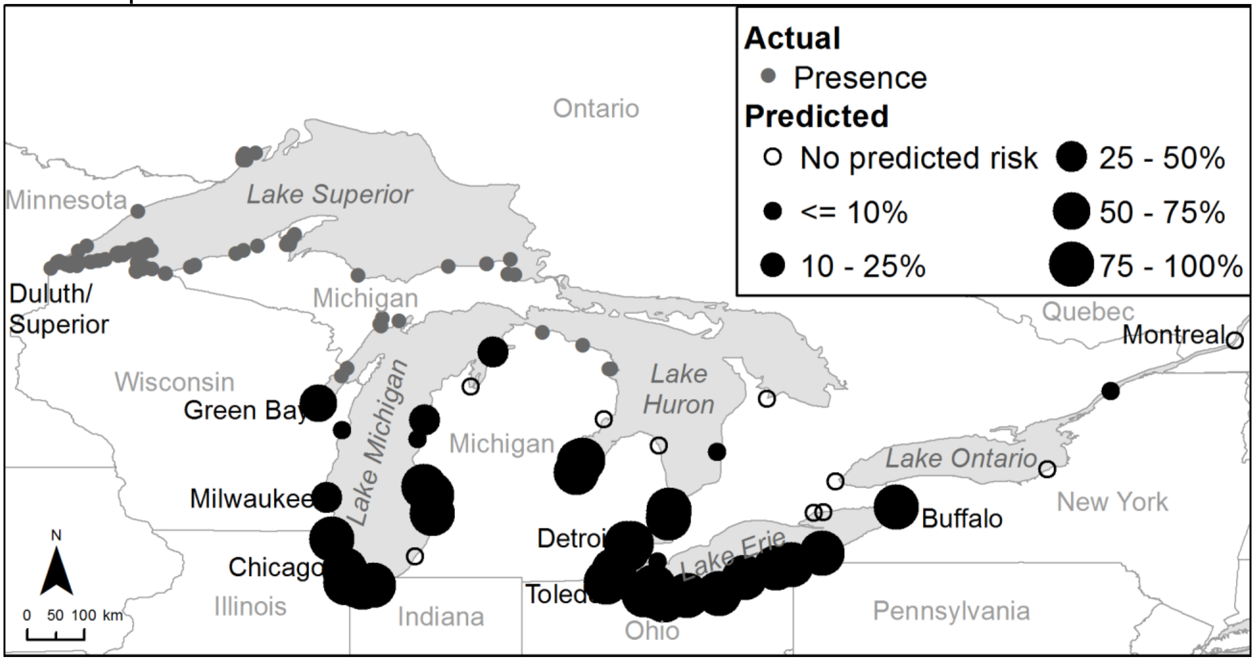

Time-step 6

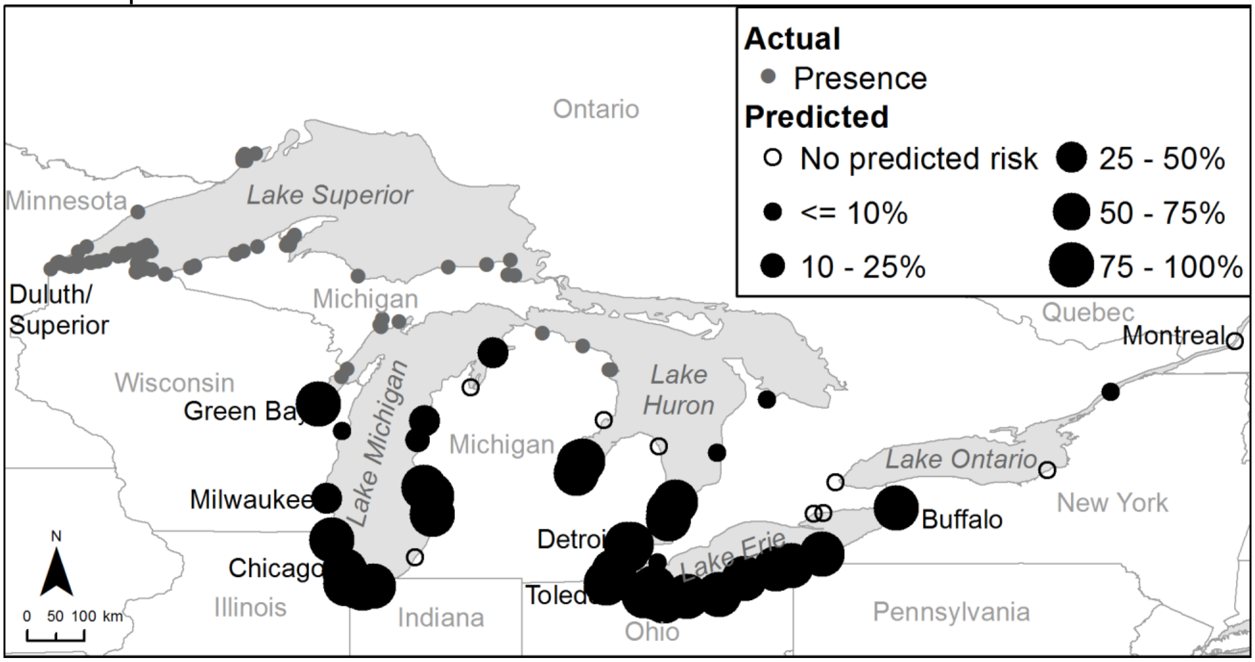

Time-step 7

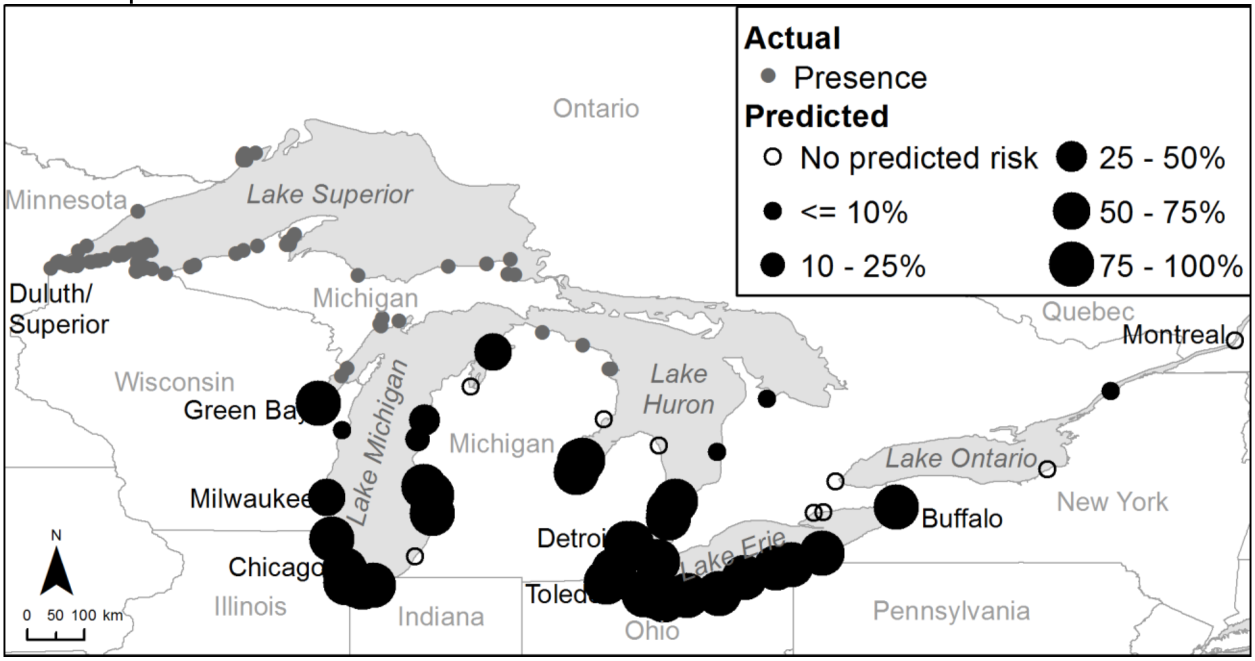

Time-step 8

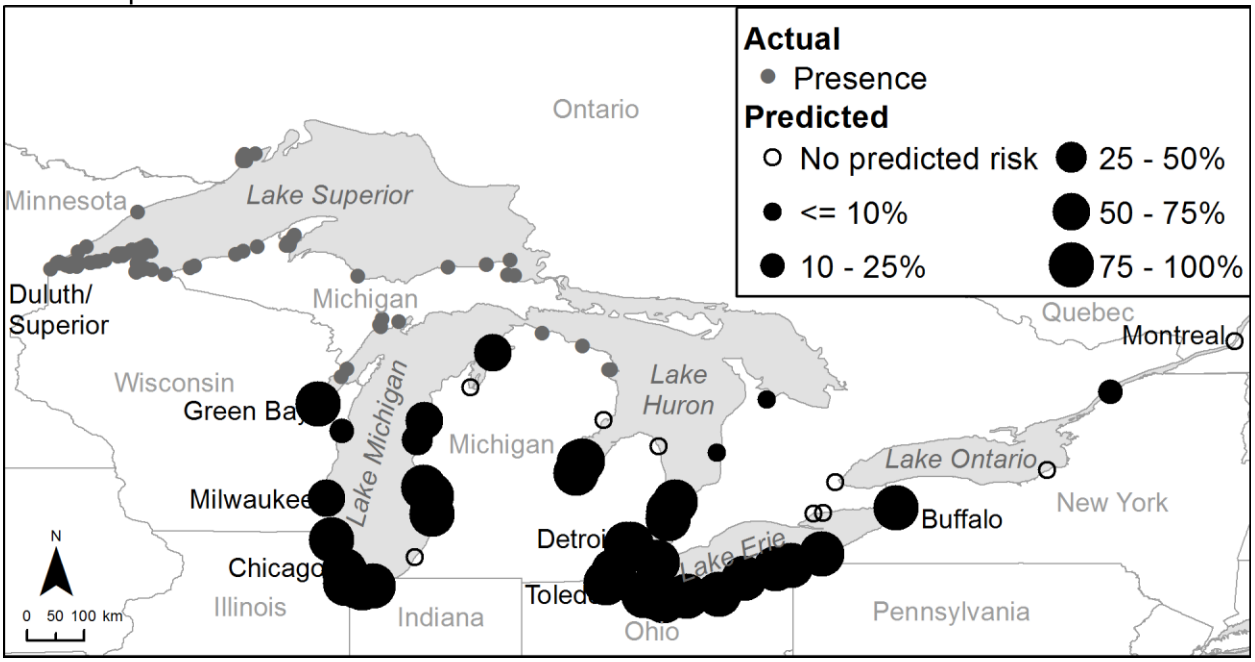

Time-step 9

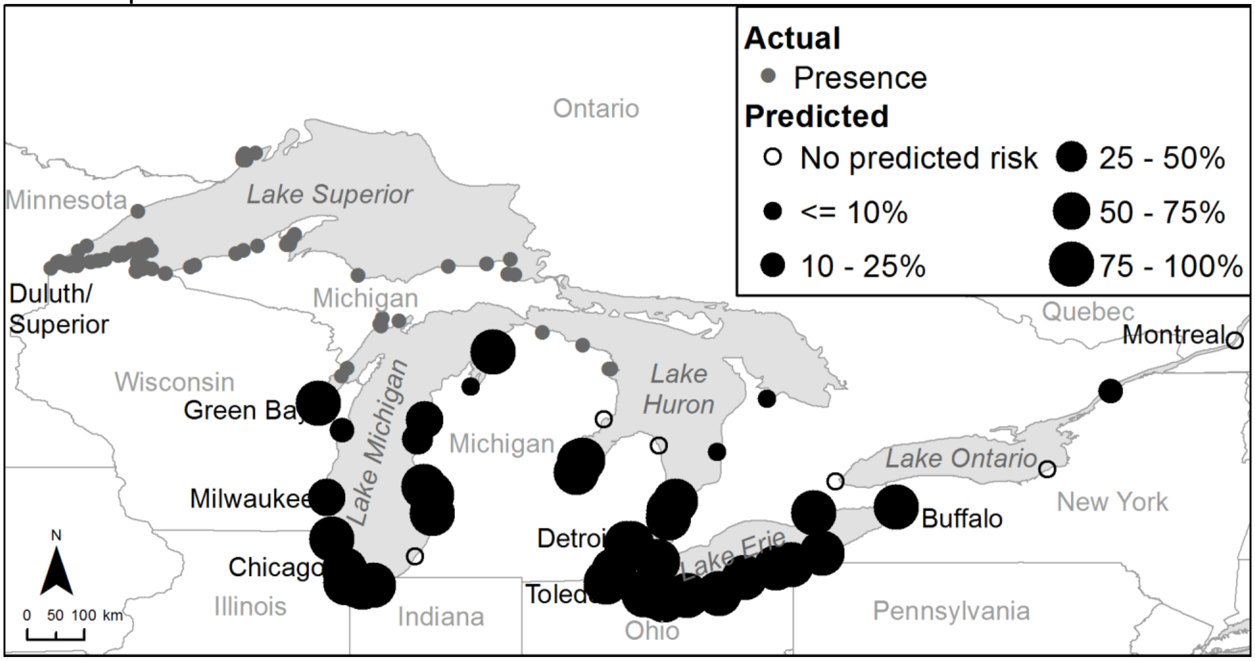

Time-step 10

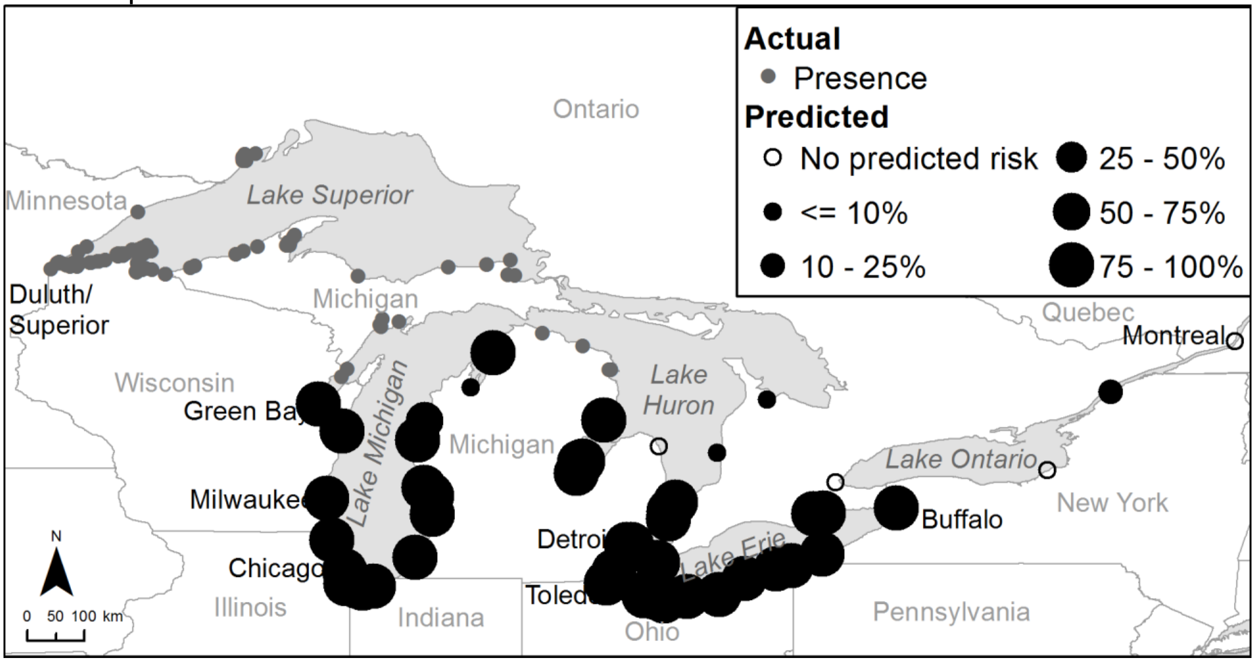

Eurasian Ruffe
Dispersal Distance = 25-km and Probability of Infestation = 0.0001

Time-step 1

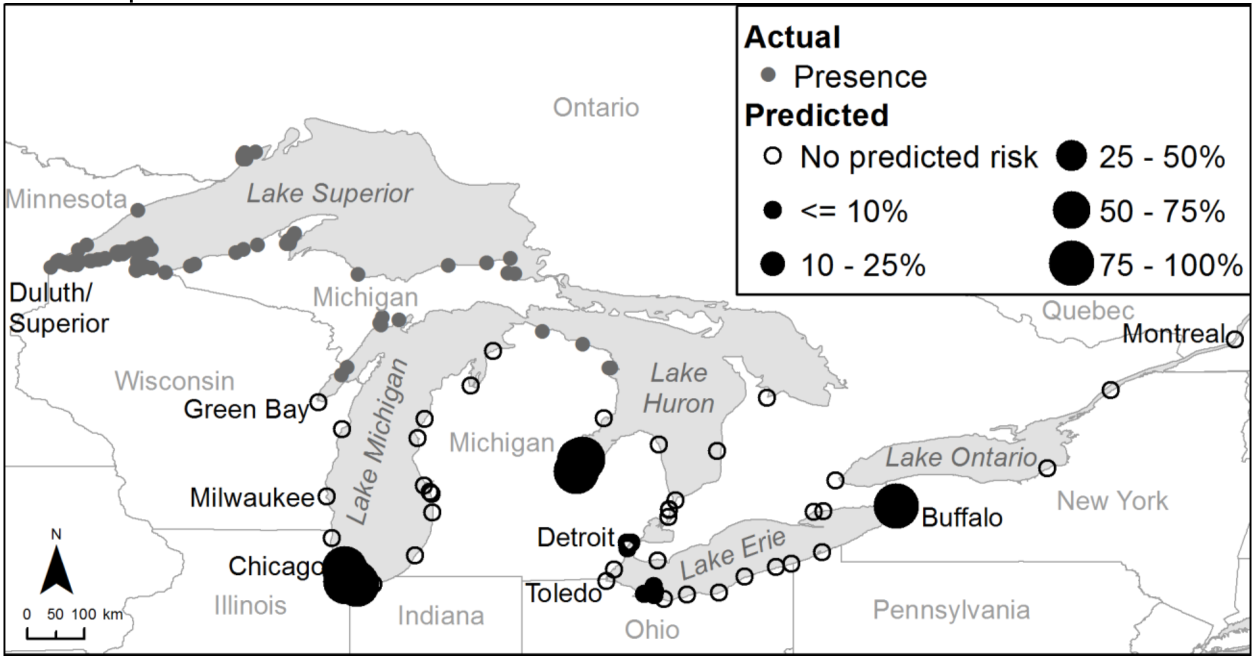

Time-step 2

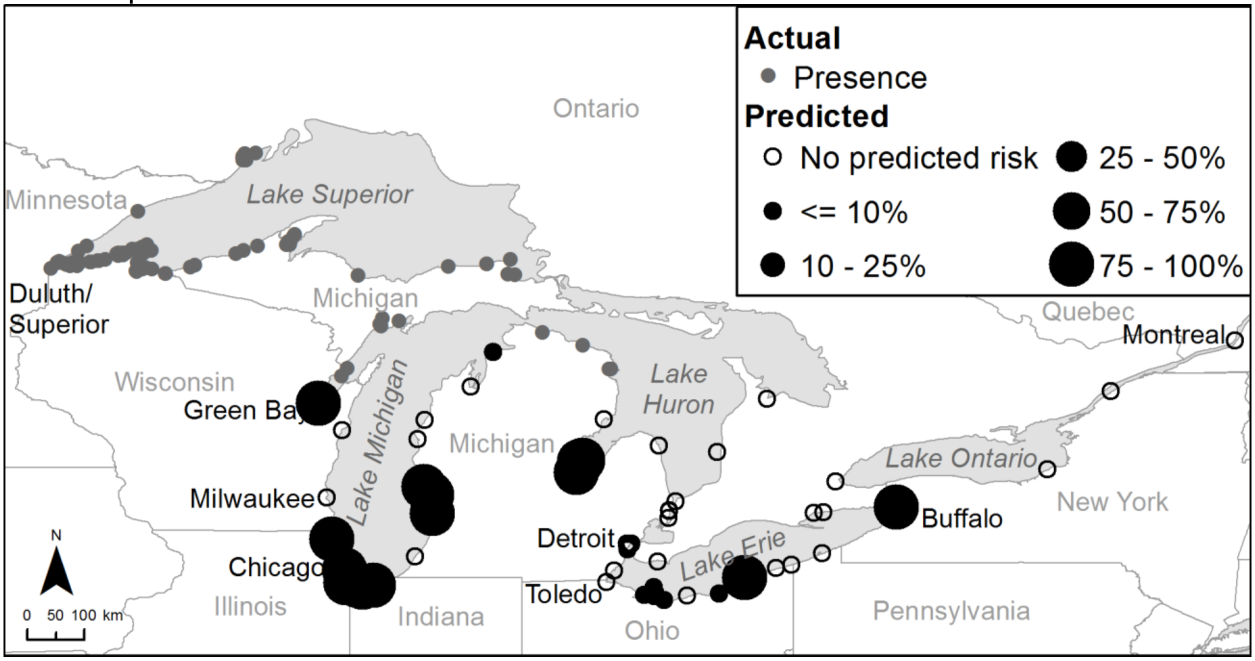

Time-step 3

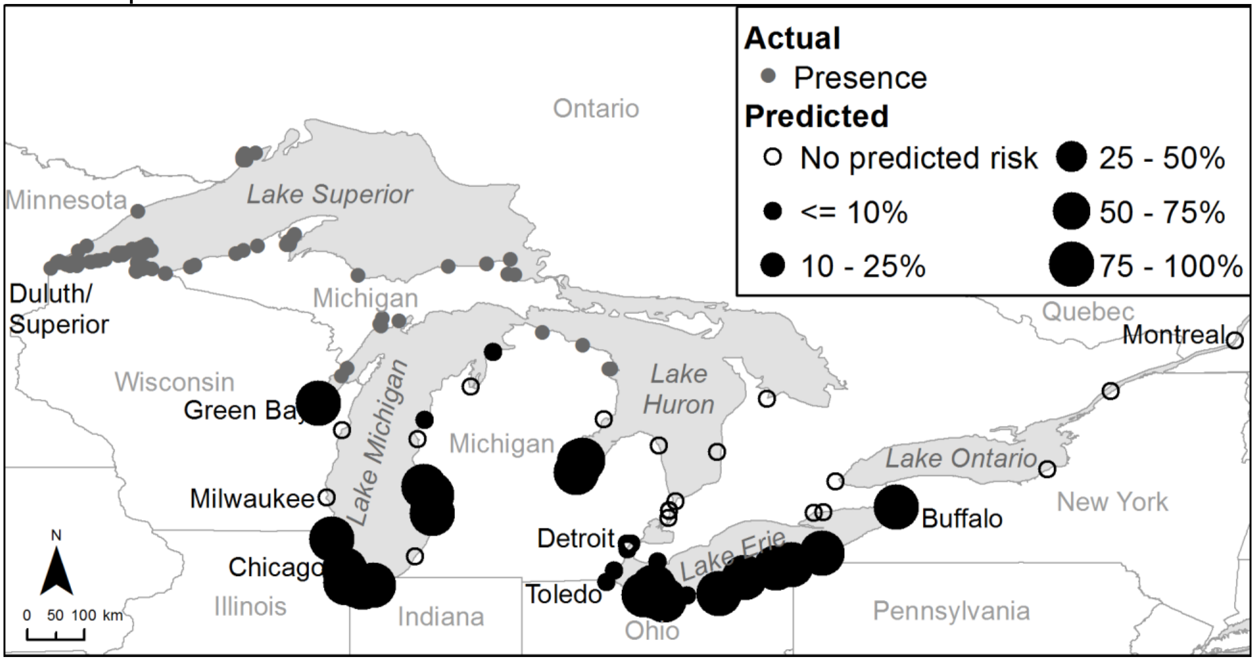

Time-step 4

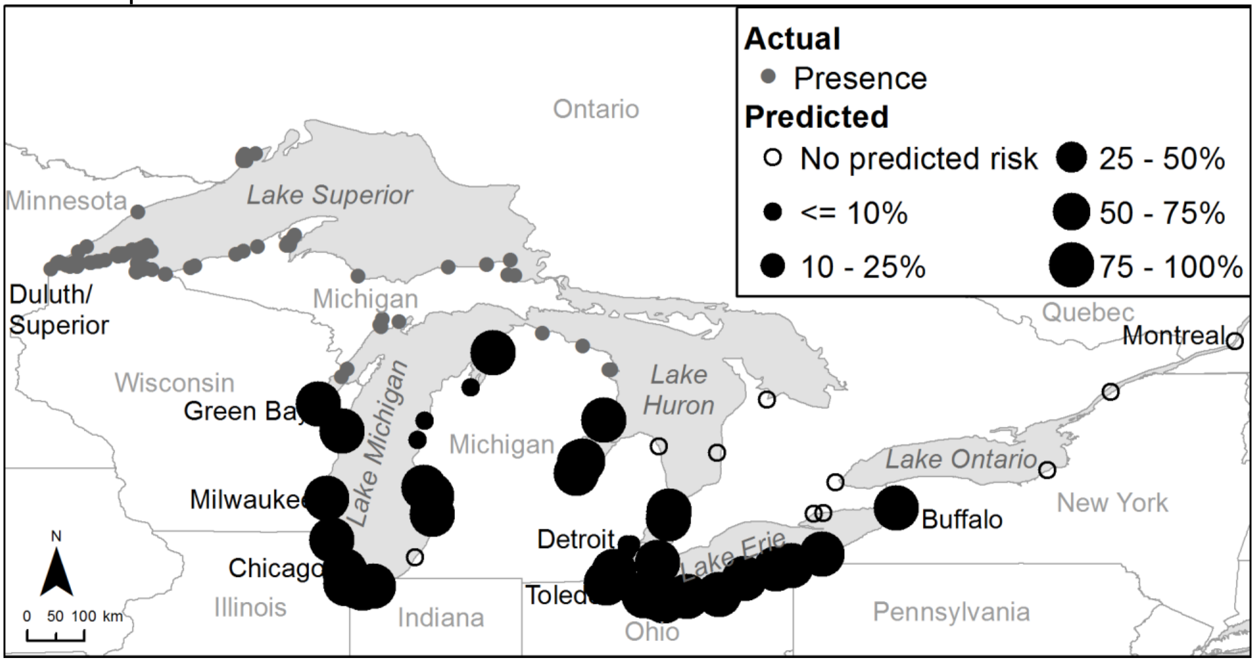

Time-step 5

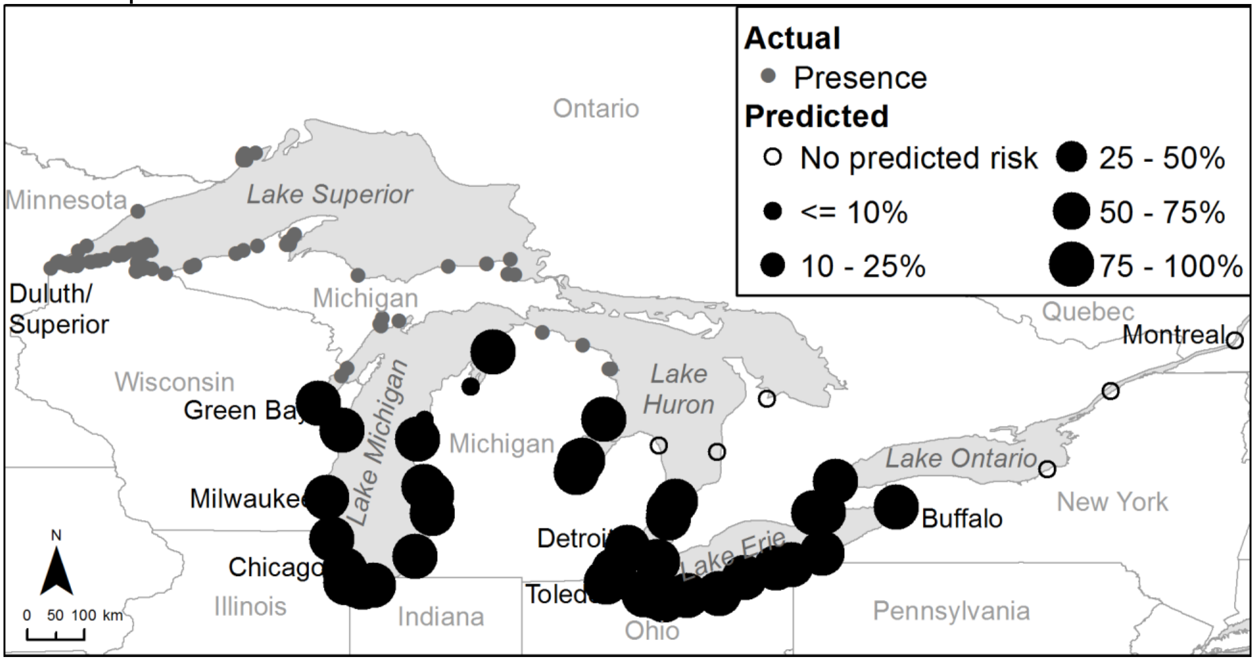

Time-step 6

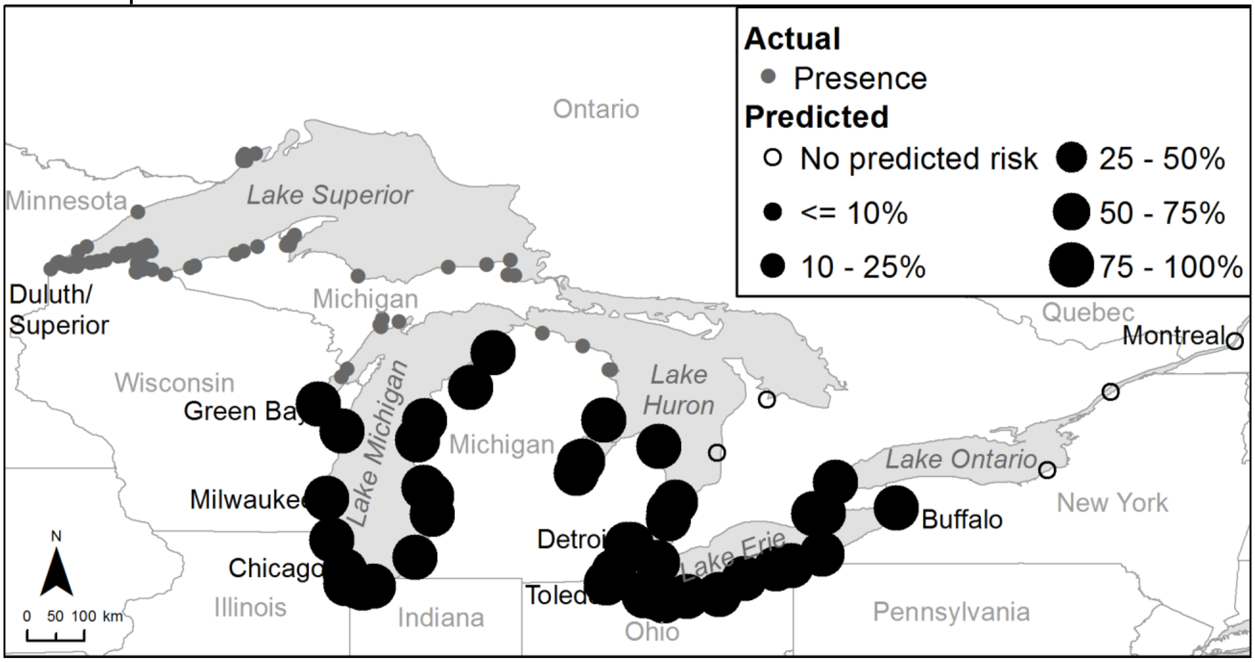

Time-step 7

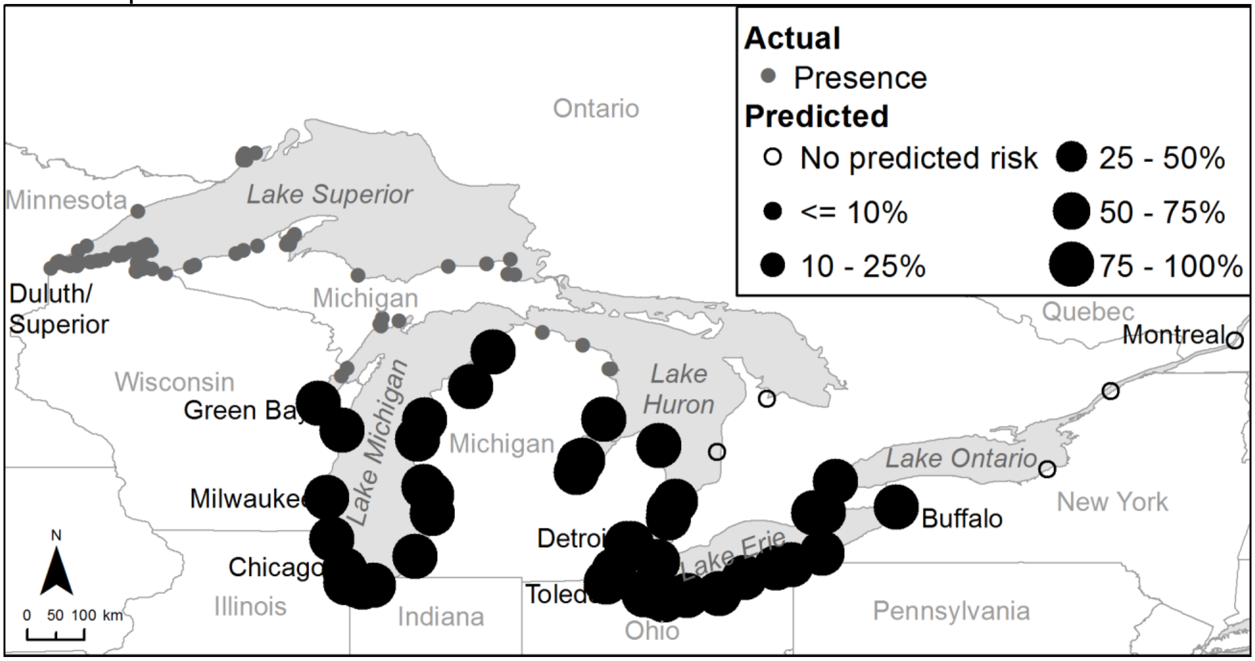

Time-step 8

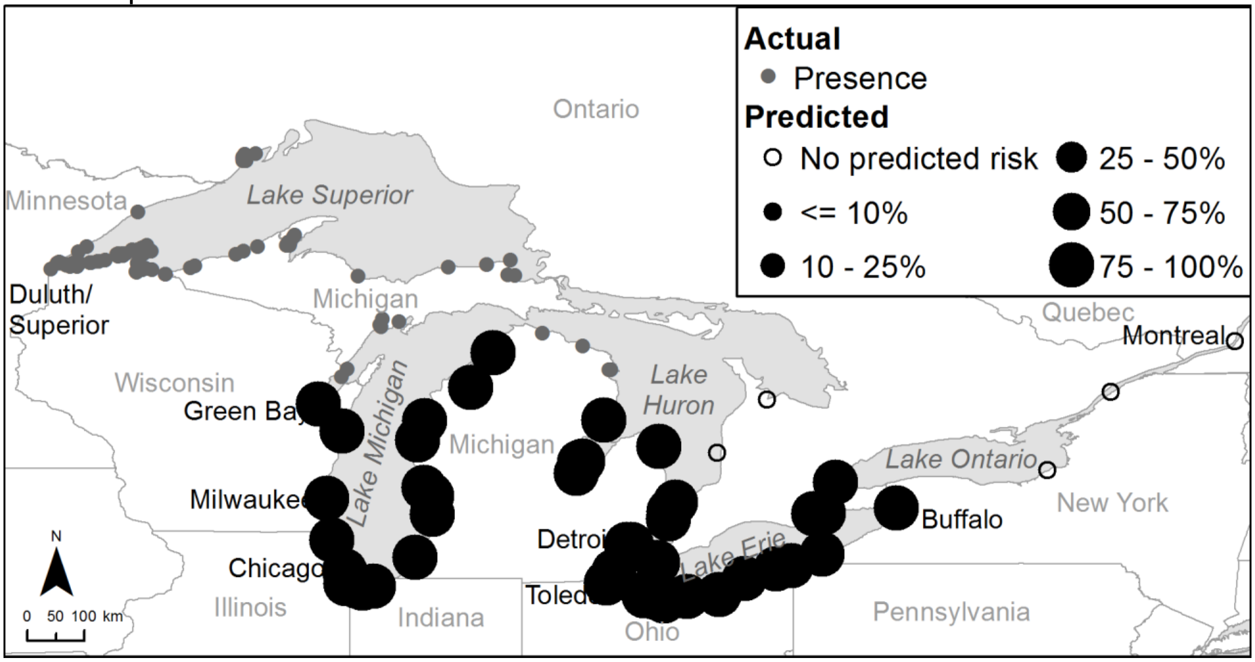

Time-step 9

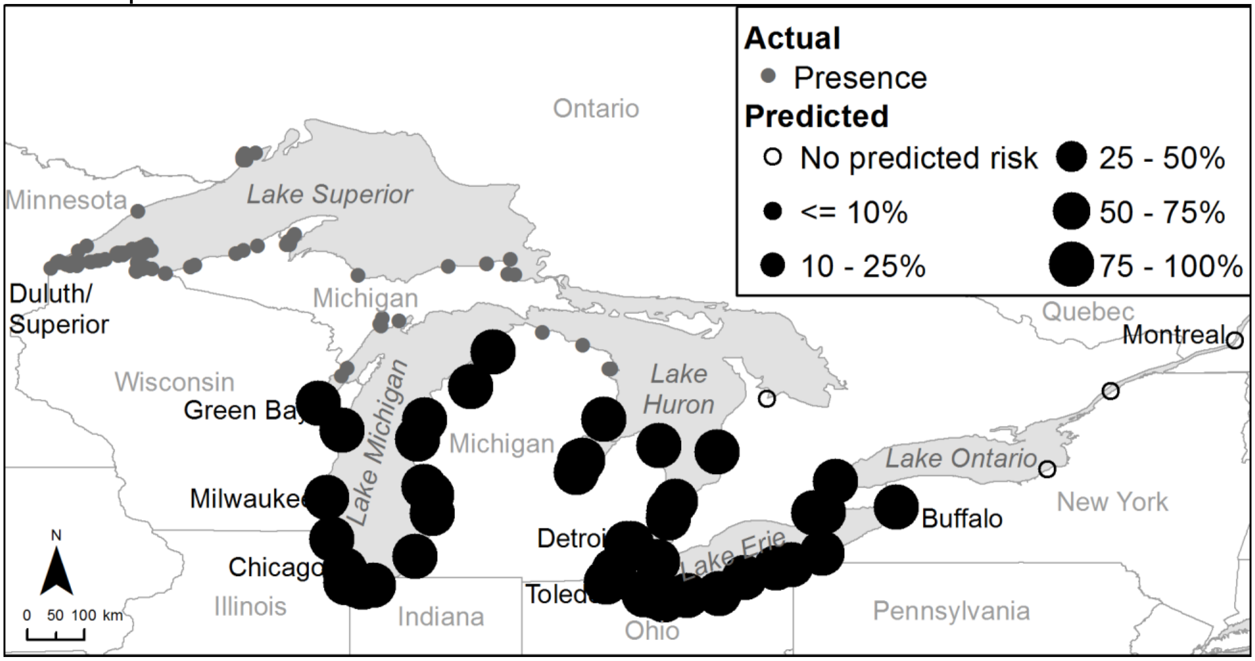

Time-step 10

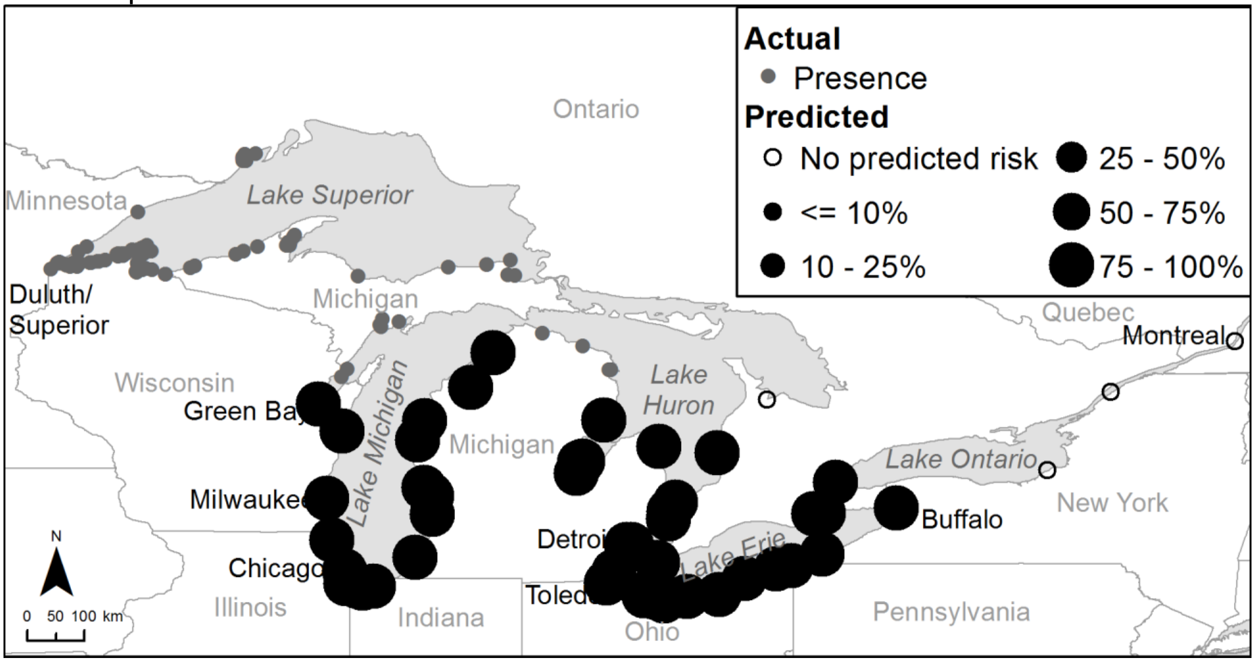

Killer Shrimp
Duluth, Minnesota, USA
Dispersal Distance = 0-km and Probability of Infestation = 0.75

Time-step 1

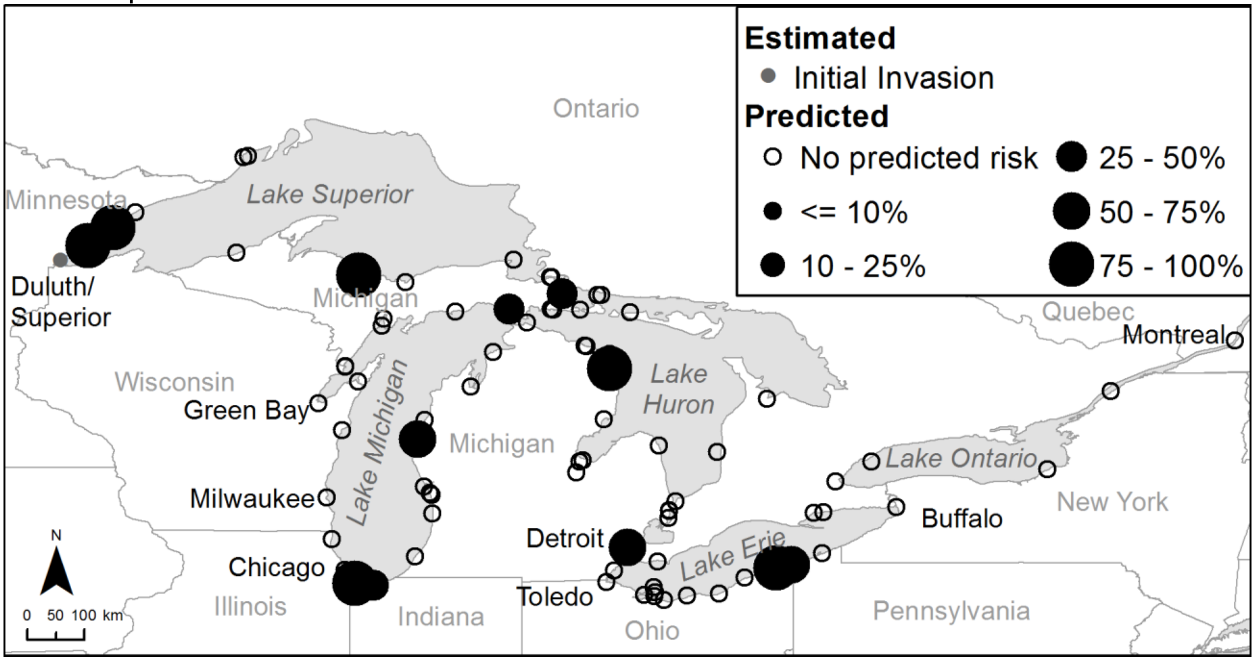

Time-step 2

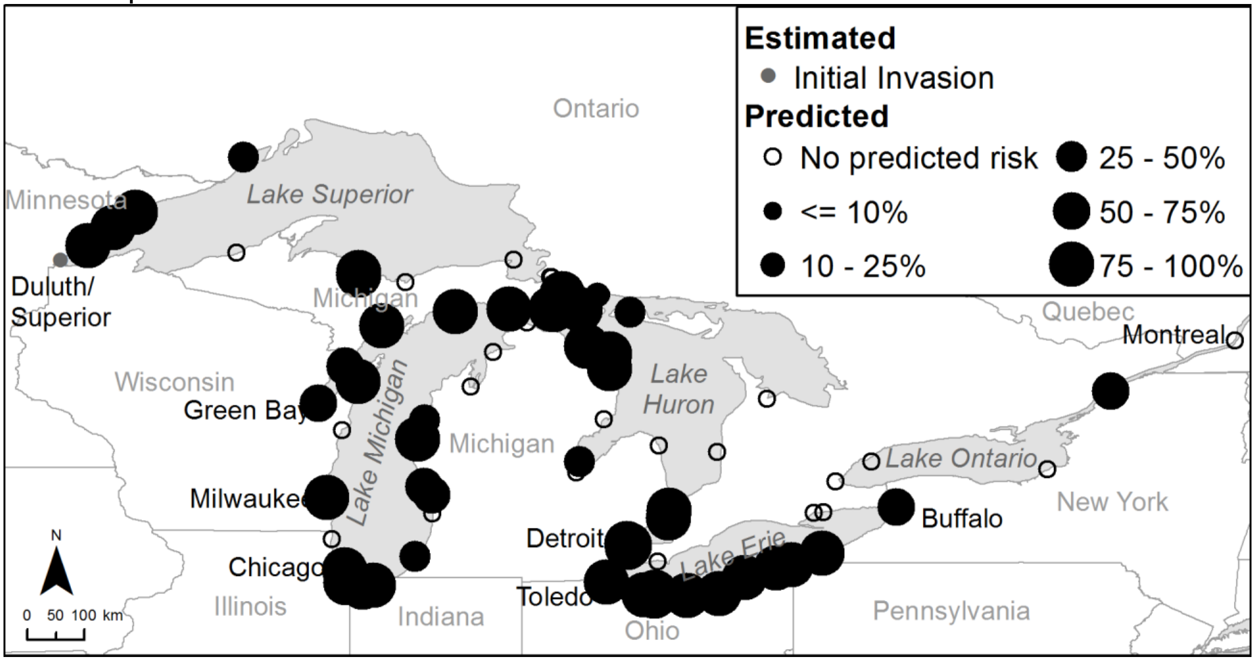

Time-step 3

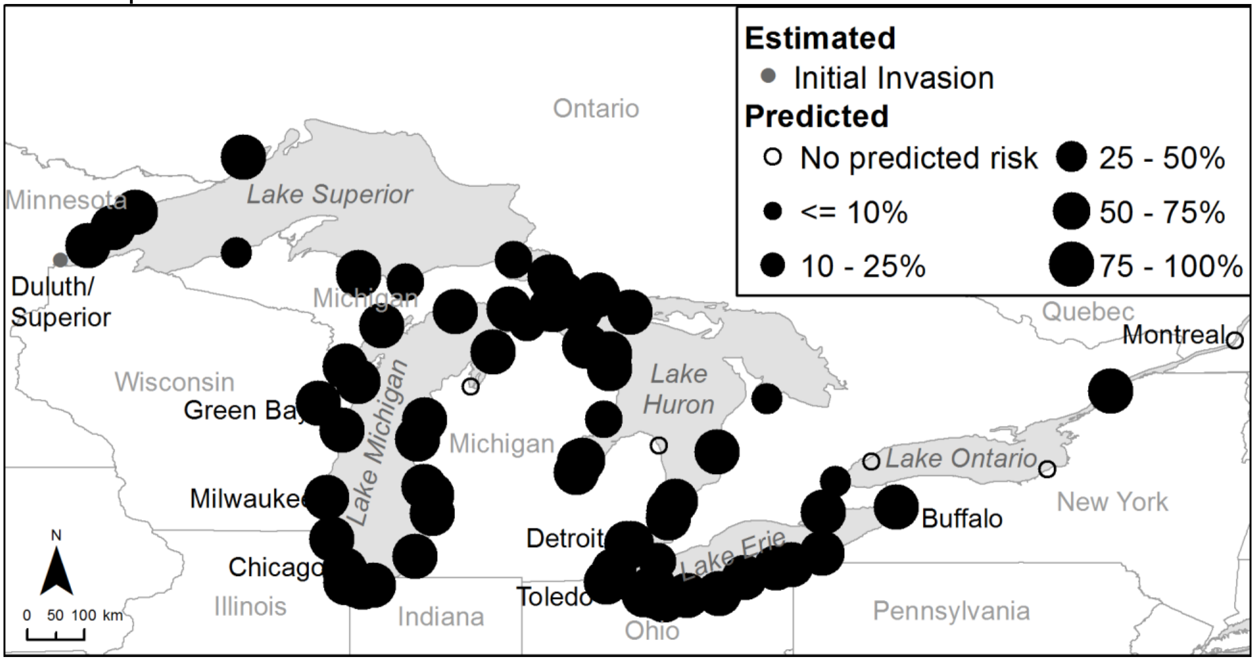

Time-step 4

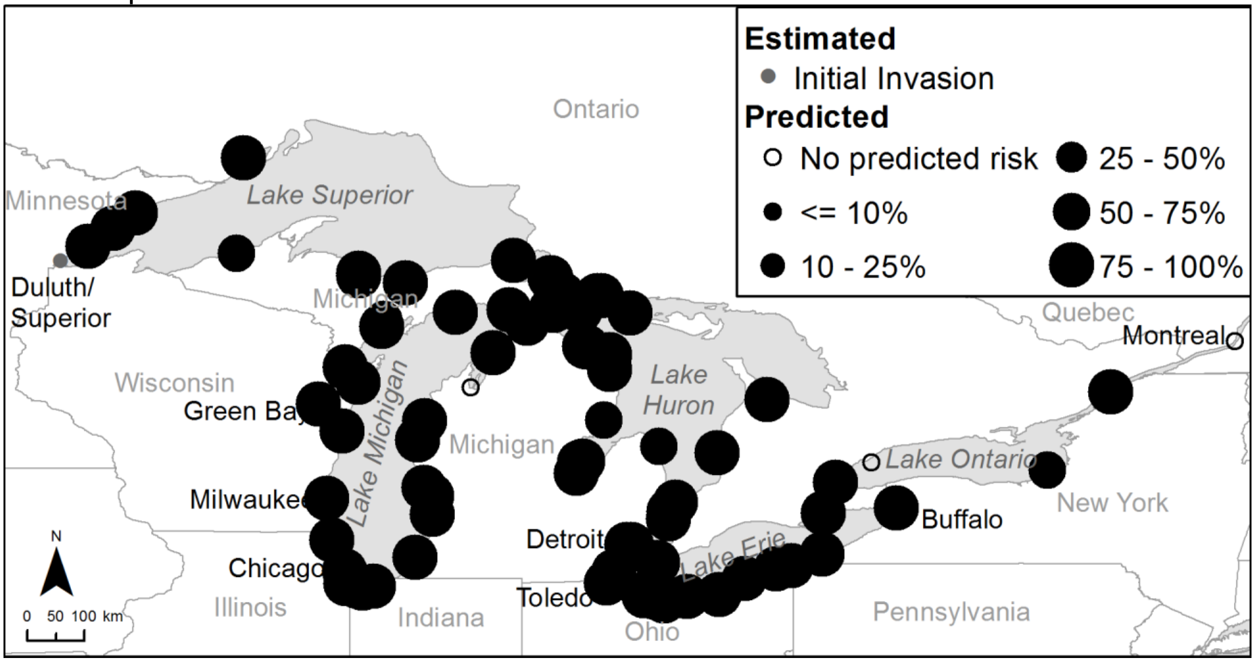

Time-step 5

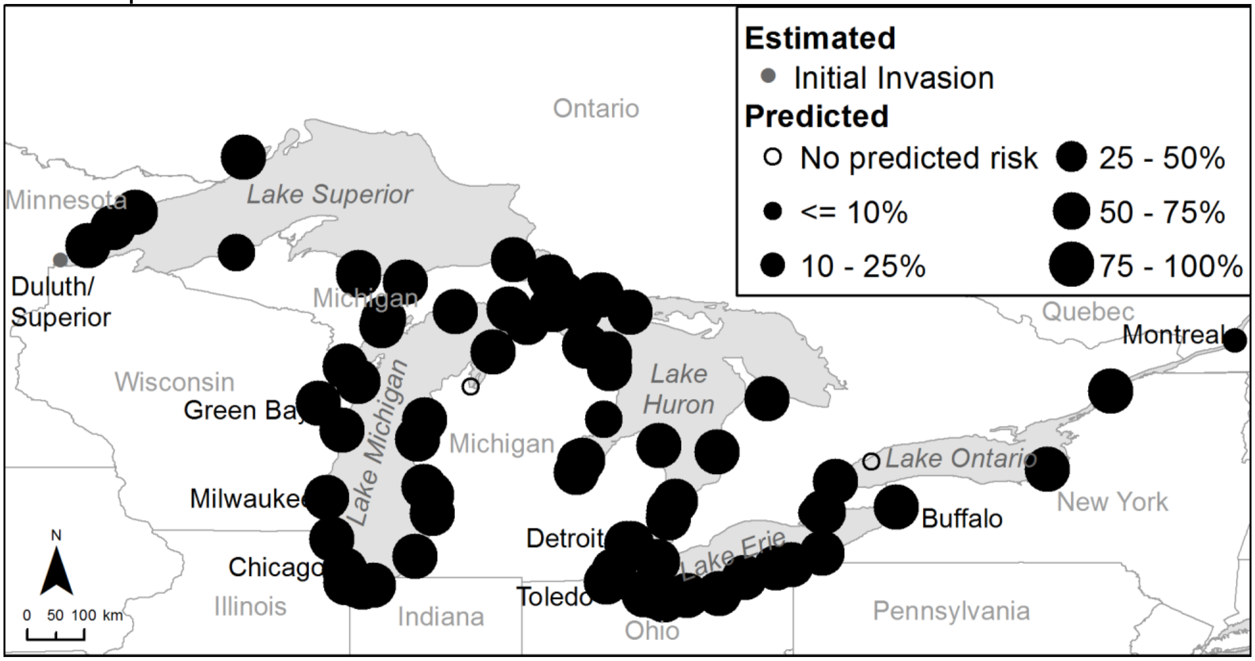

Time-step 6

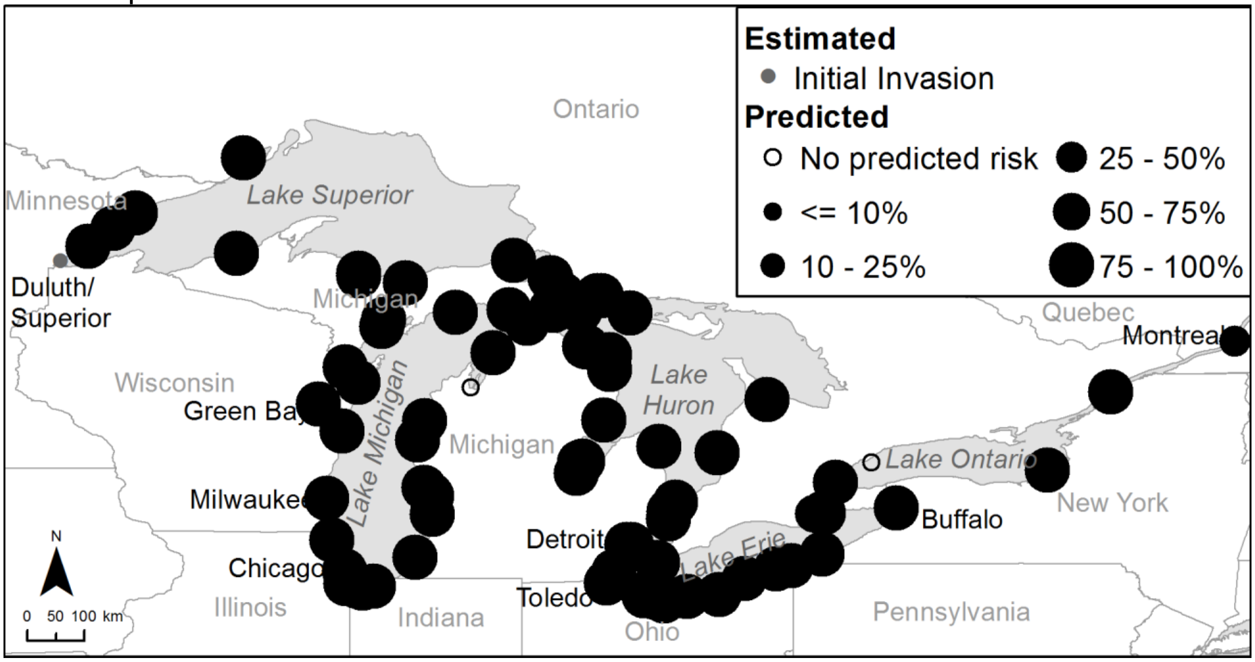

Time-step 7

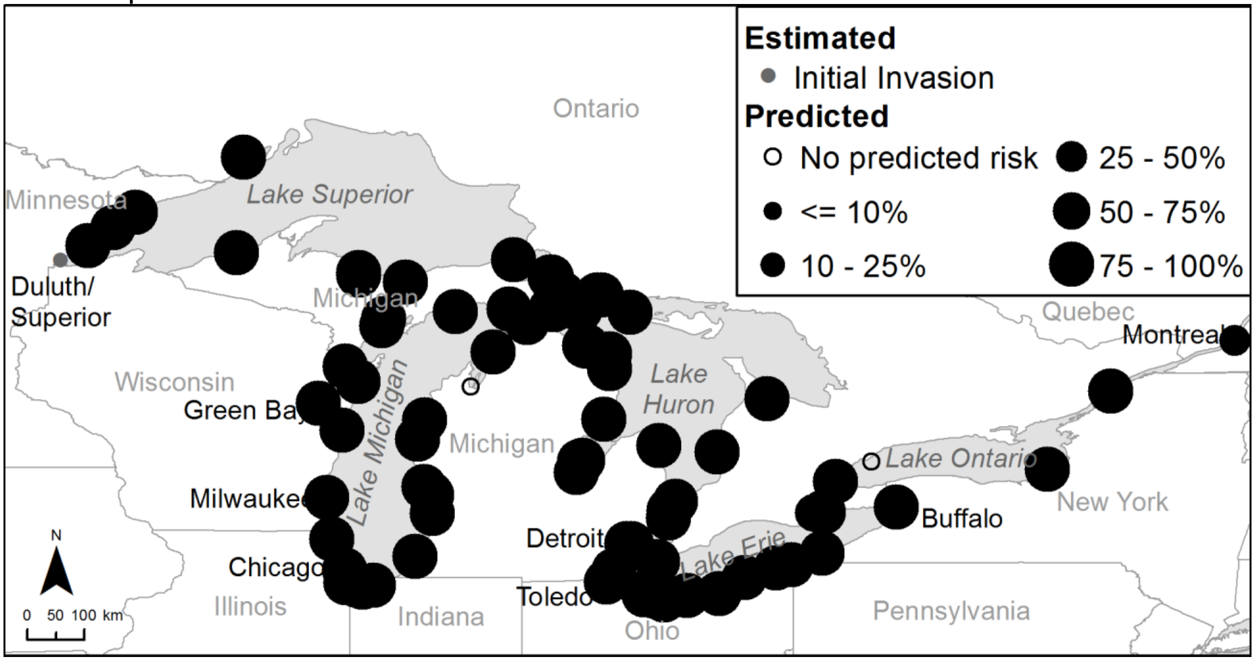

Time-step 8

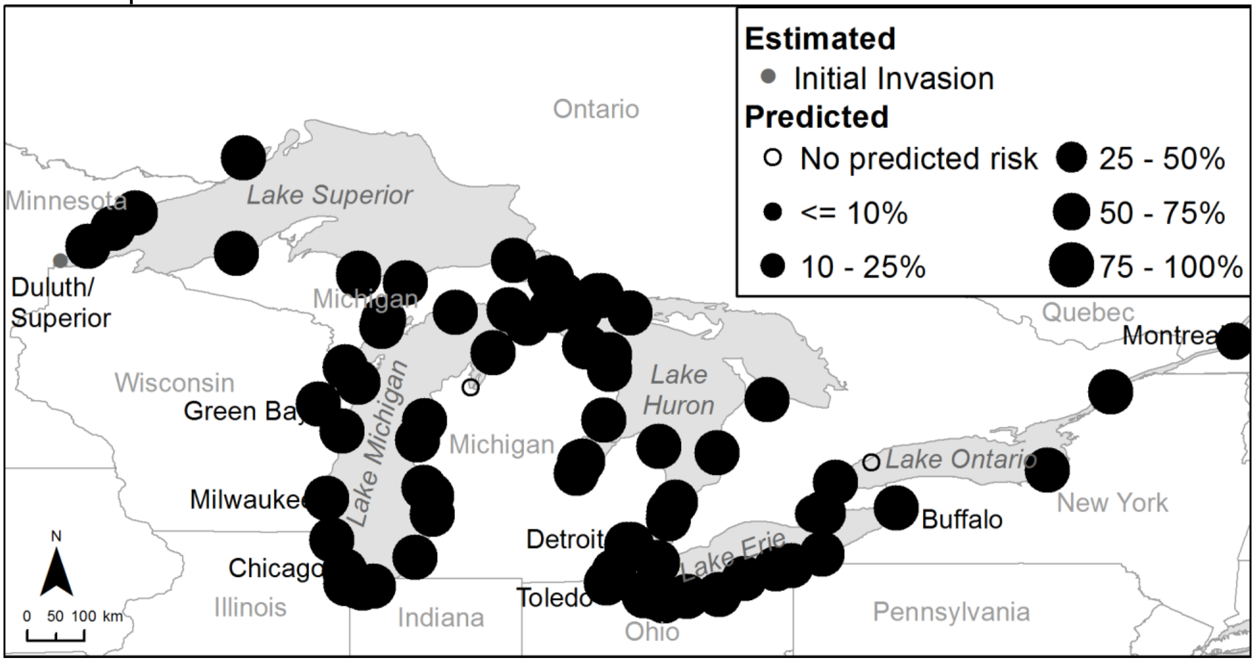

Time-step 9

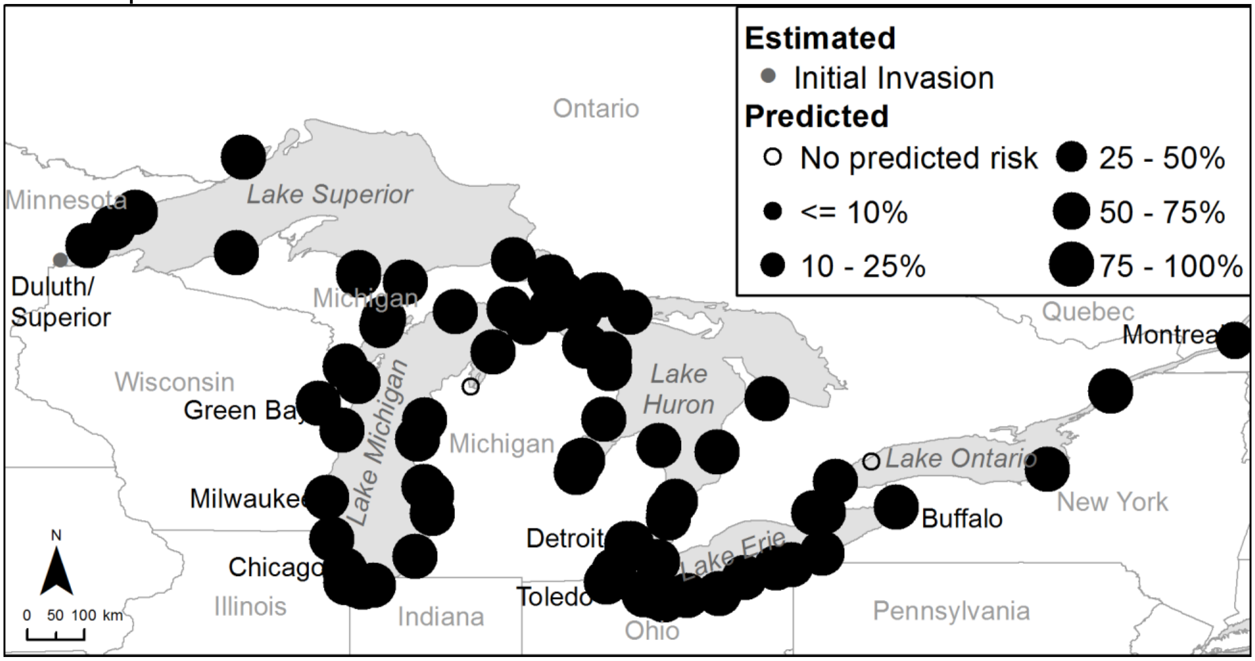

Time-step 10

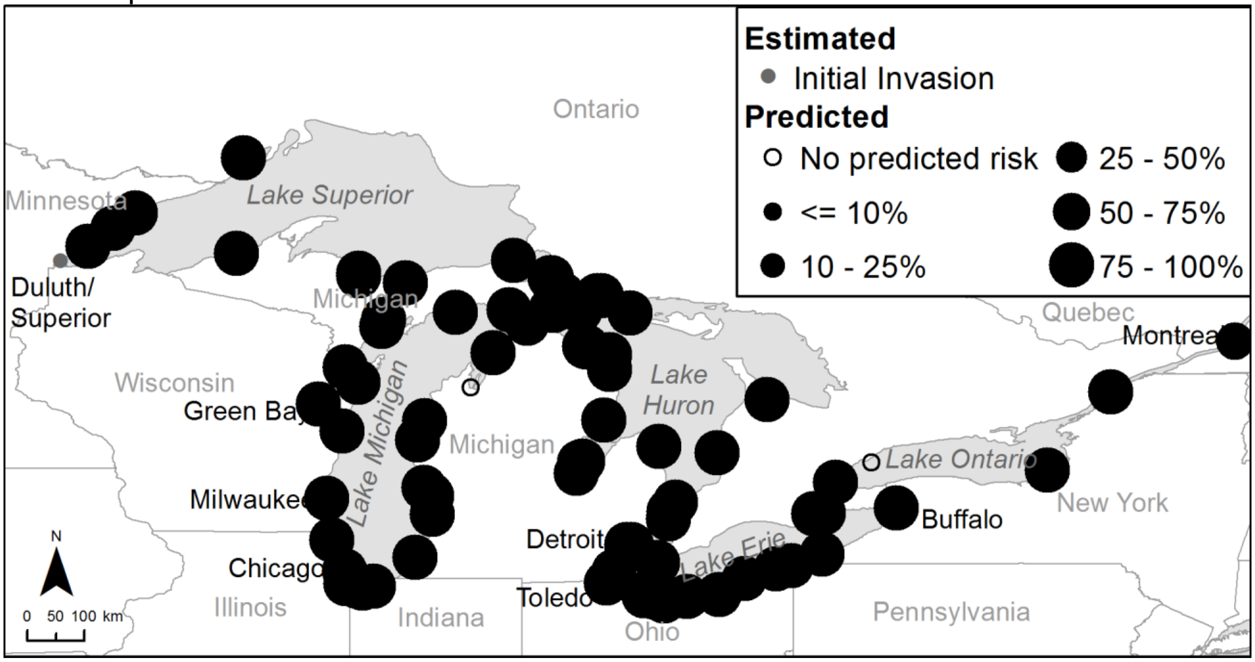

Killer Shrimp
Toledo, Ohio, USA
Dispersal Distance = 0-km and Probability of Infestation = 0.75

Time-step 1

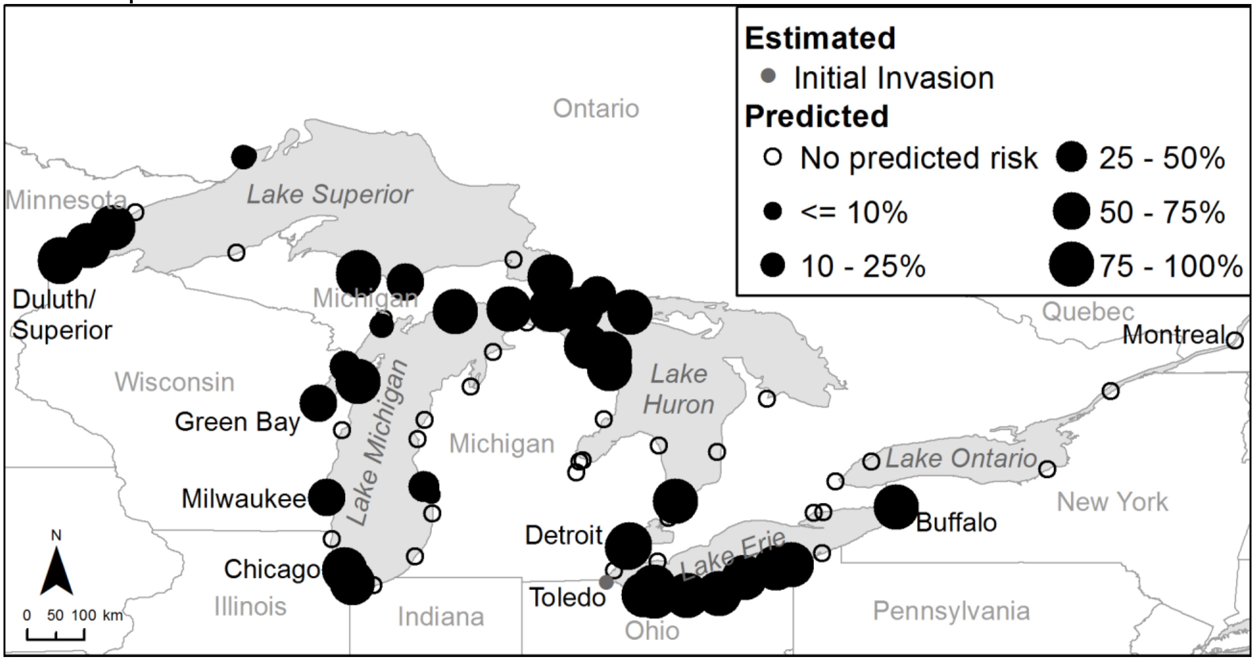

Time-step 2

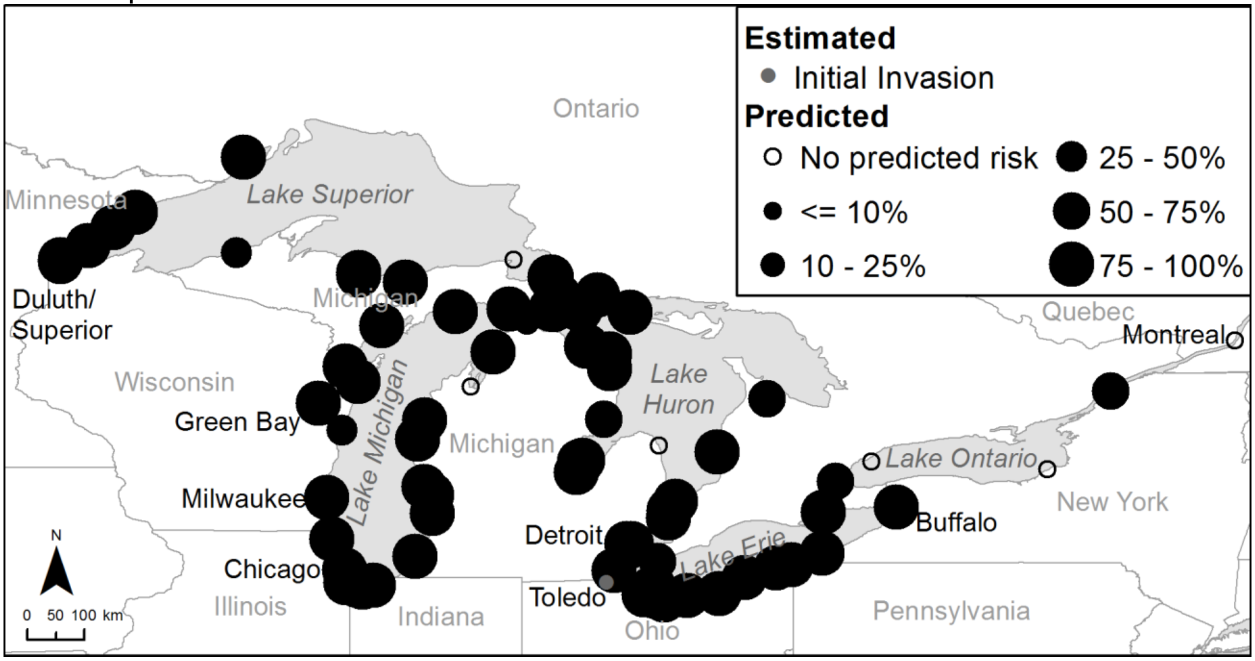

Time-step 3

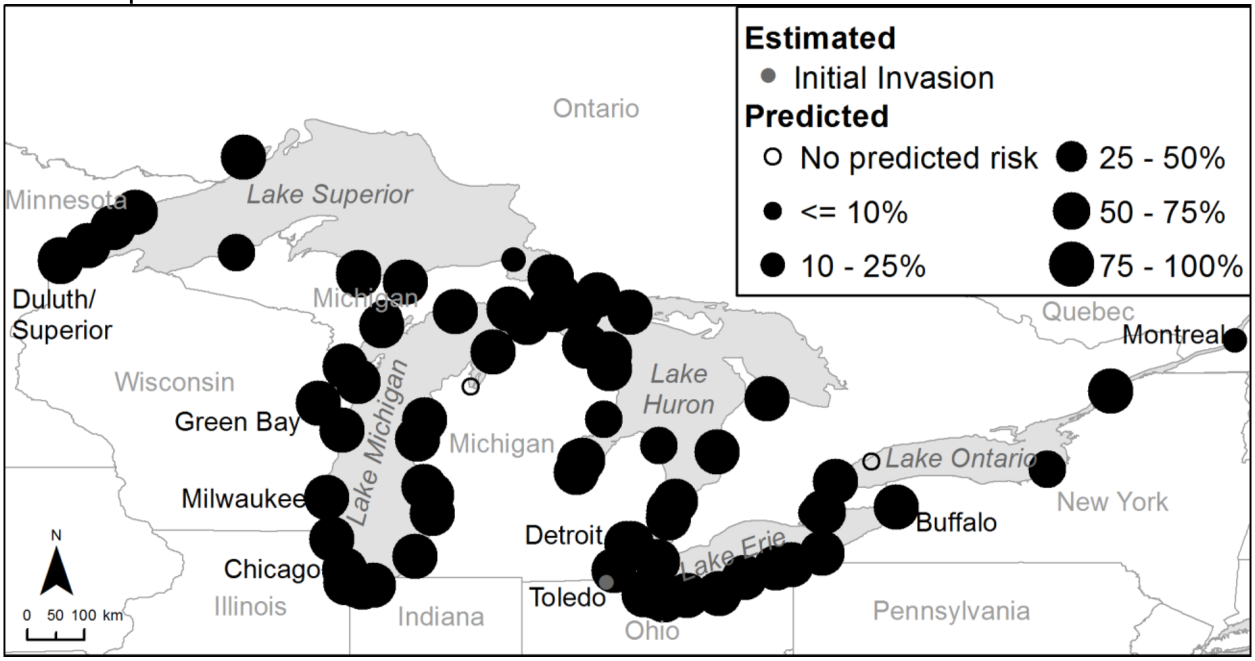

Time-step 4

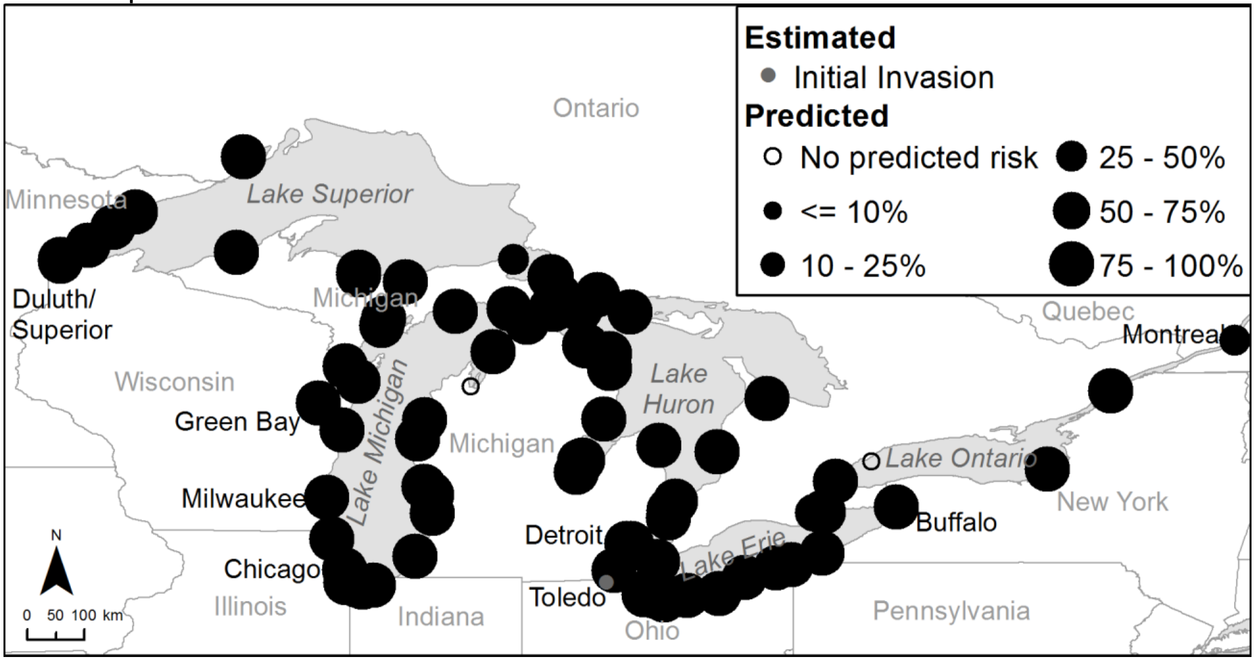

Time-step 5

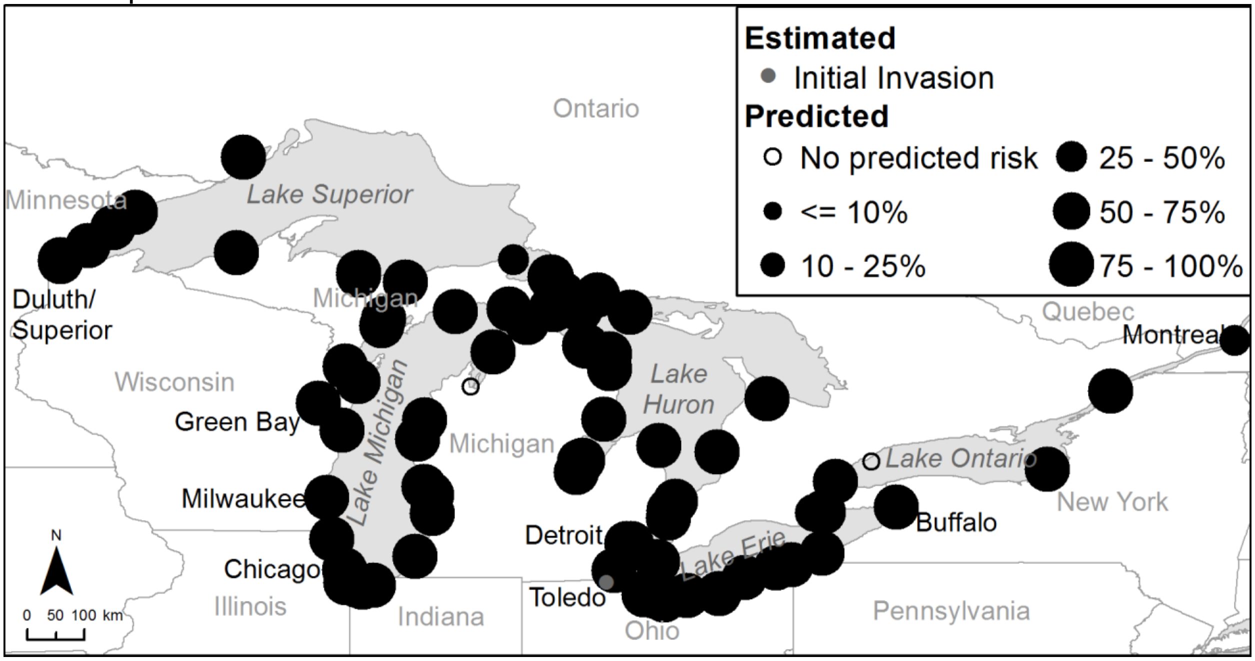

Time-step 6

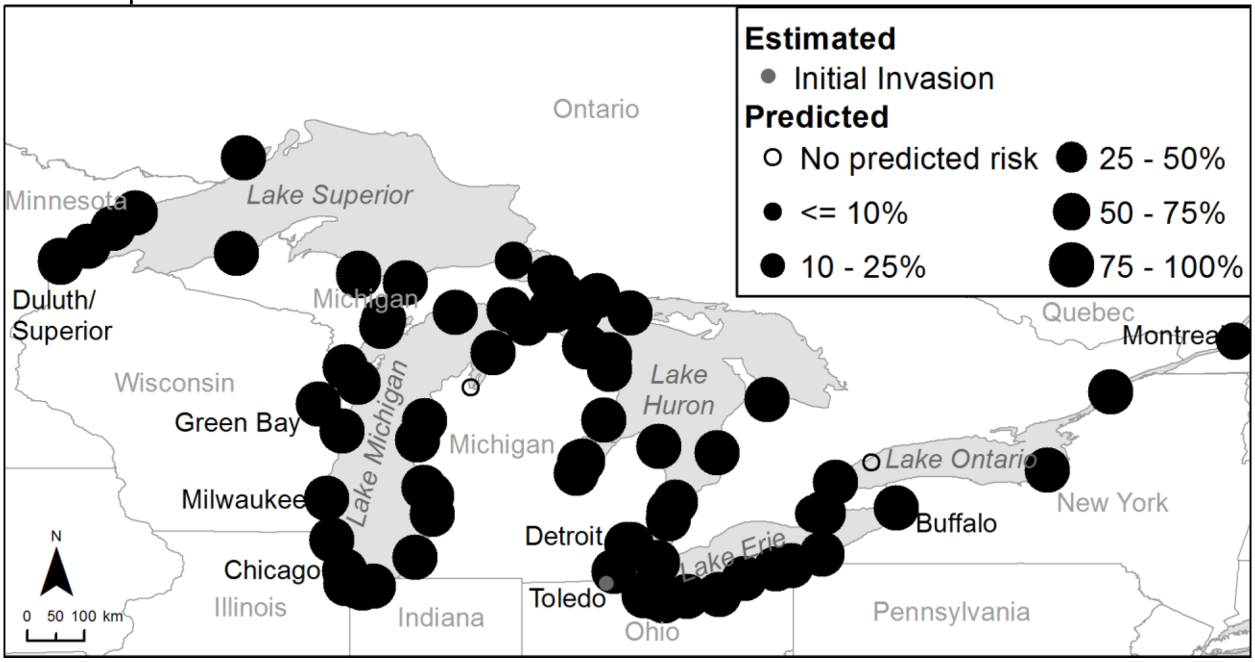

Time-step 7

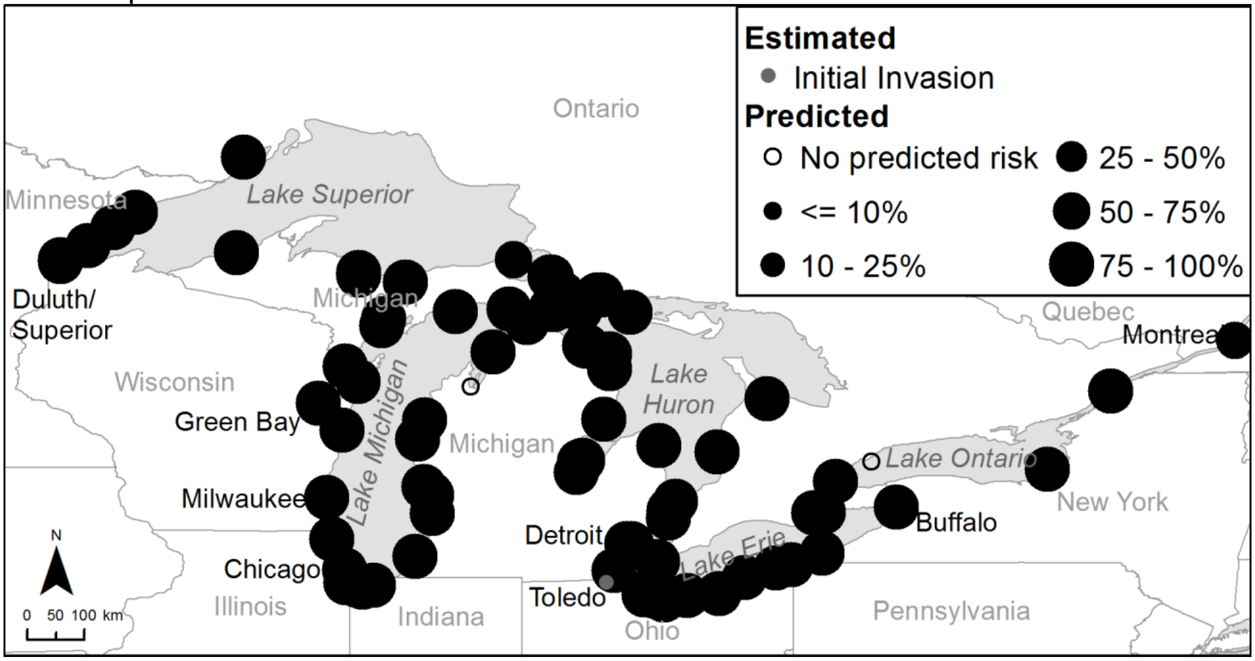

Time-step 8

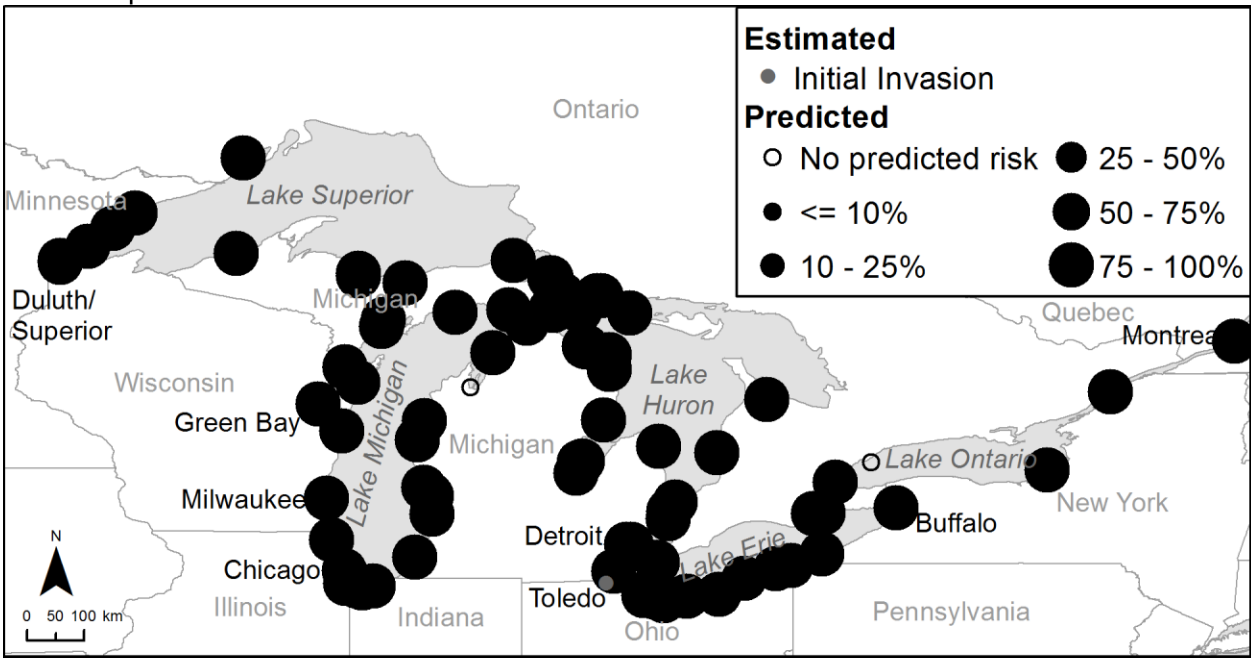

Time-step 9

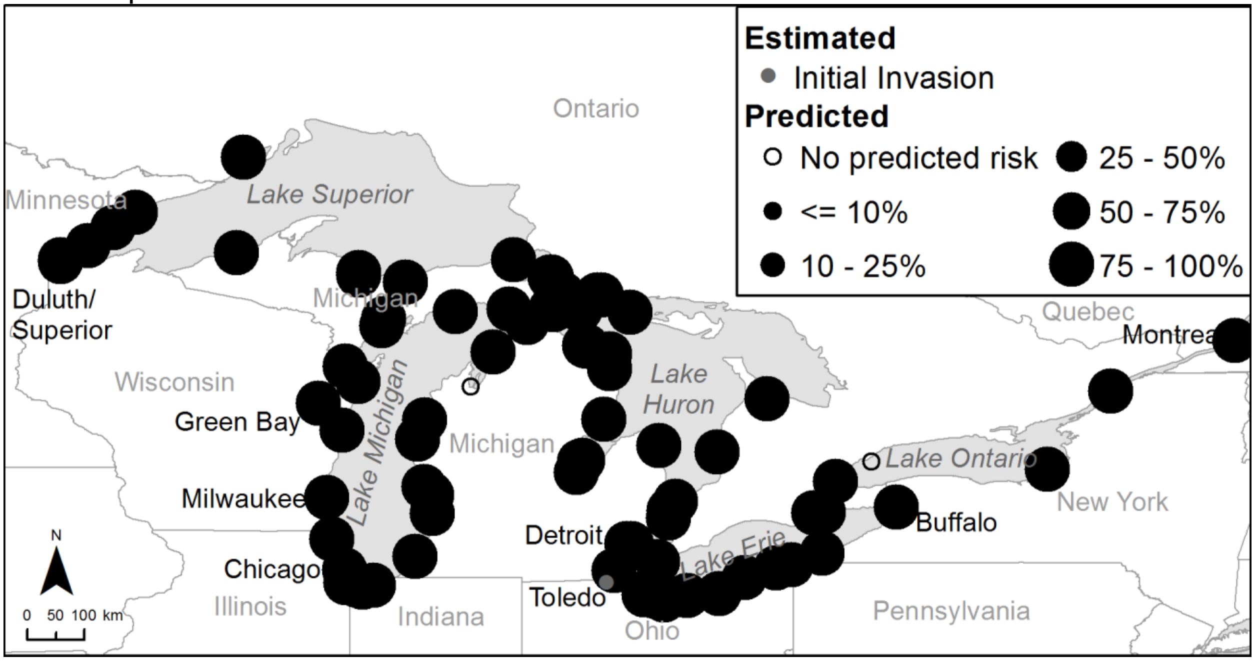

Time-step 10

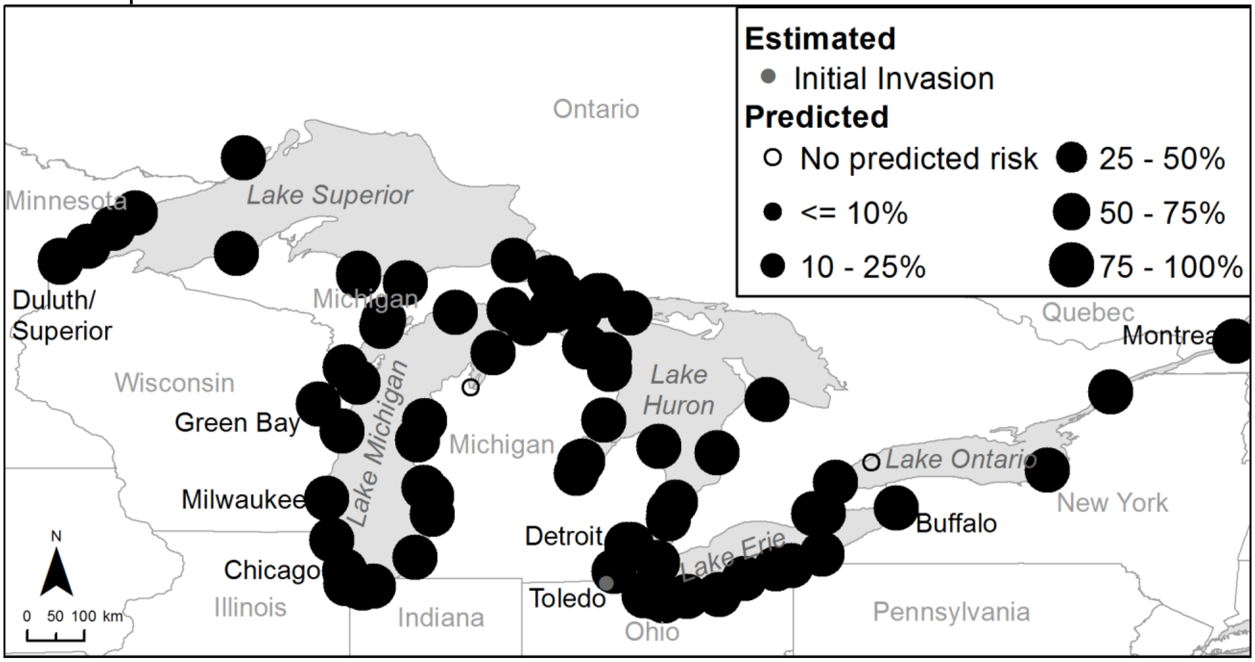

Killer Shrimp
Ogdensburg, New York, USA
Dispersal Distance = 0-km and Probability of Infestation = 0.75

Time-step 1

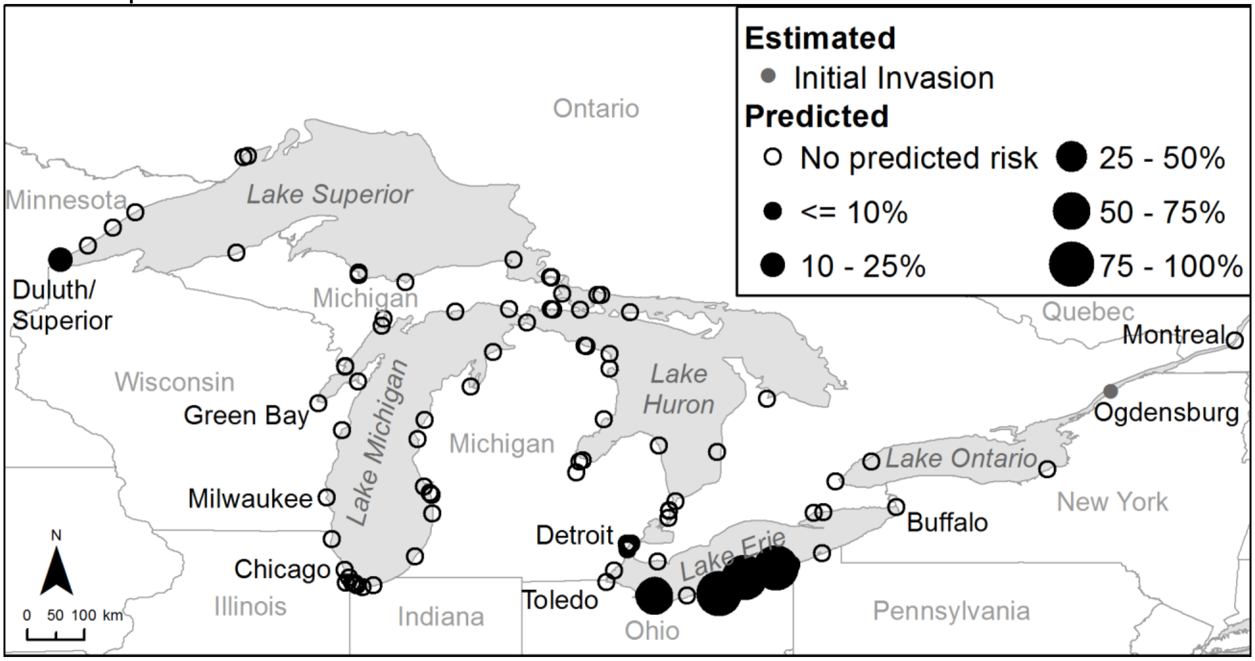

Time-step 2

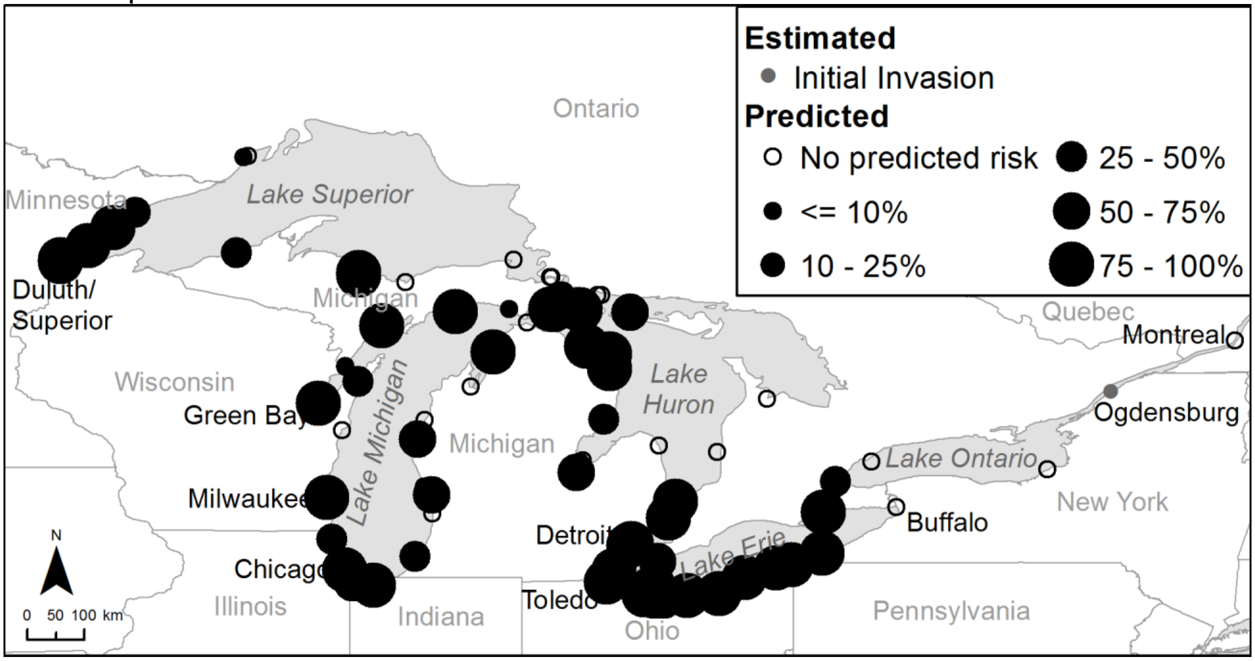

Time-step 3

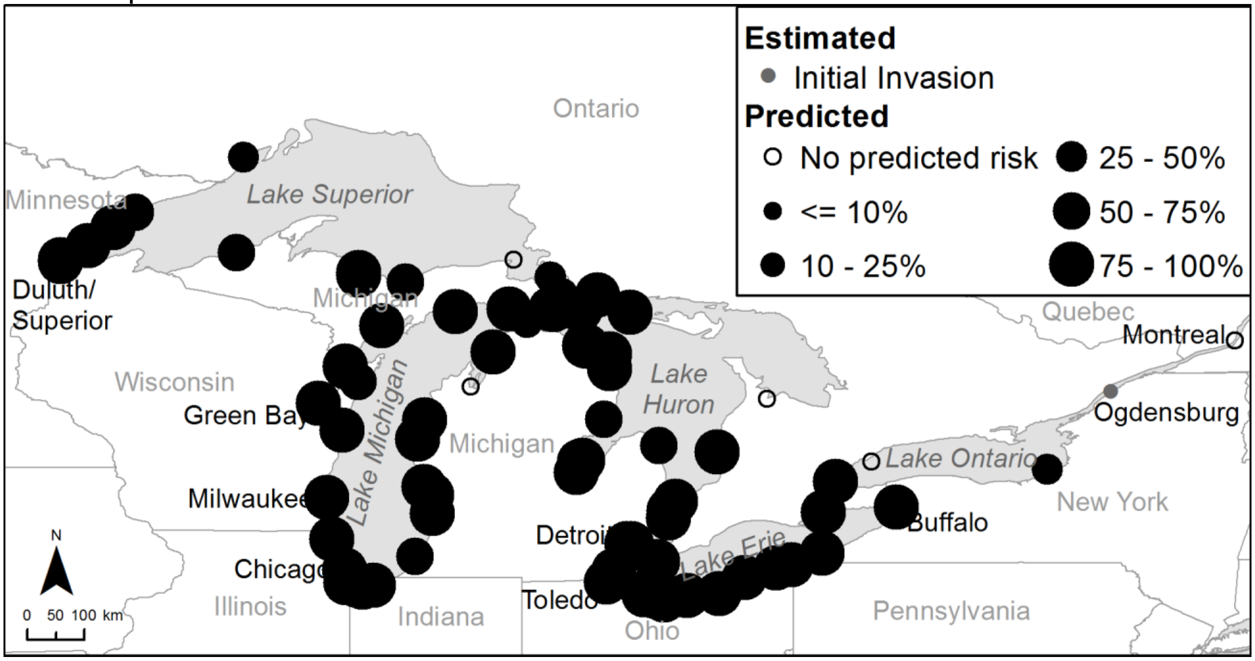

Time-step 4

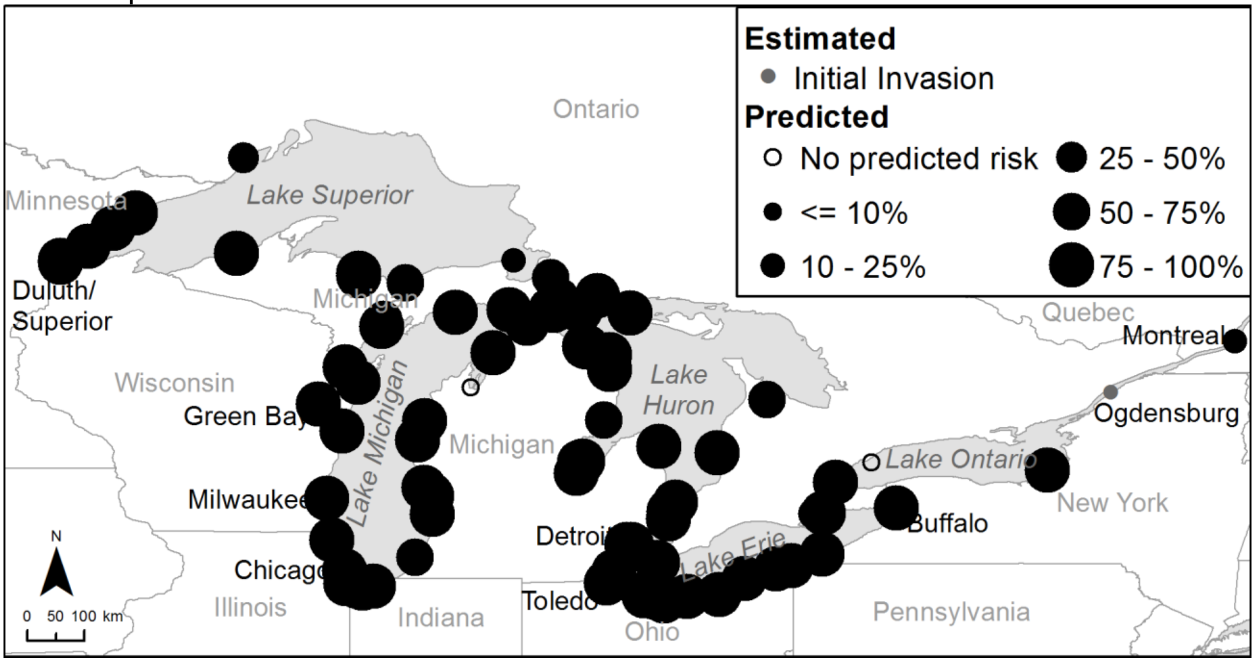

Time-step 5

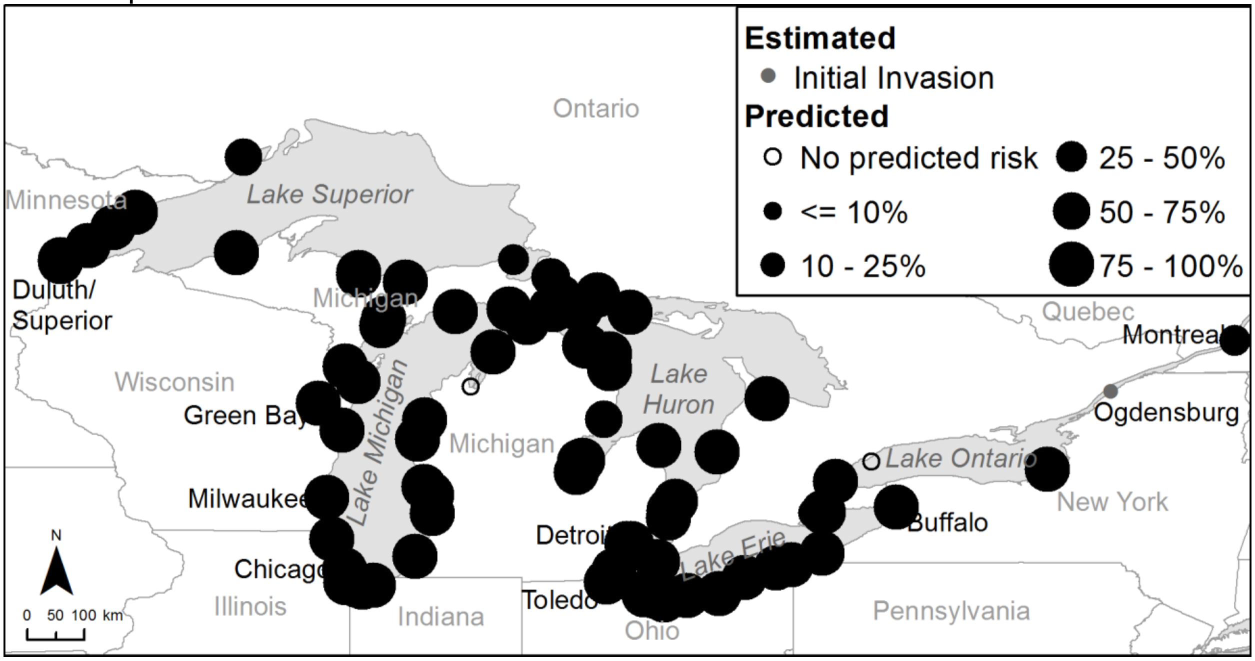

Time-step 6

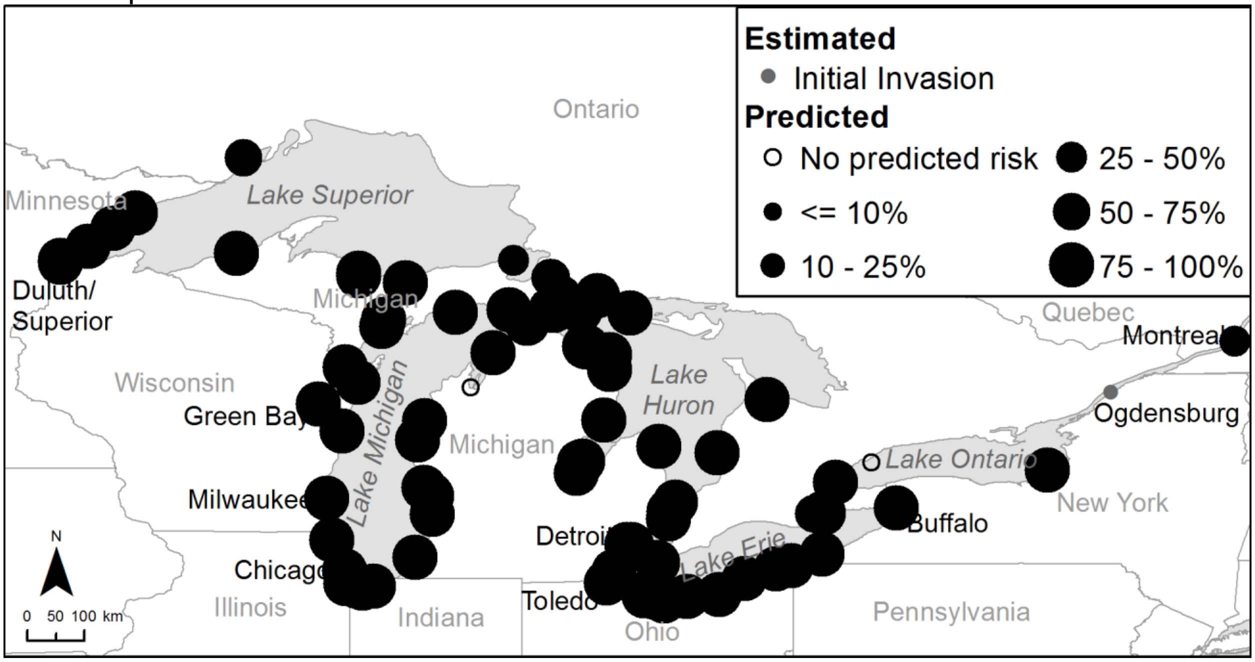

Time-step 7

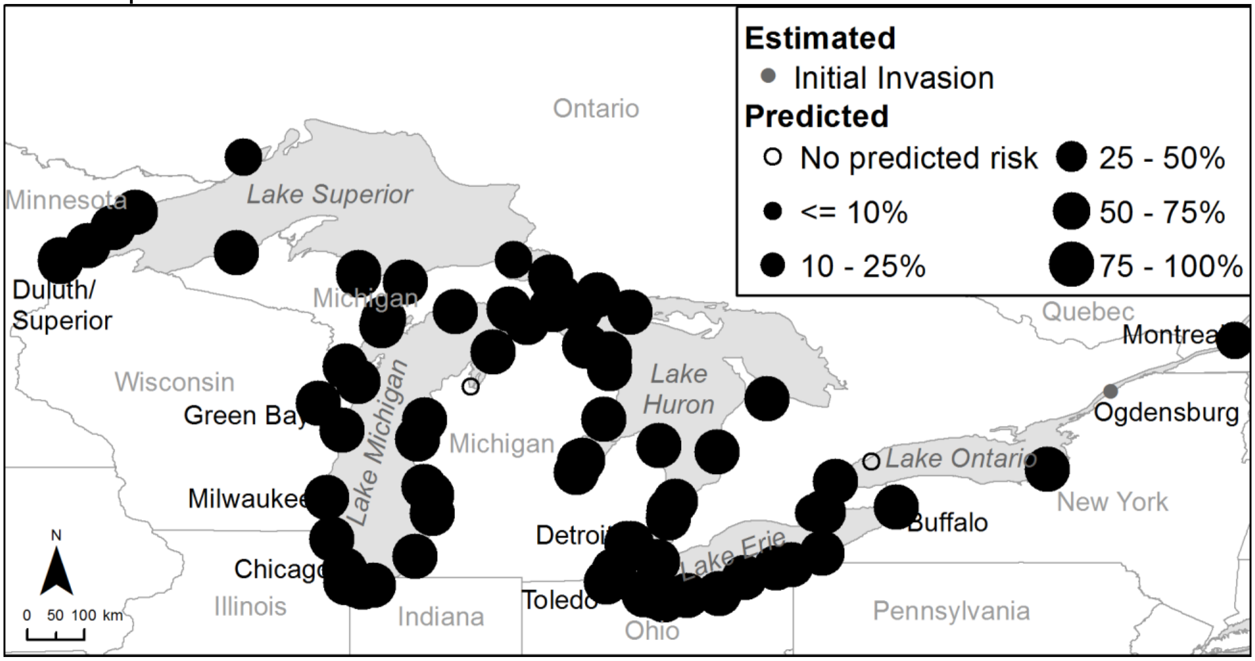

Time-step 8

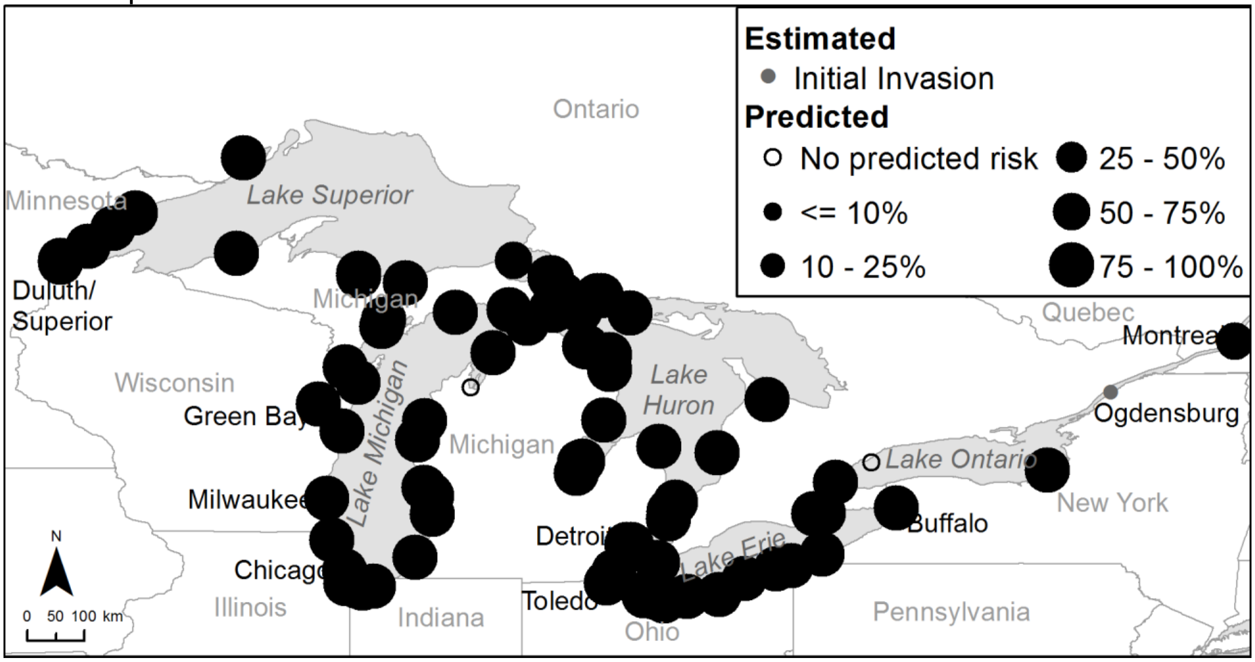

Time-step 9

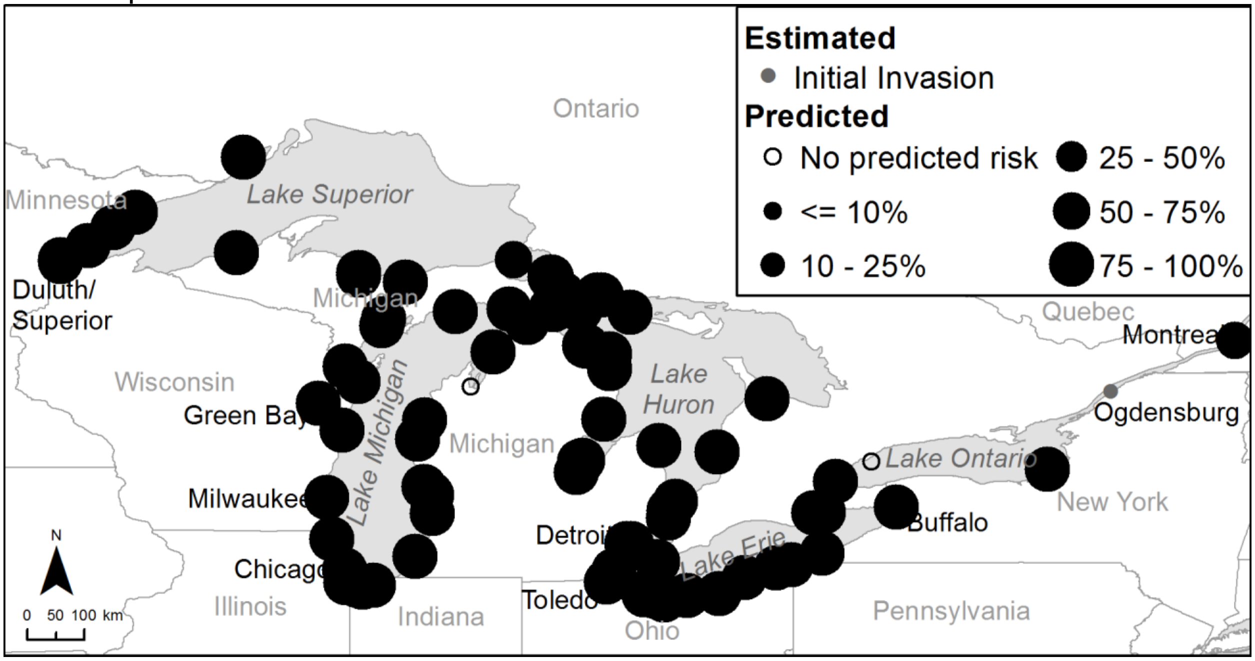

Time-step 10

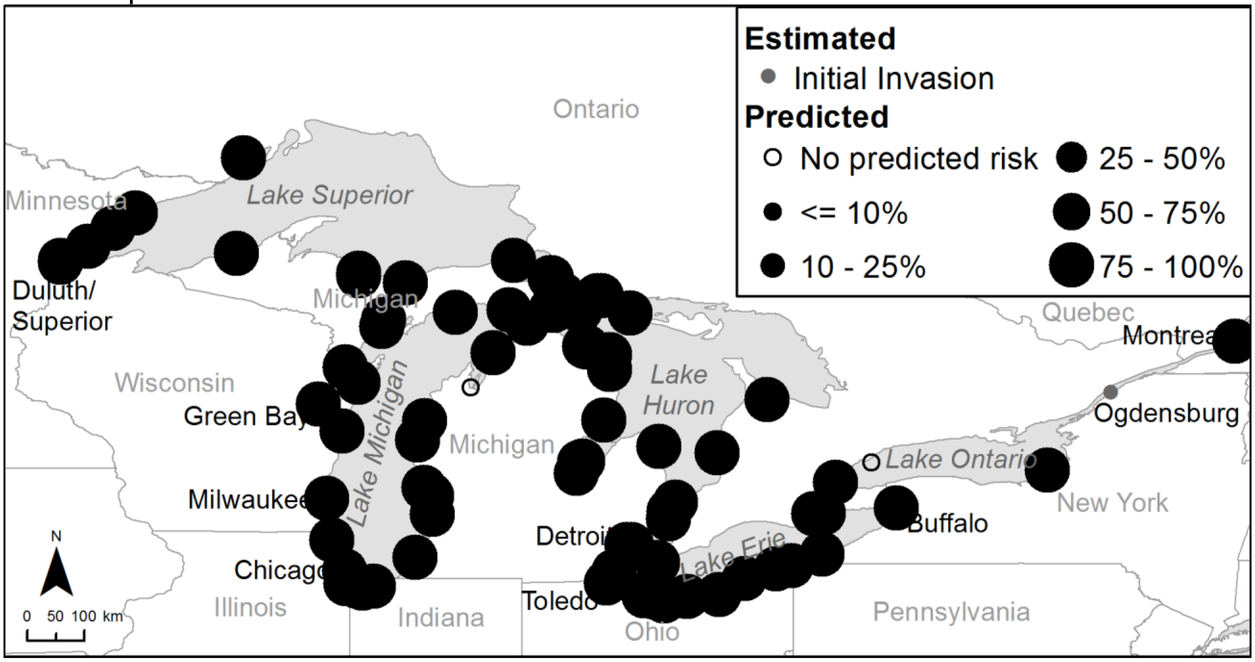

Killer Shrimp
Green Bay, Wisconsin, USA
Dispersal Distance = 0-km and Probability of Infestation = 0.75

Time-step 1

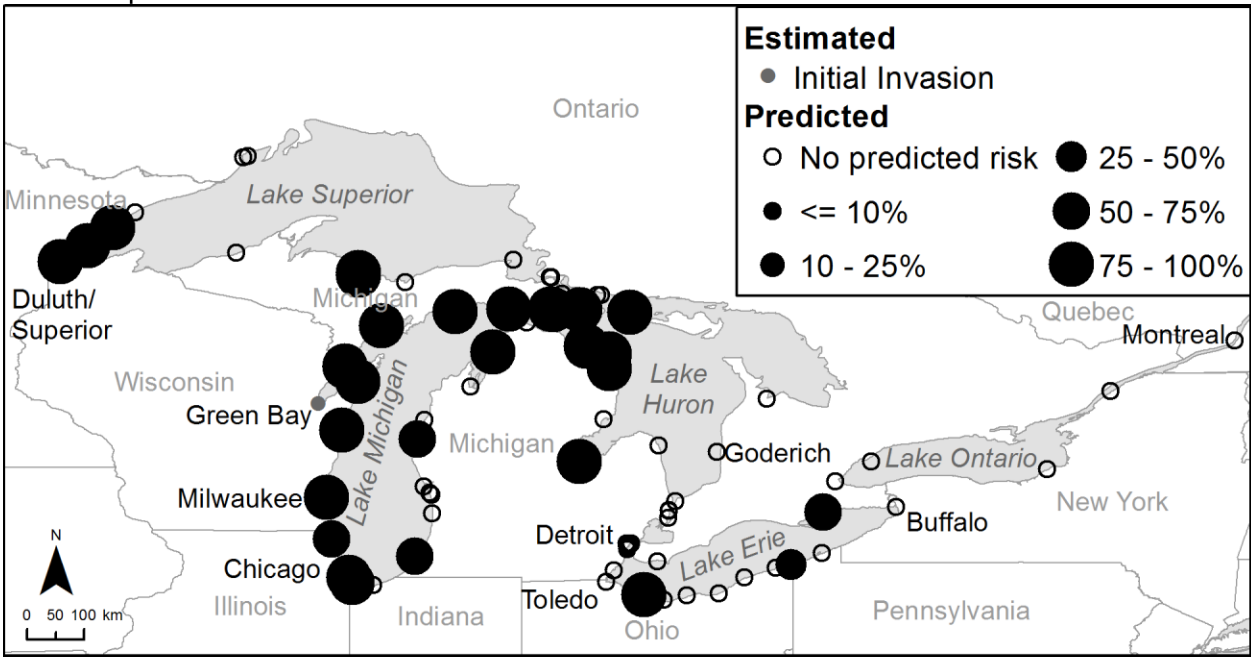

Time-step 2

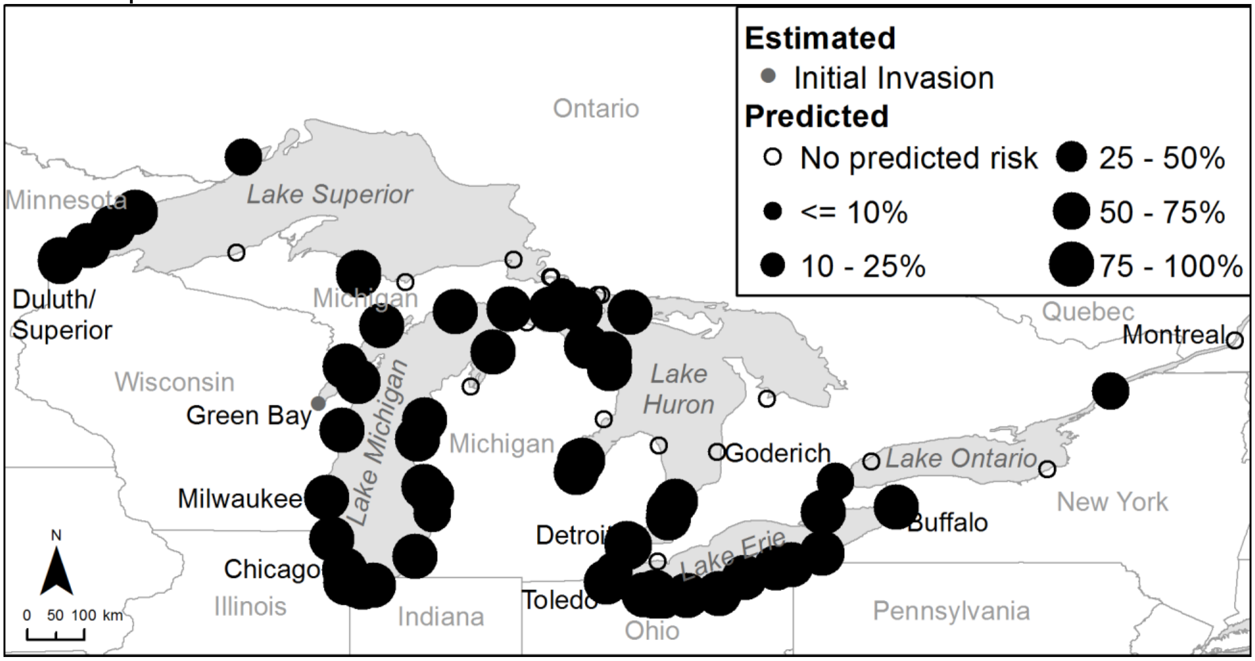

Time-step 3

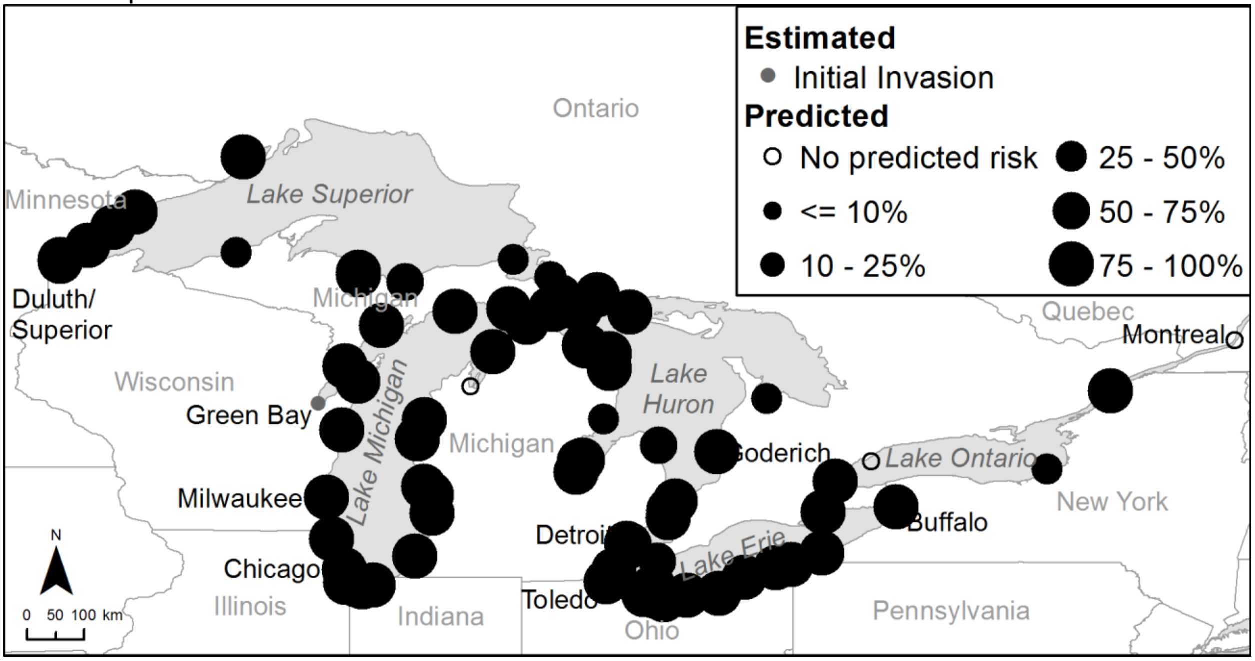

Time-step 4

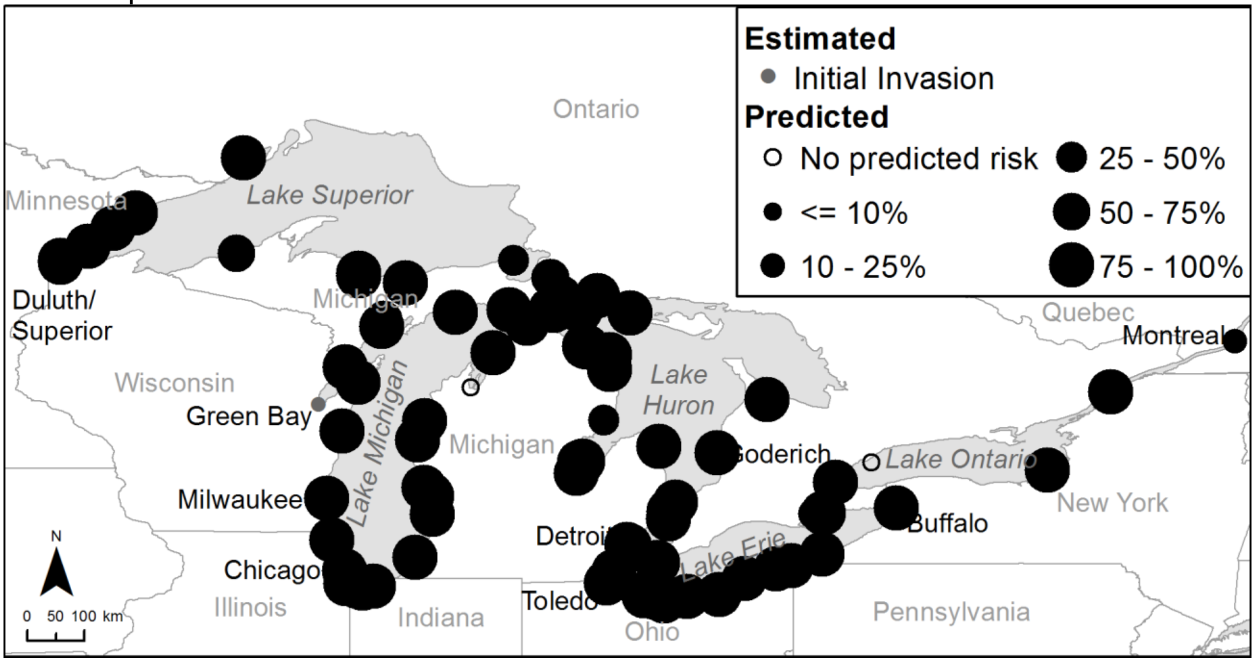

Time-step 5

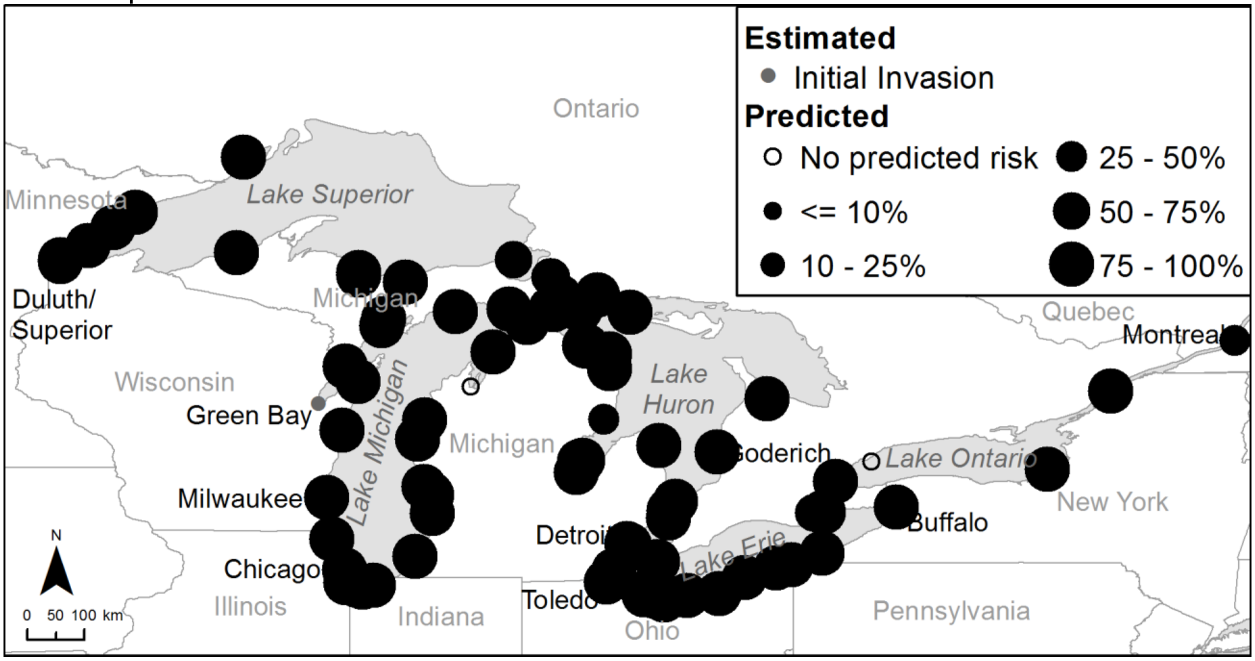

Time-step 6

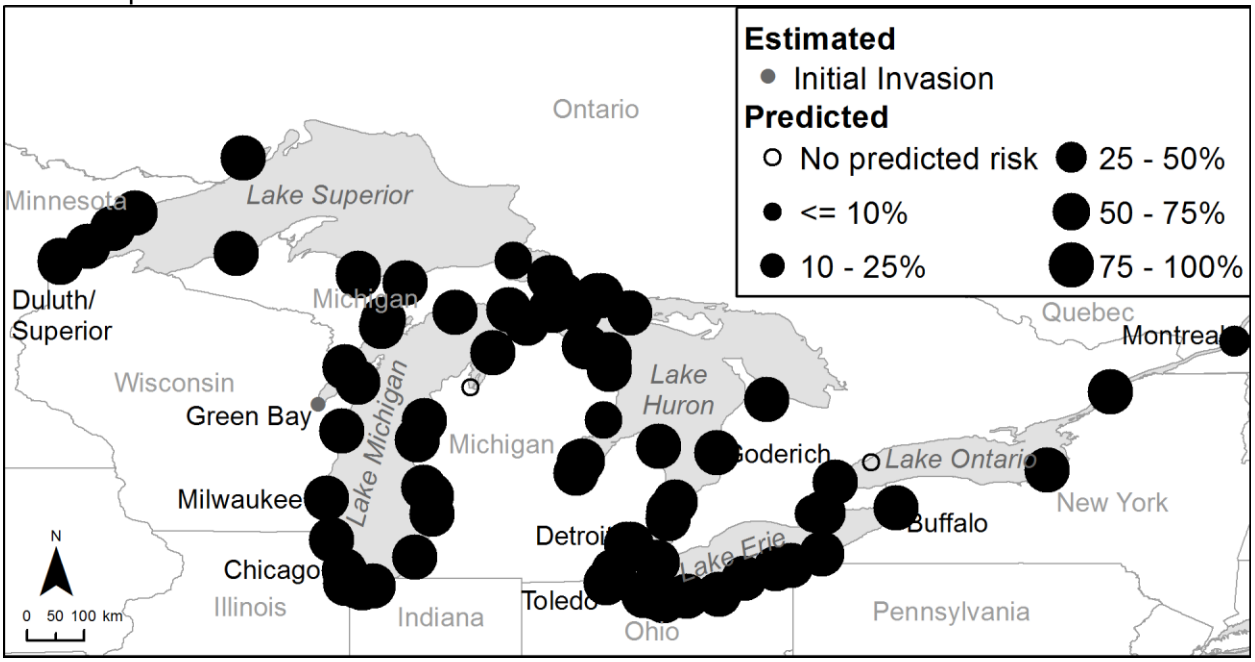

Time-step 7

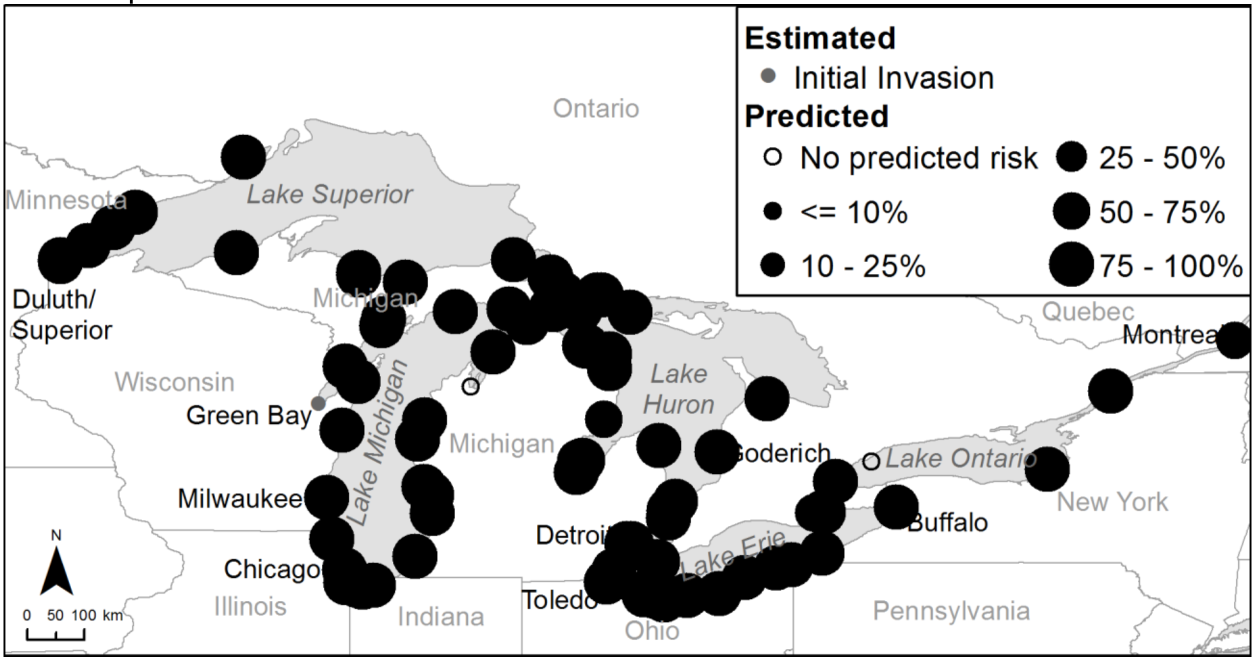

Time-step 8

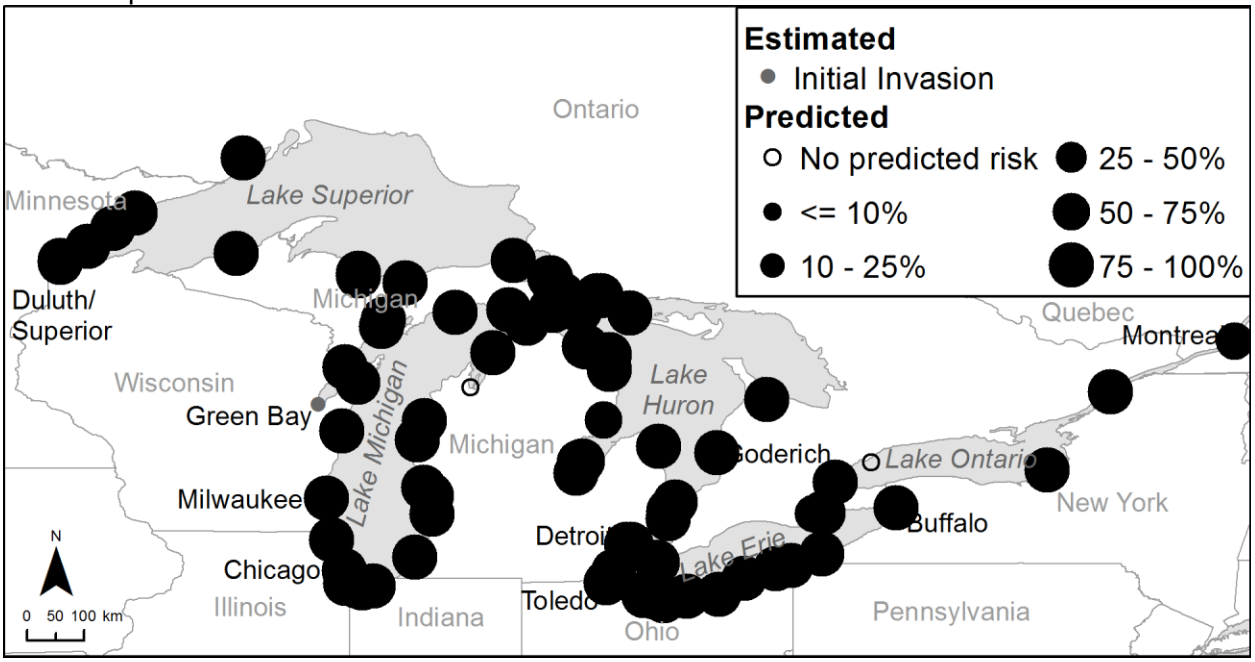

Time-step 9

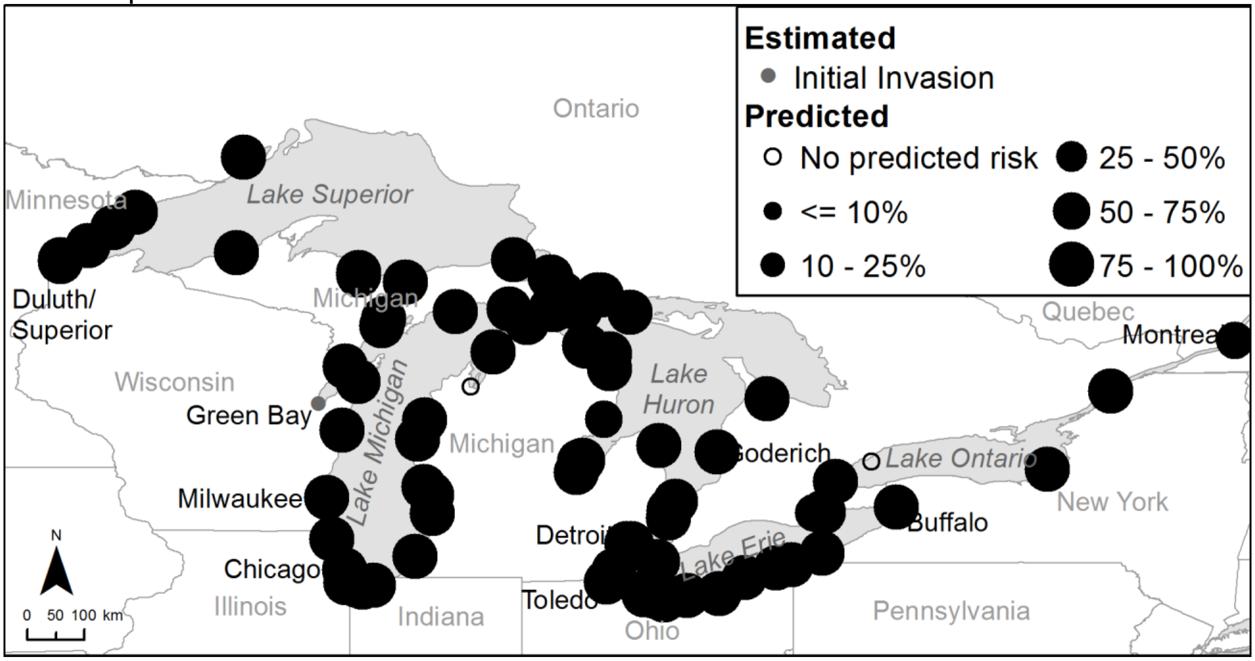

Time-step 10

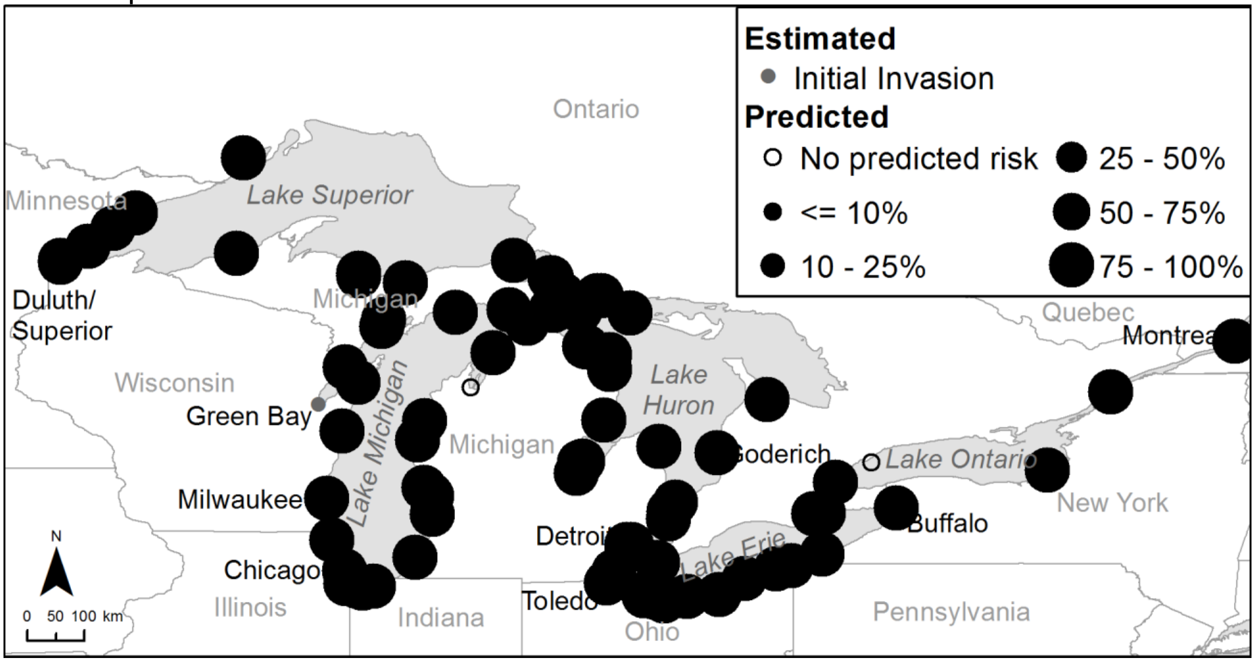

Killer Shrimp
Goderich, Ontario, Canada
Dispersal Distance = 0-km and Probability of Infestation = 0.75

Time-step 1

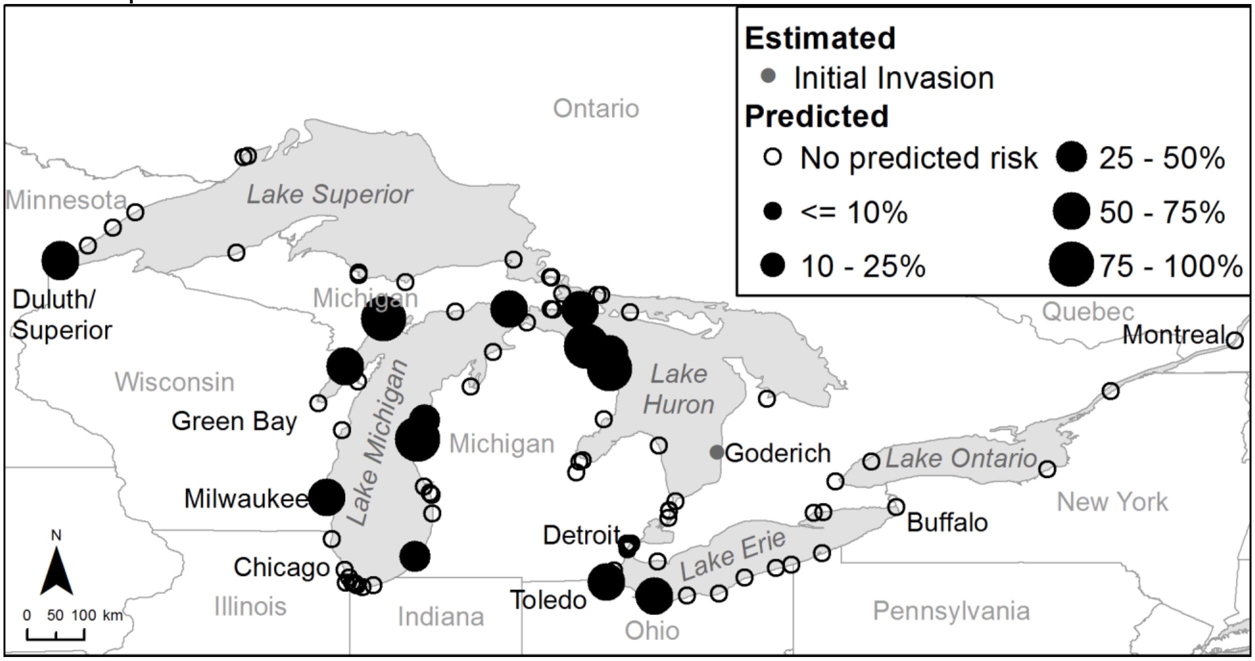

Time-step 2

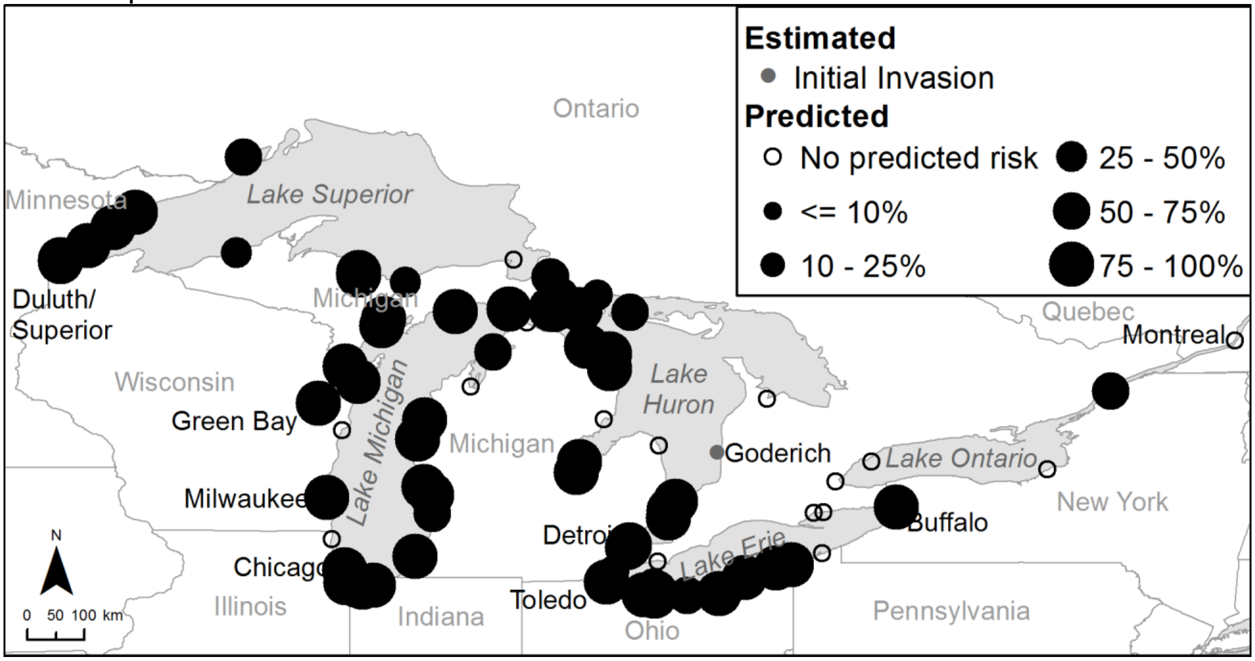

Time-step 3

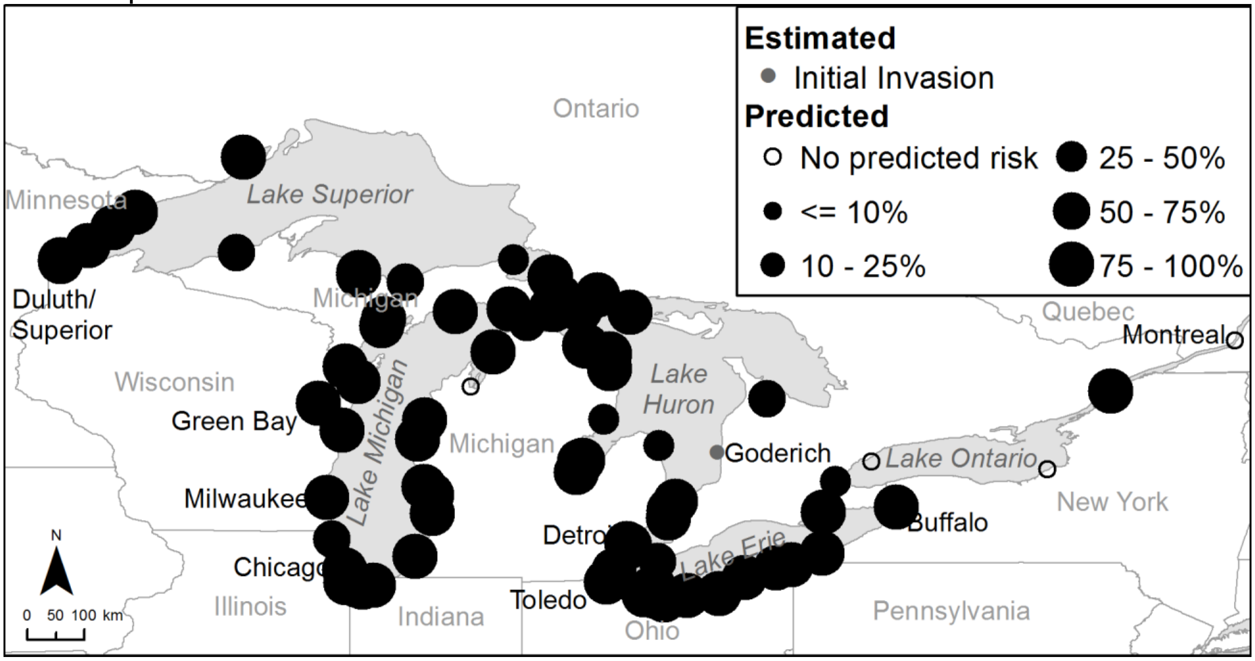

Time-step 4

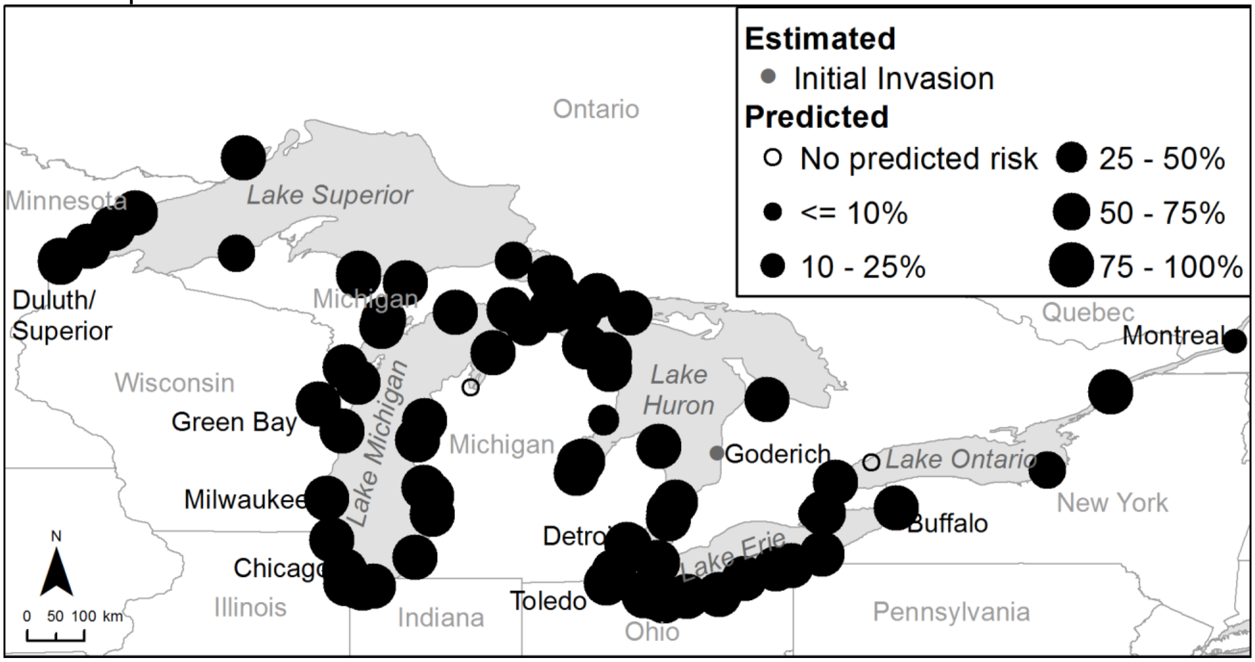

Time-step 5

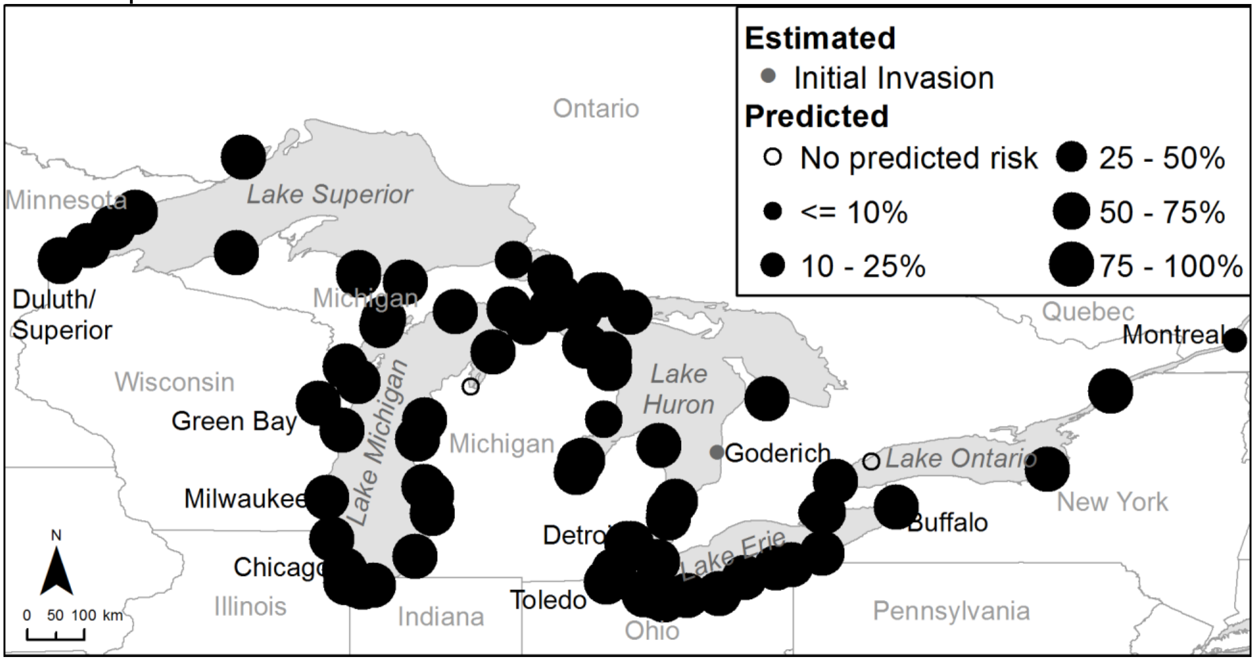

Time-step 6

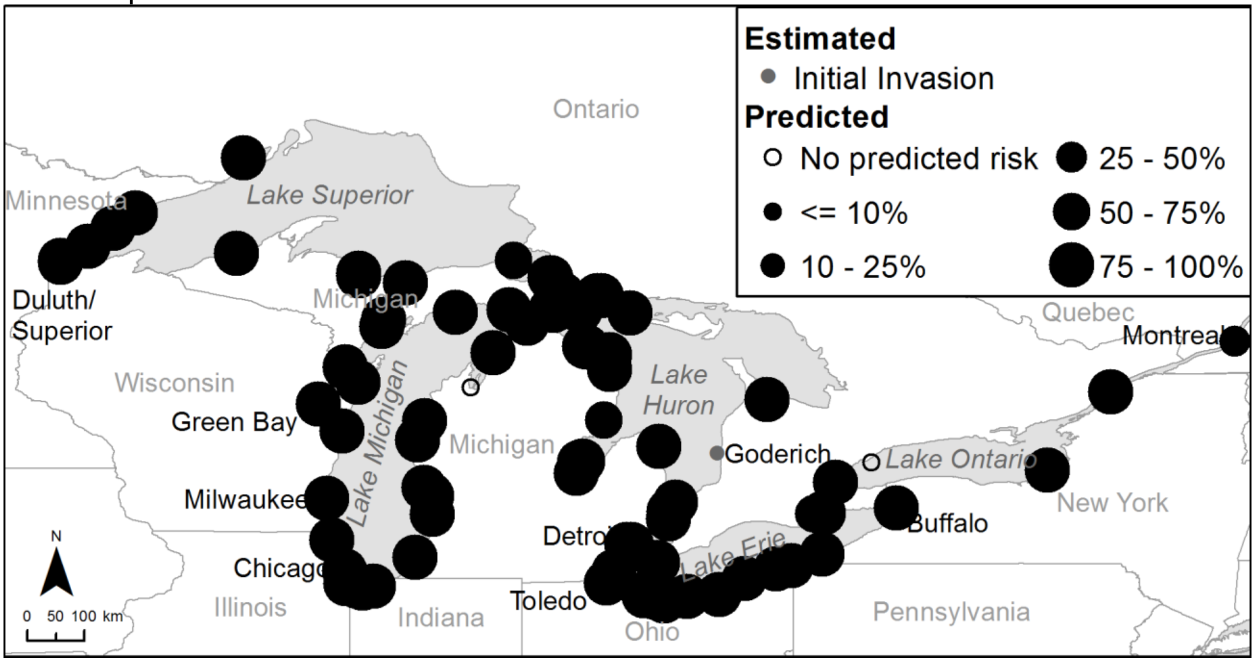

Time-step 7

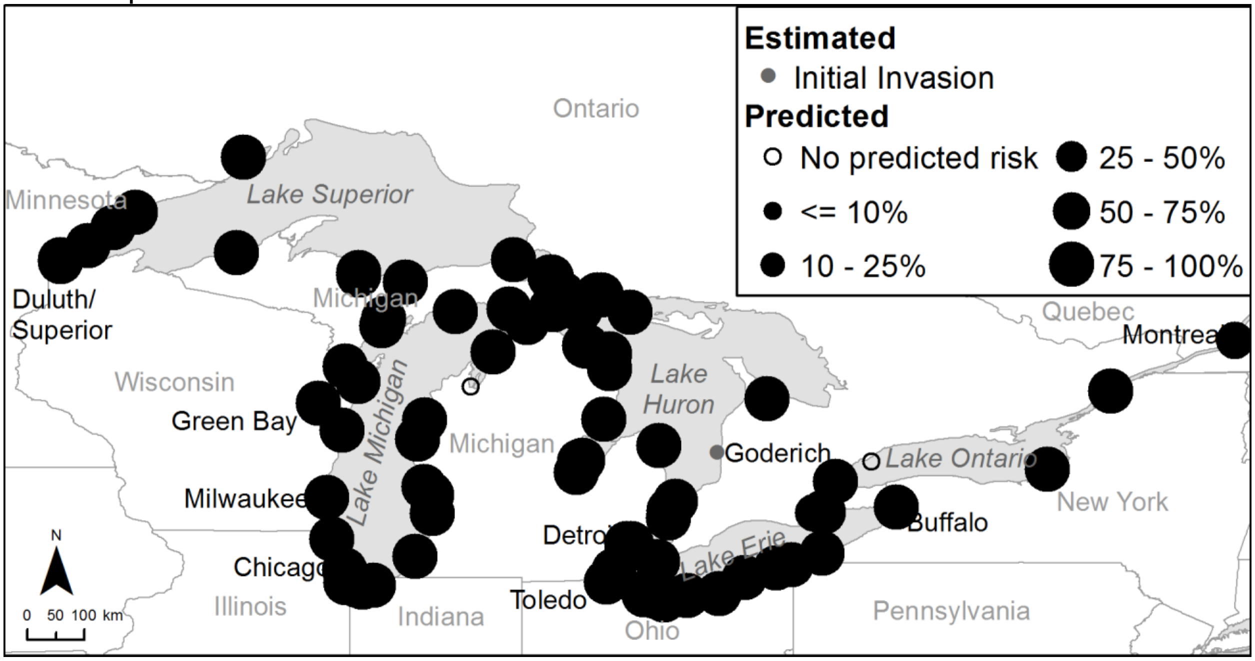

Time-step 8

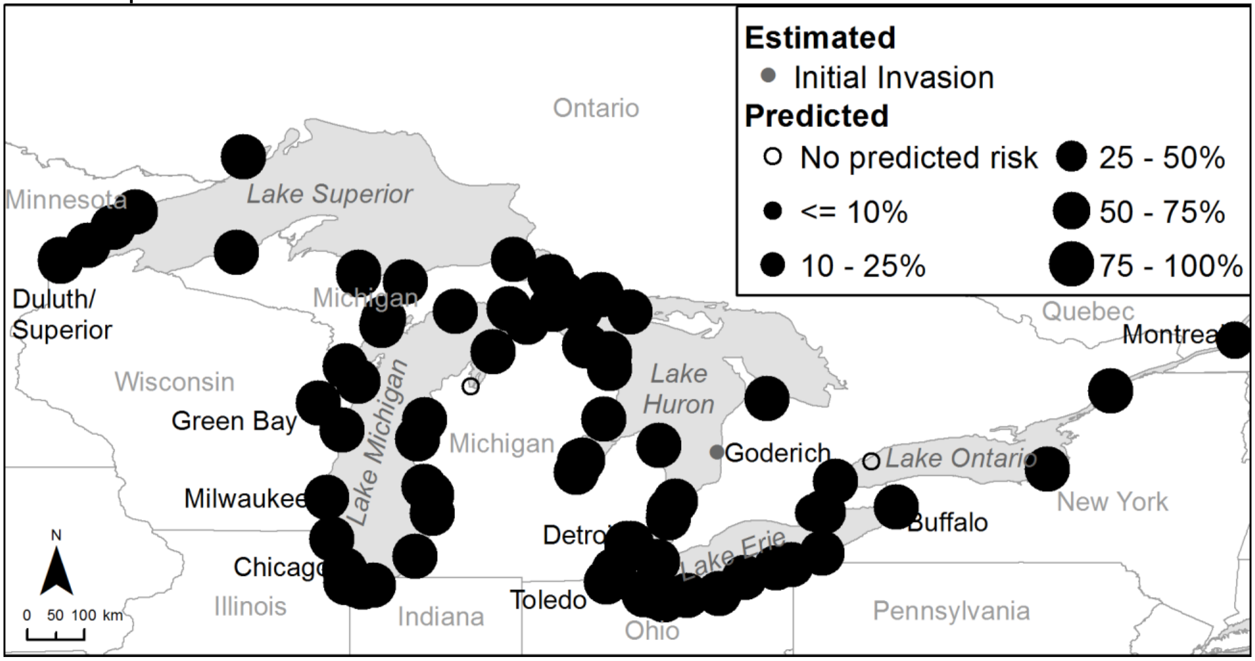

Time-step 9

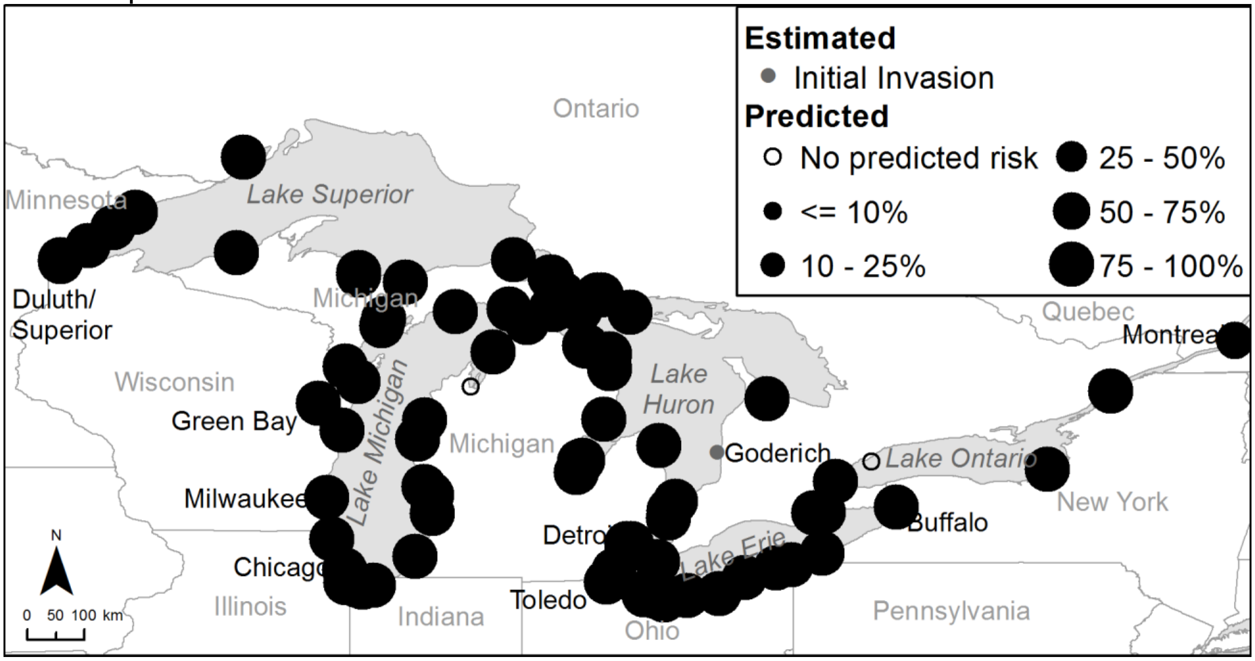

Time-step 10

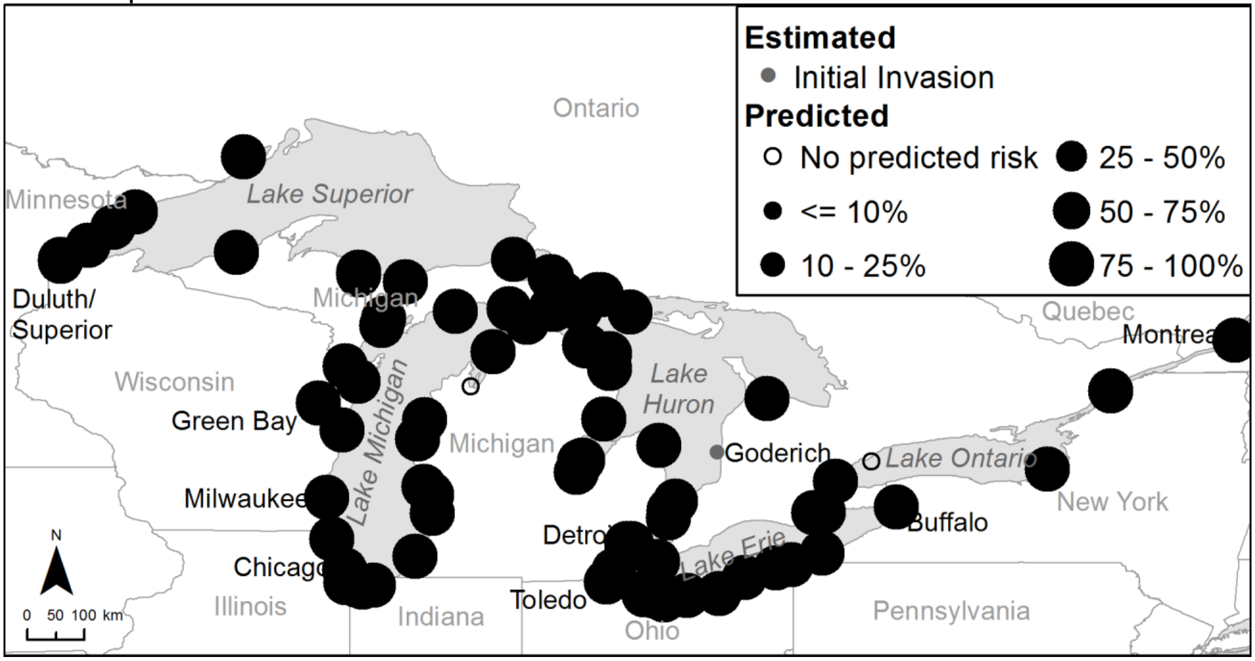

Killer Shrimp
Detroit, Michigan, USA
Dispersal Distance = 0-km and Probability of Infestation = 0.75

Time-step 1

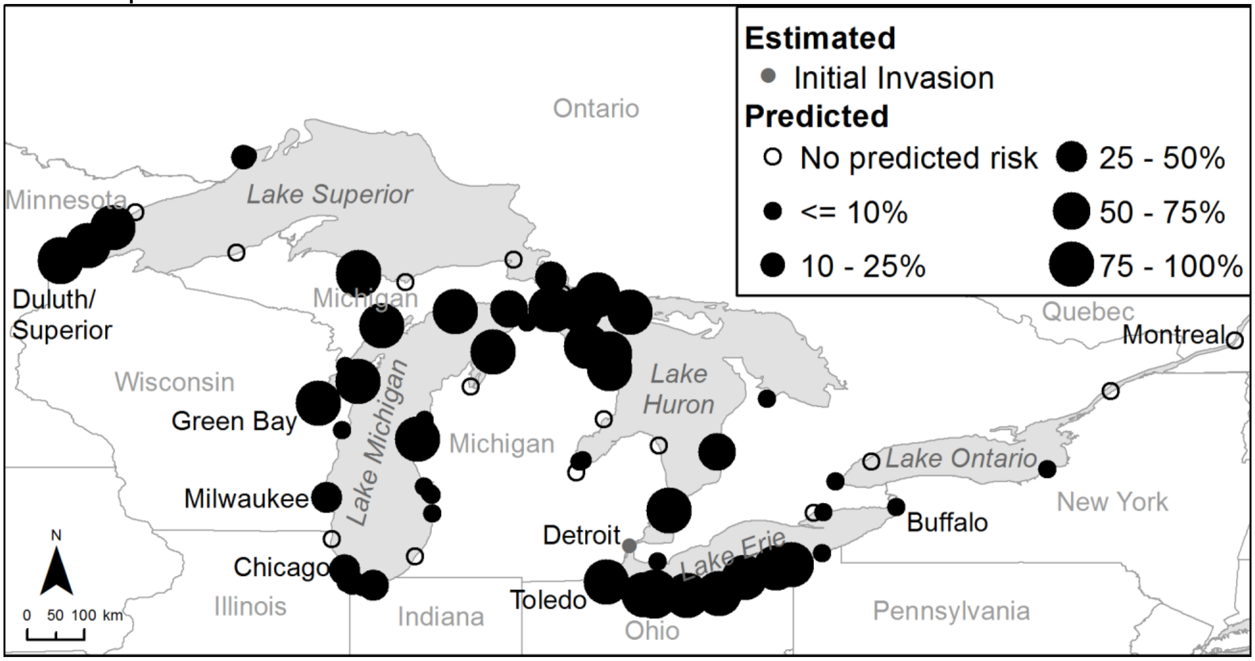

Time-step 2

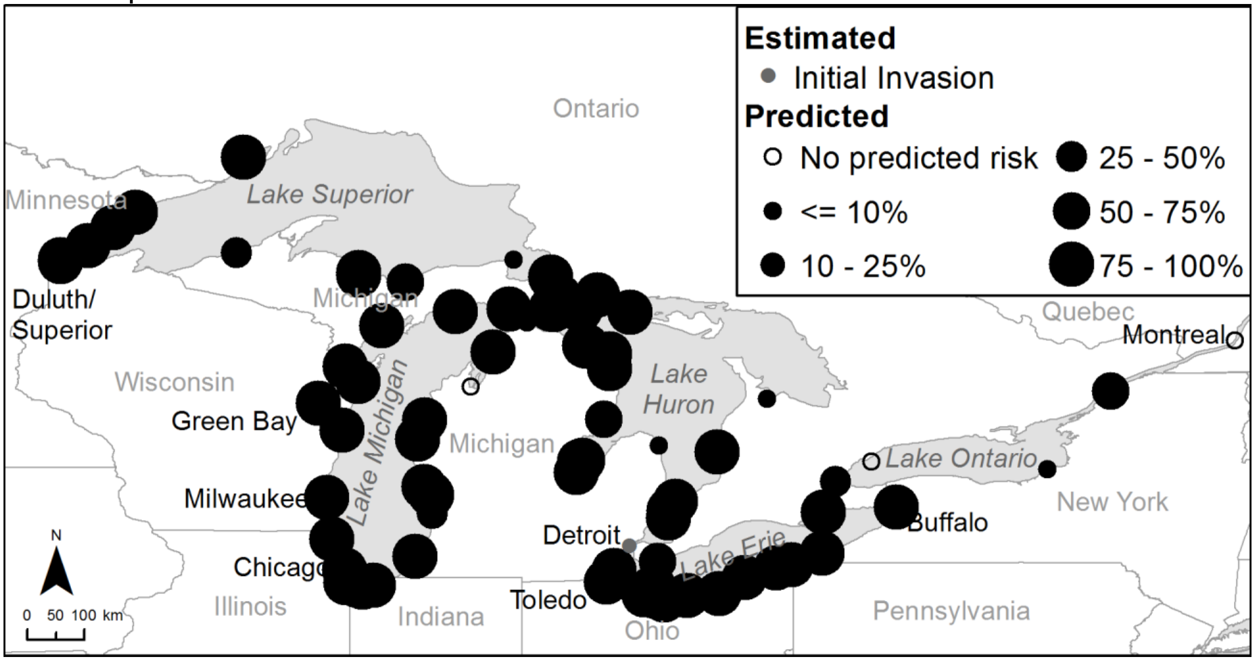

Time-step 3

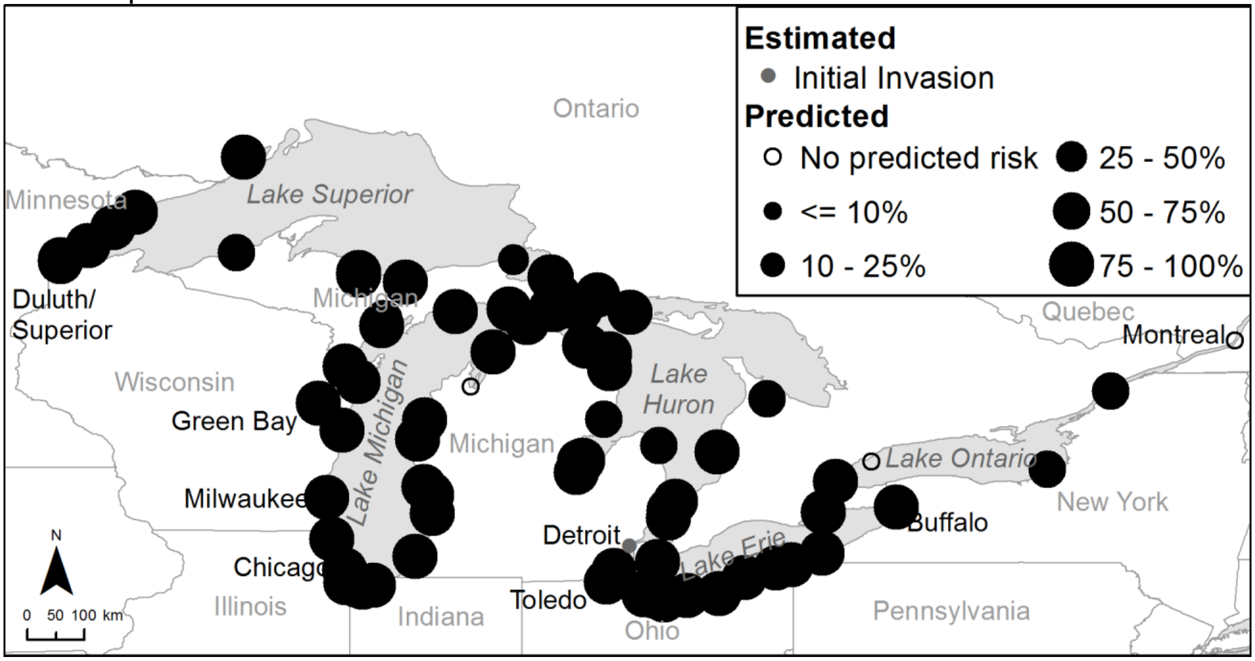

Time-step 4

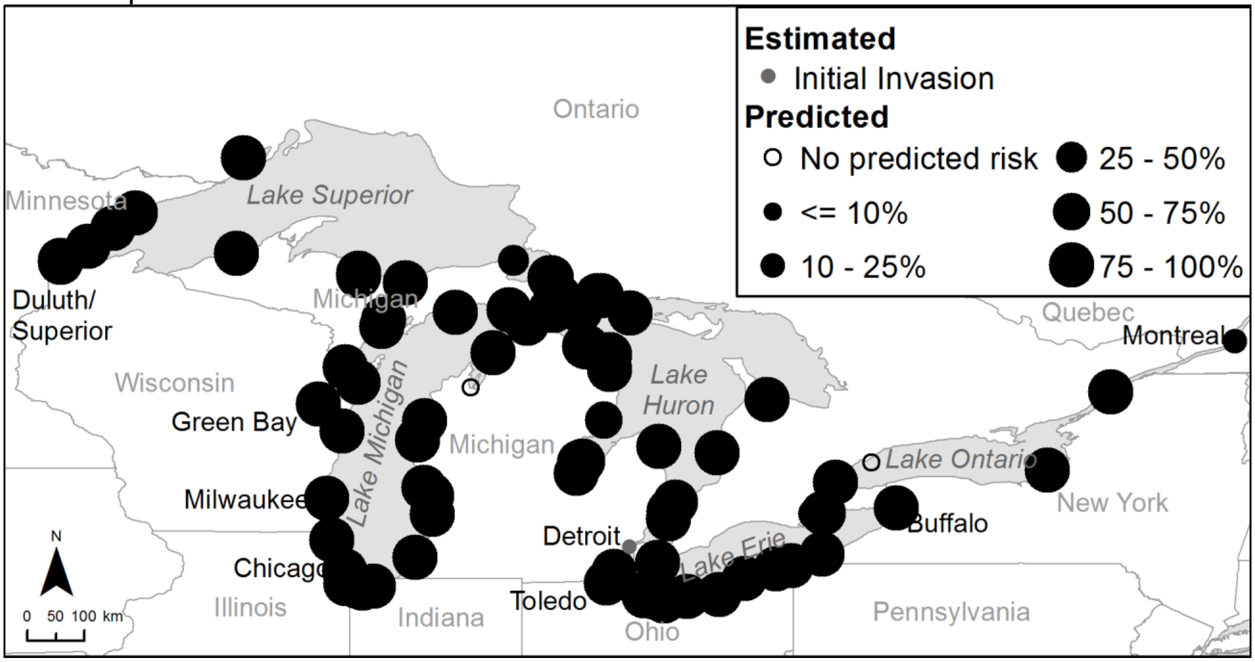

Time-step 5

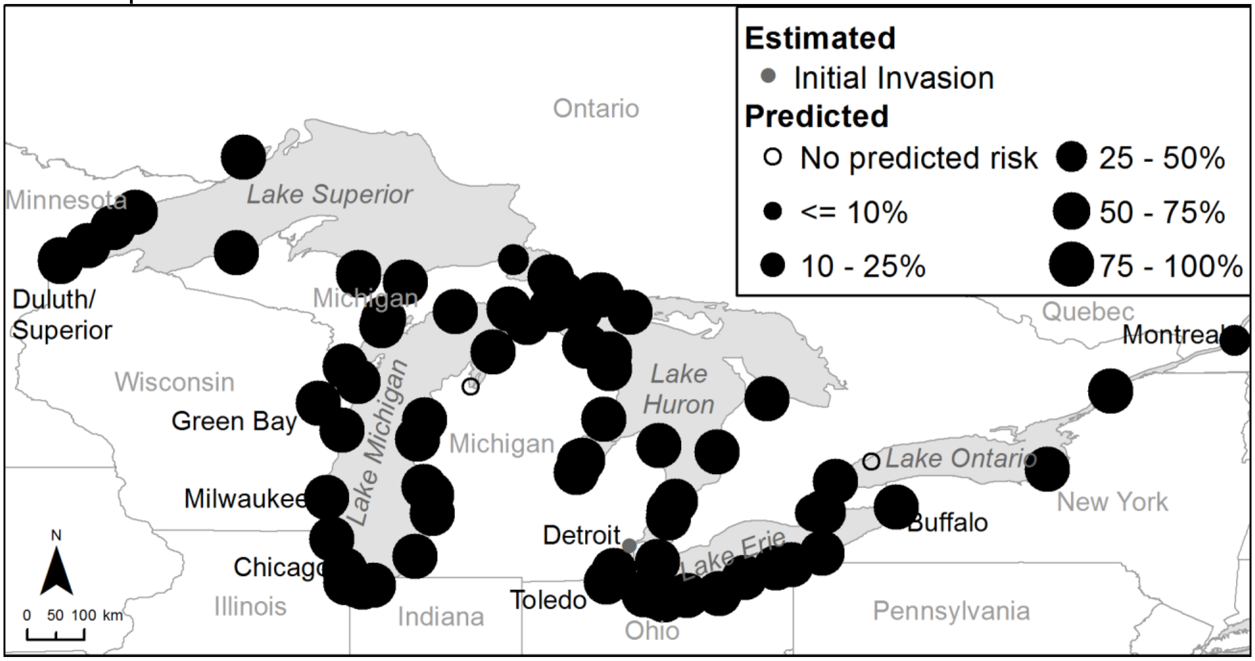

Time-step 6

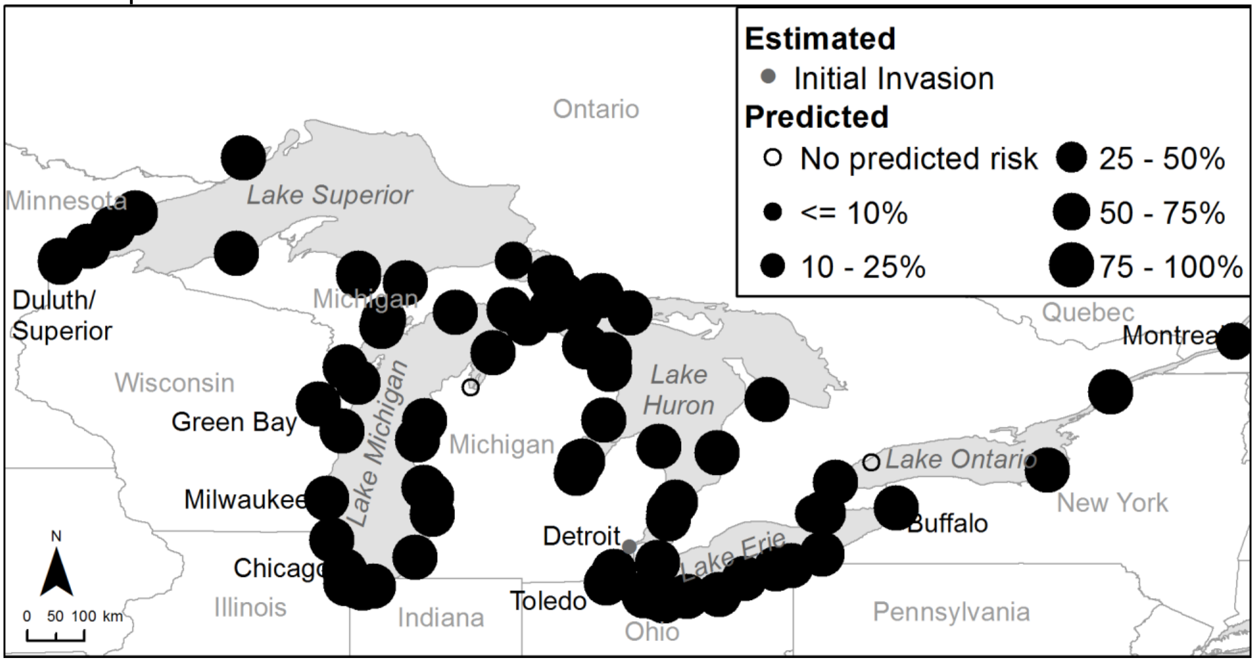

Time-step 7

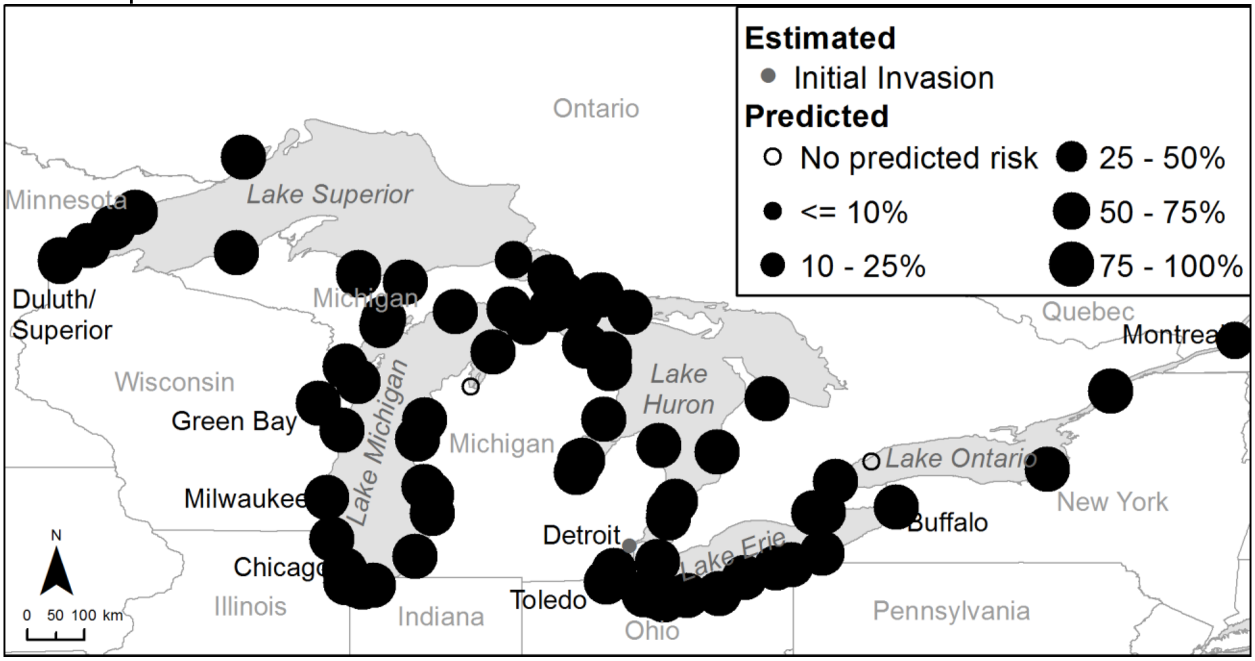

Time-step 8

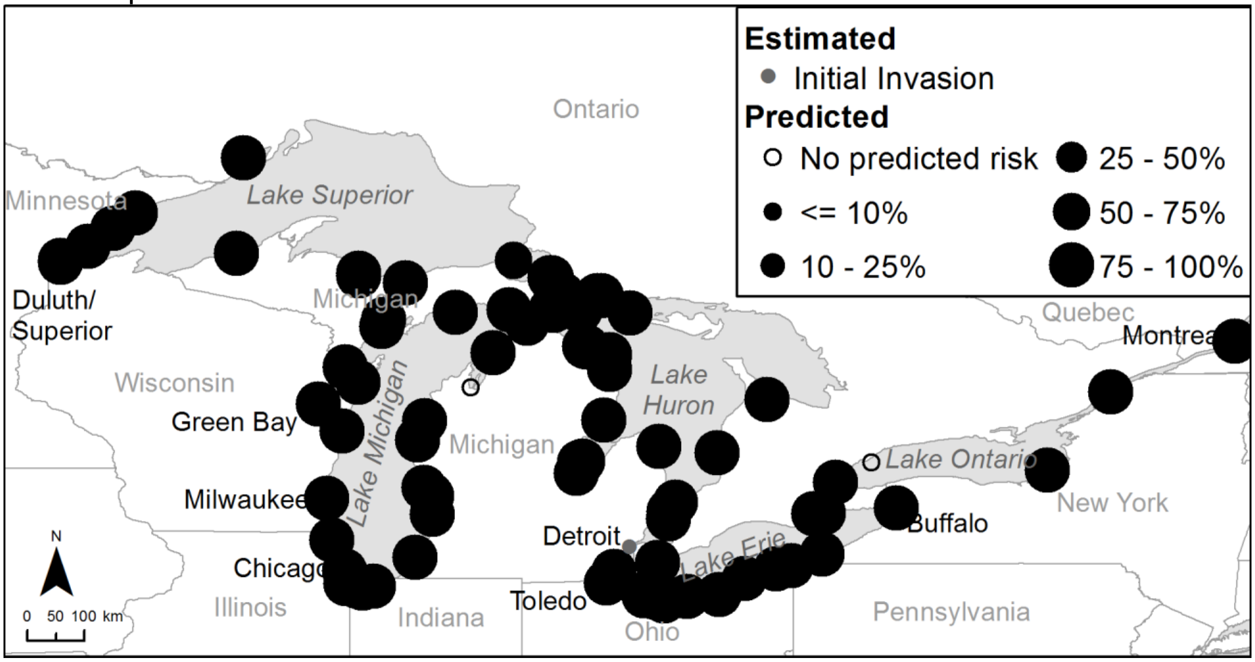

Time-step 9

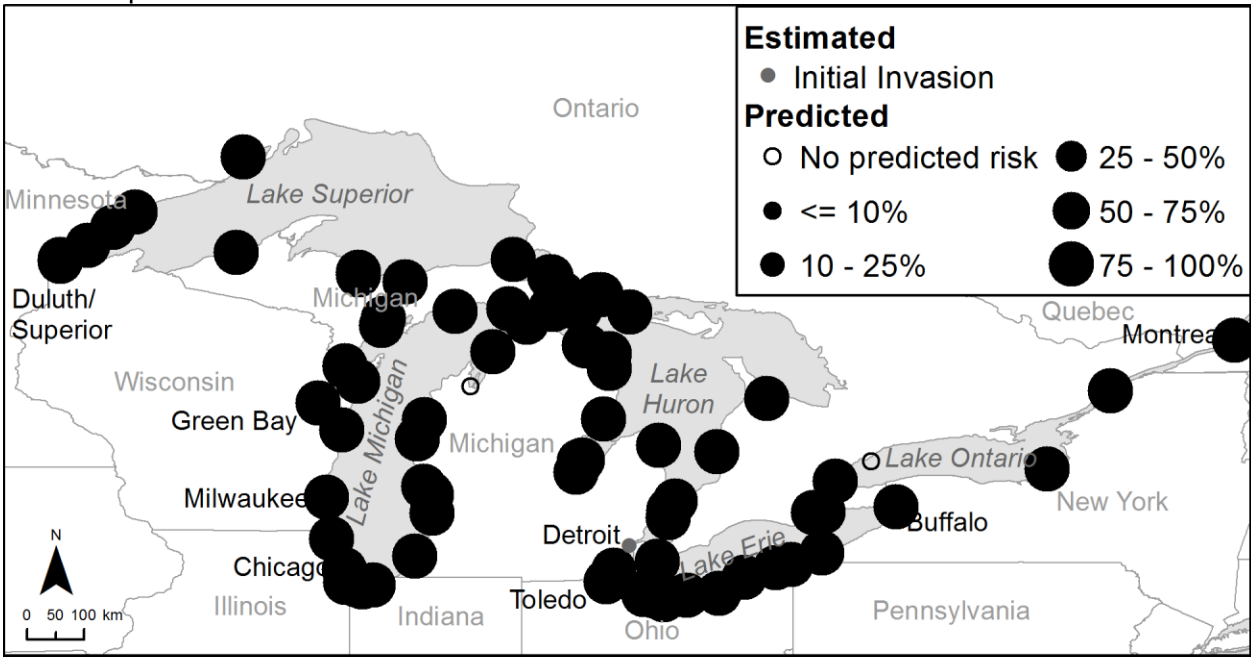

Time-step 10

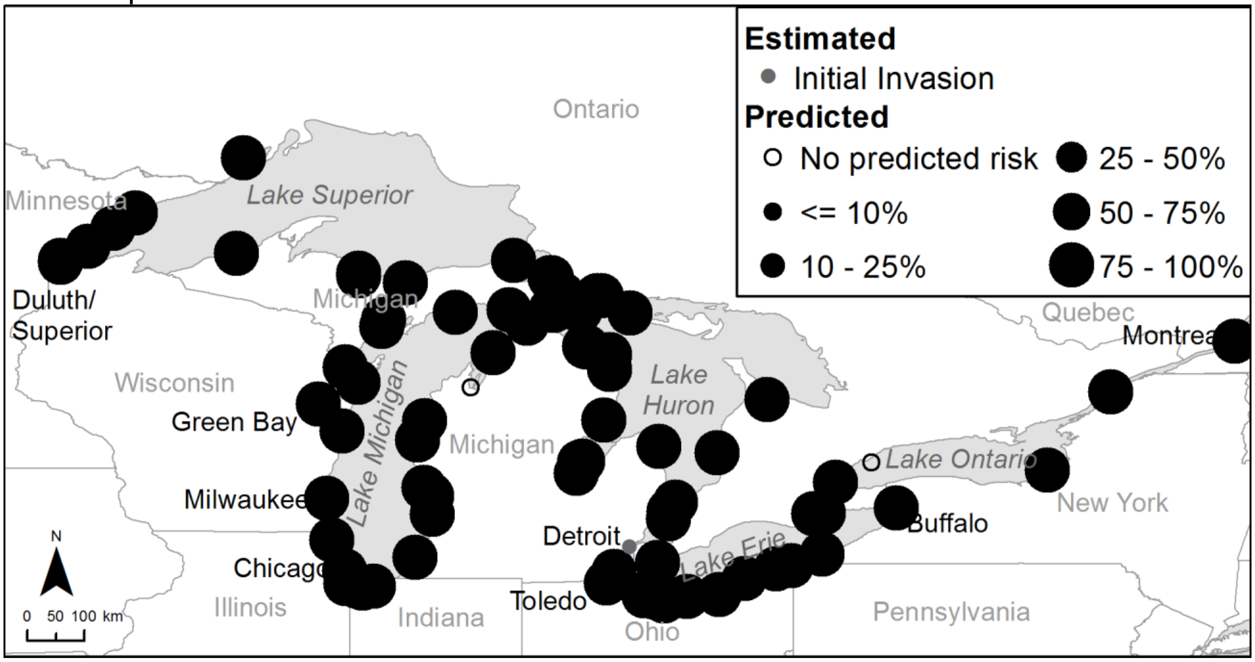

Golden Mussel
Bay City, Michigan, USA
Dispersal Distance = 20-km and Probability of Infestation = 0.75

Time-step 1

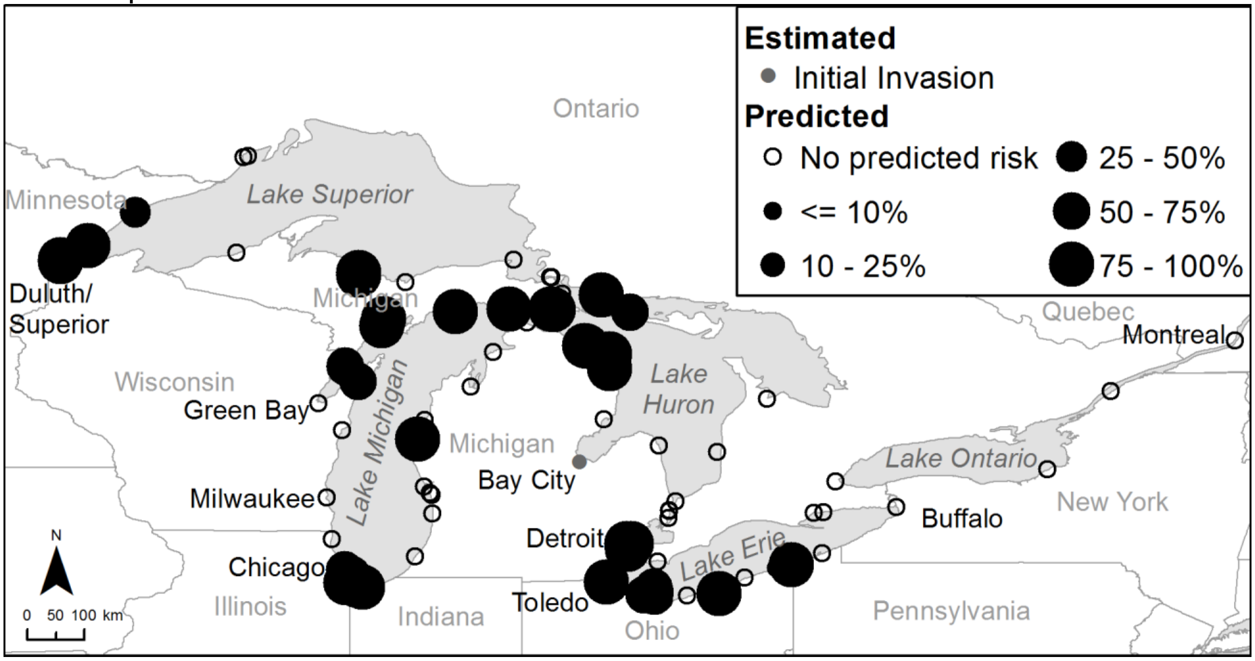

Time-step 2

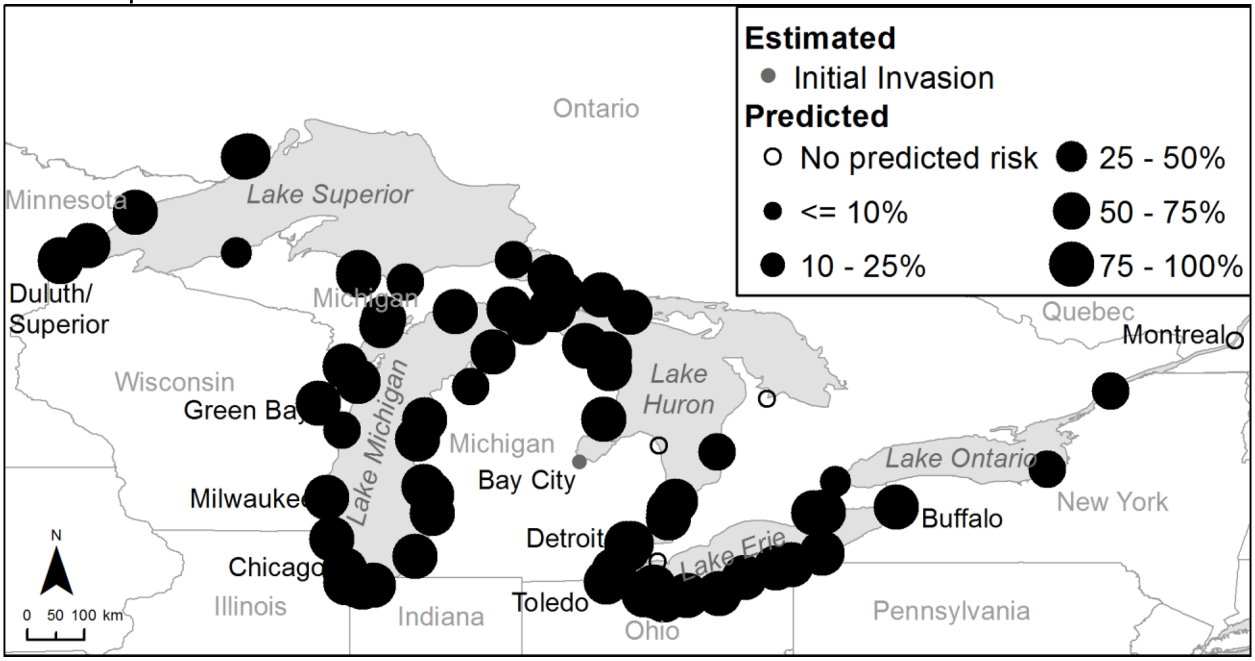

Time-step 3

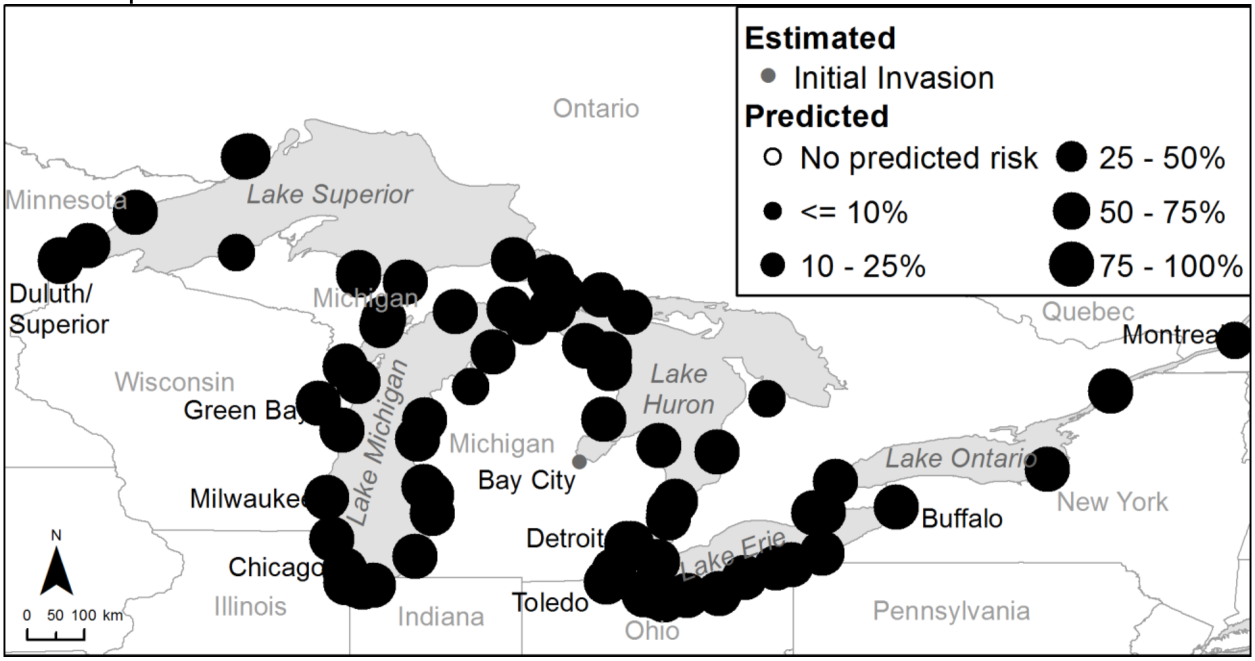

Time-step 4

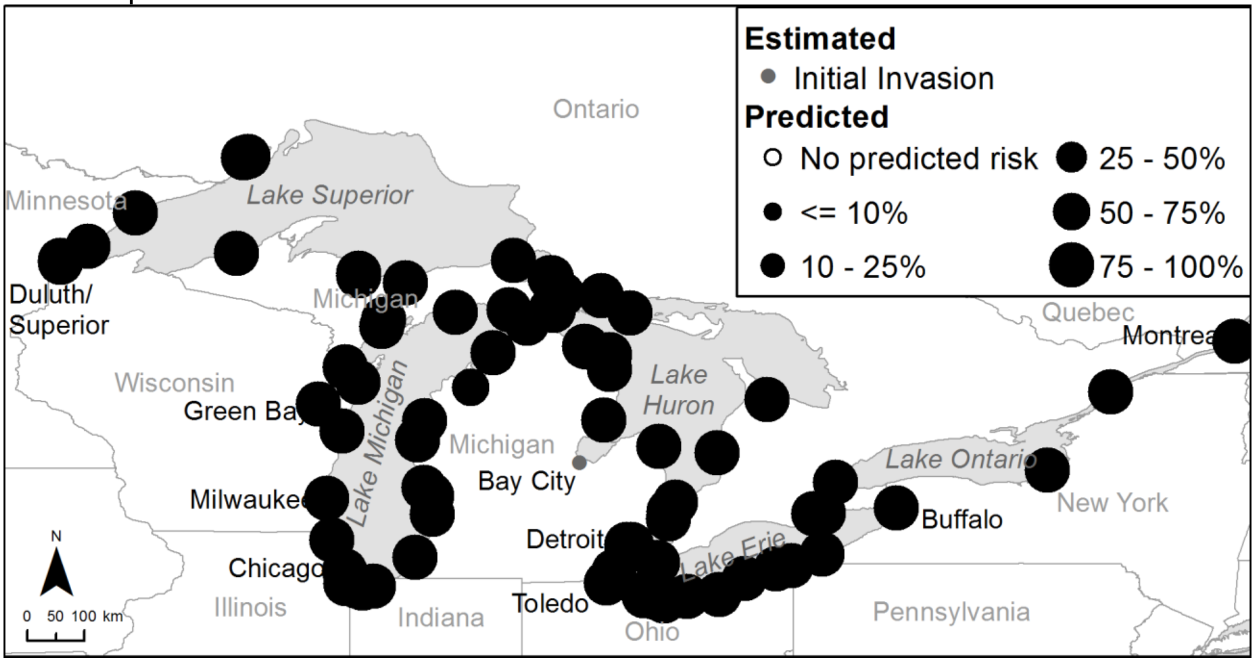

Time-step 5

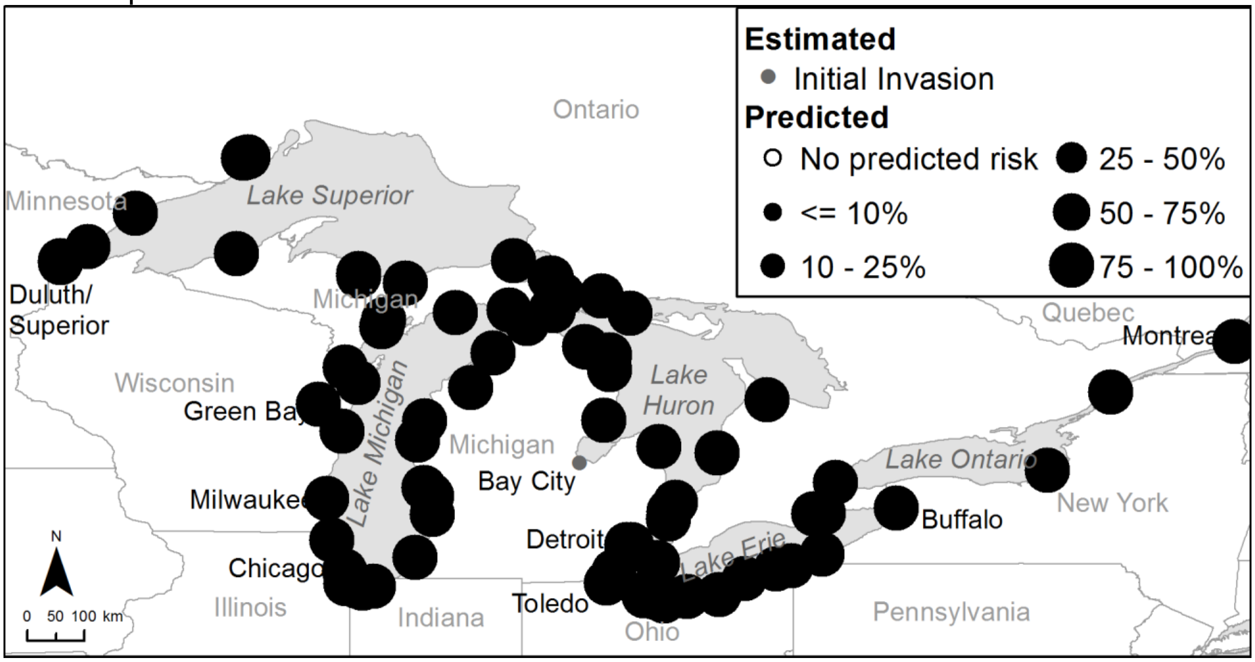

Time-step 6

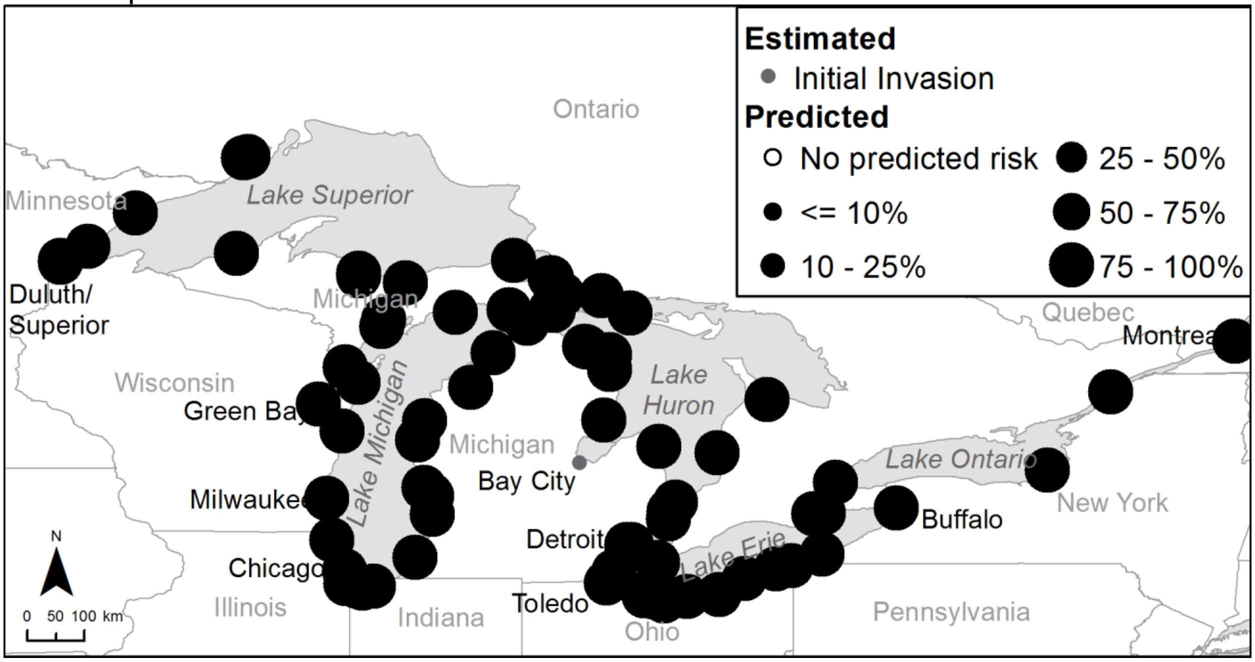

Time-step 7

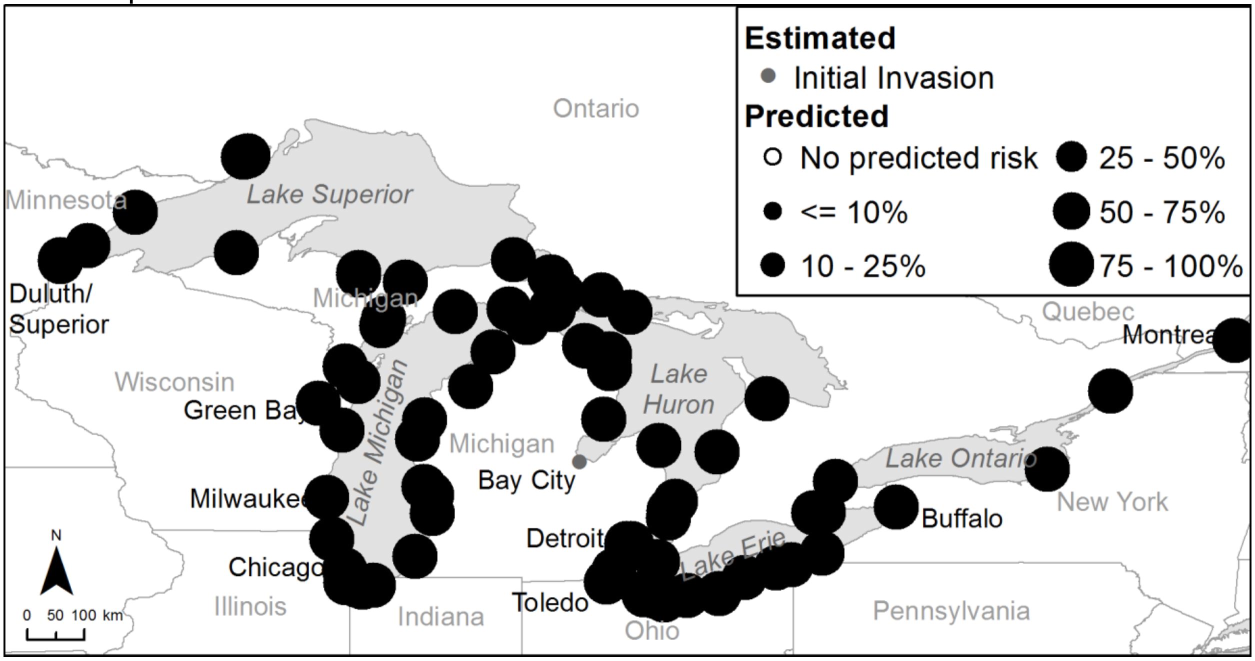

Time-step 8

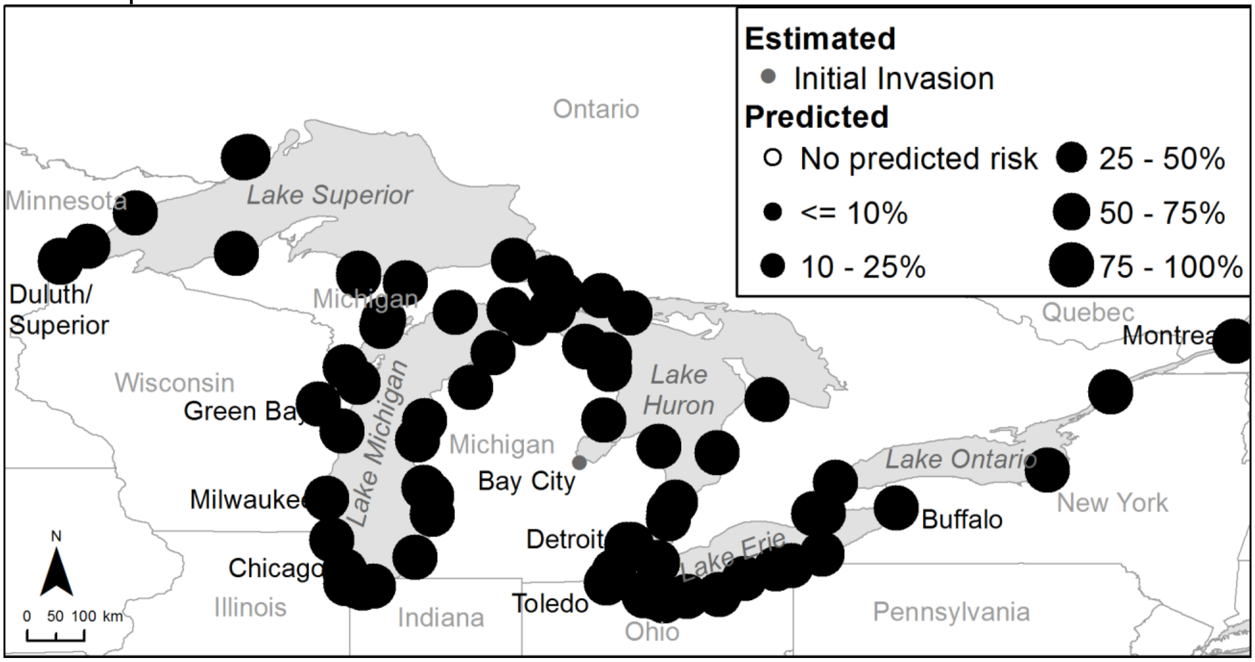

Time-step 9

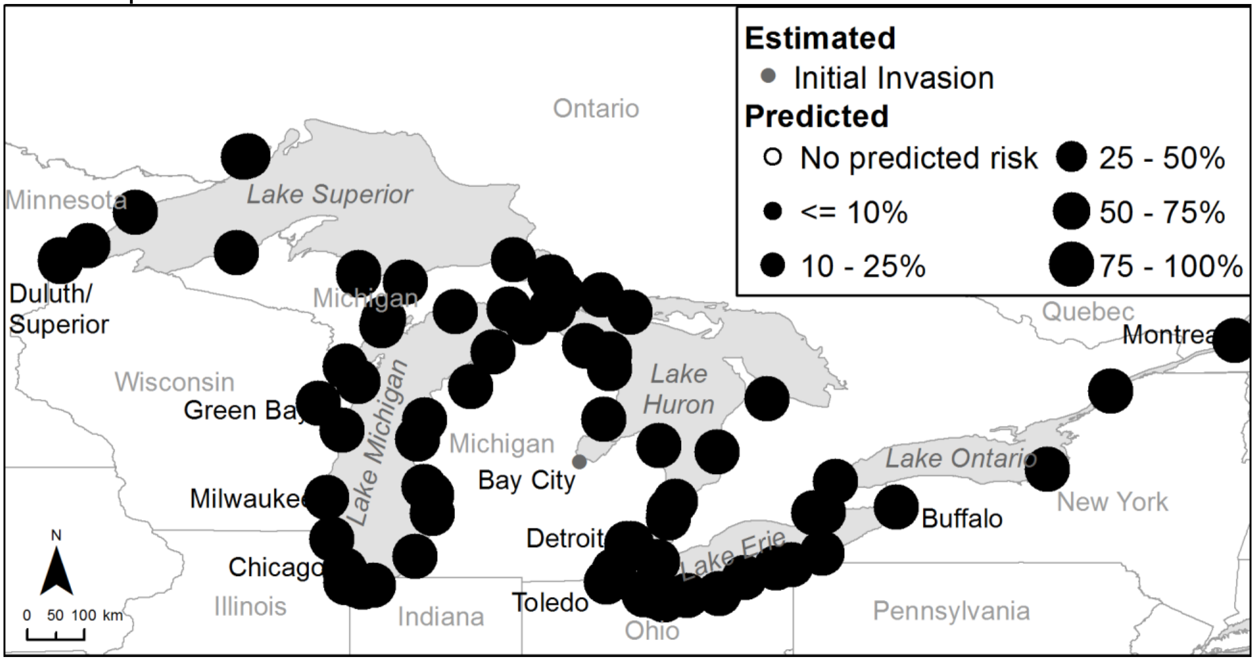

Time-step 10

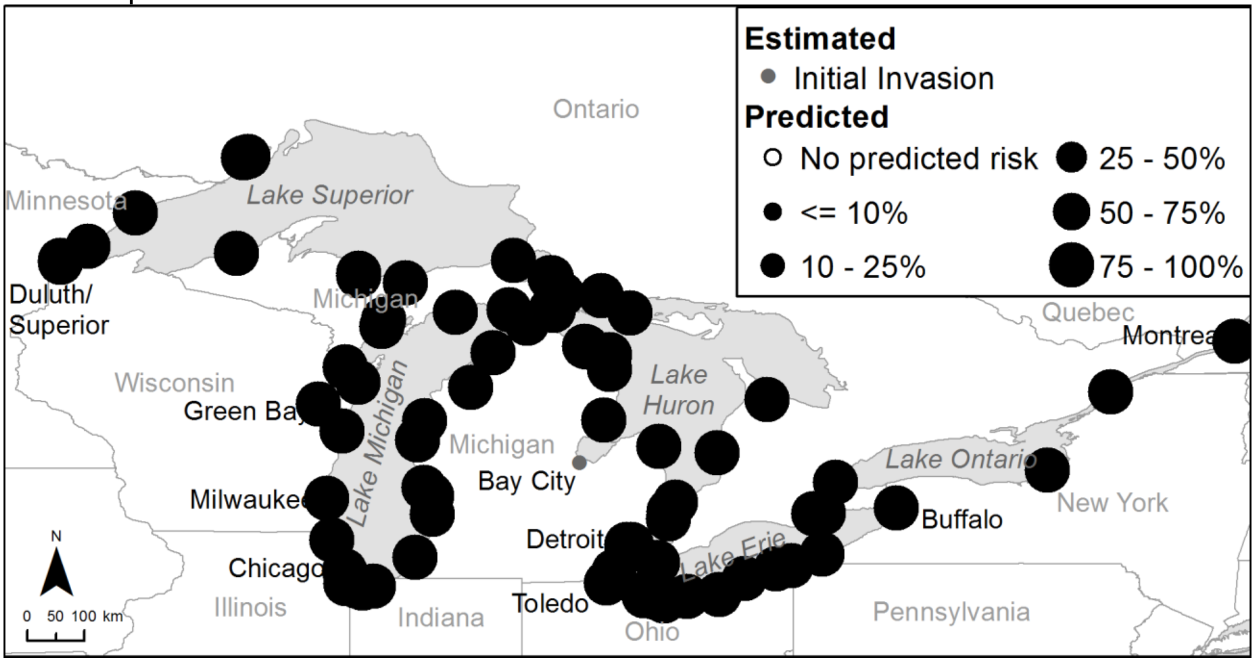

Golden Mussel
Duluth, Minnesota, USA
Dispersal Distance = 20-km and Probability of Infestation = 0.75

Time-step 1

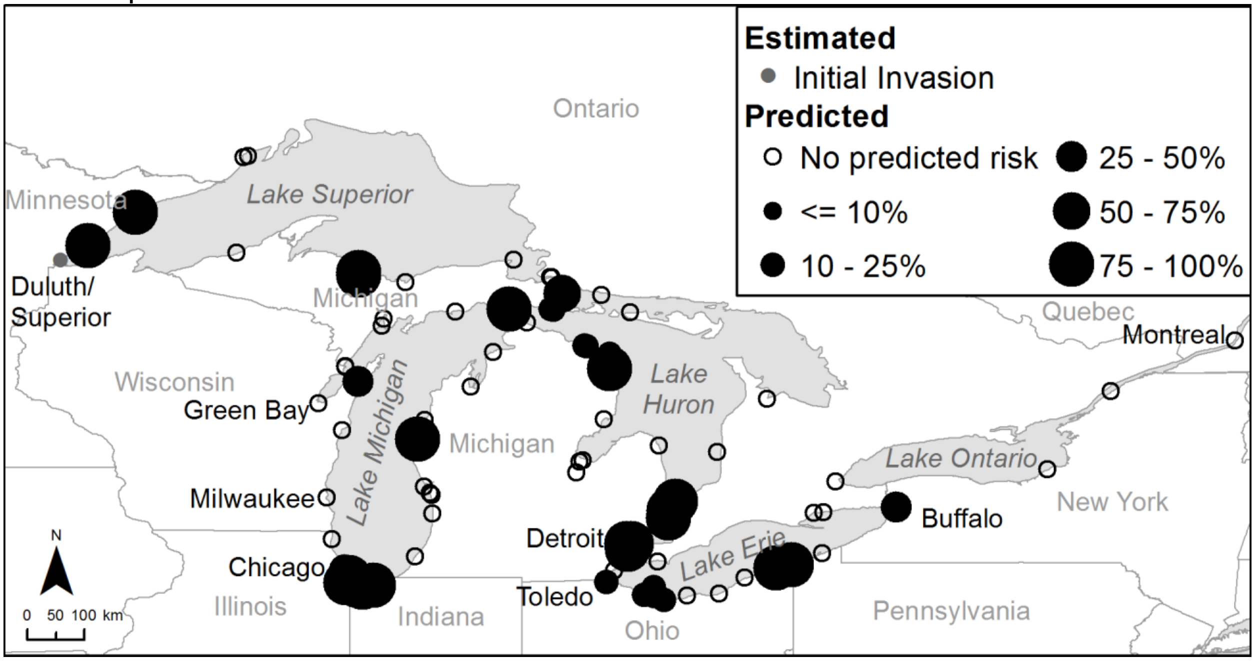

Time-step 2

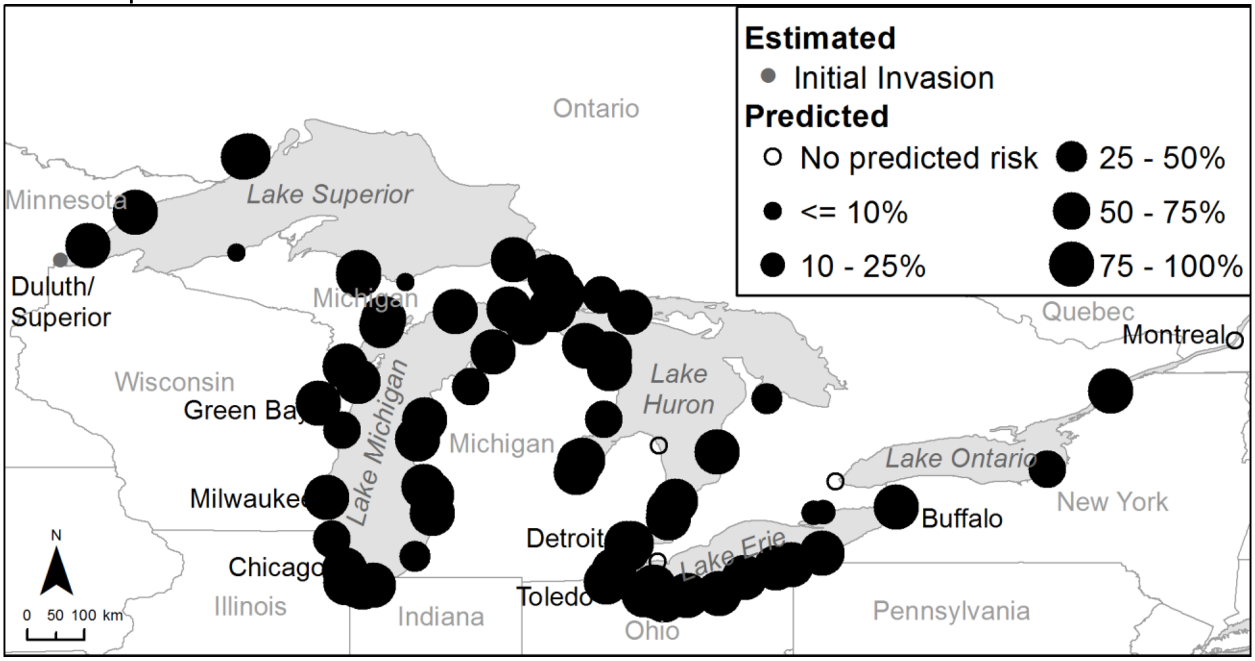

Time-step 3

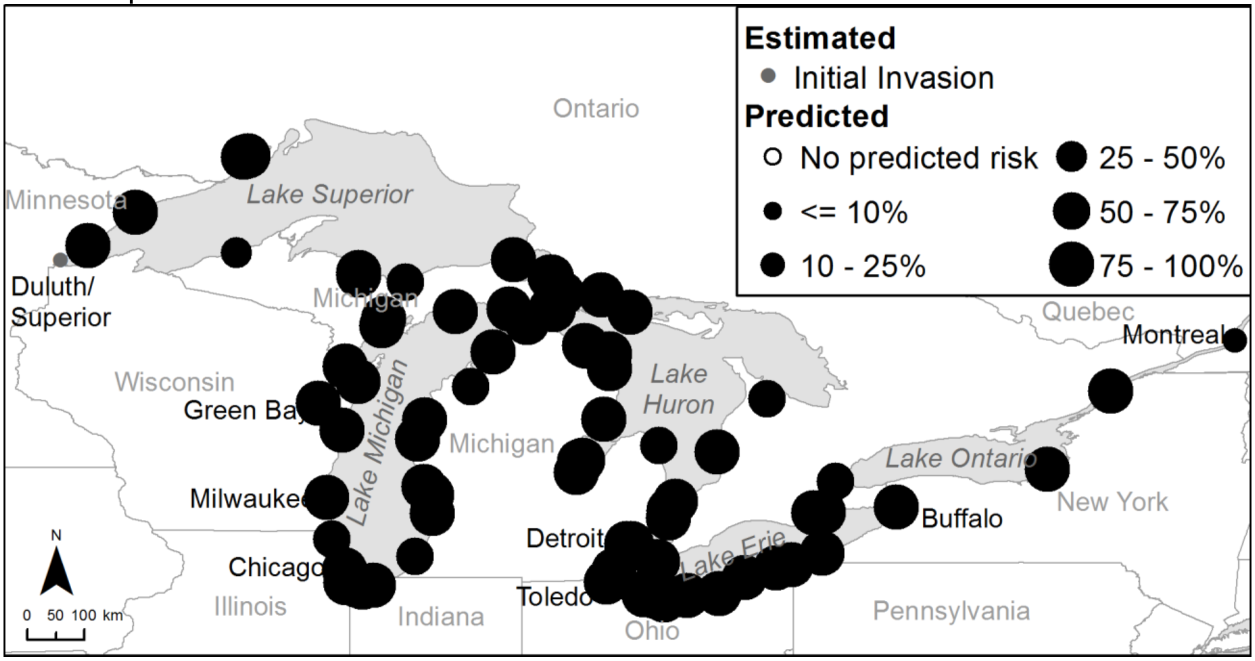

Time-step 4

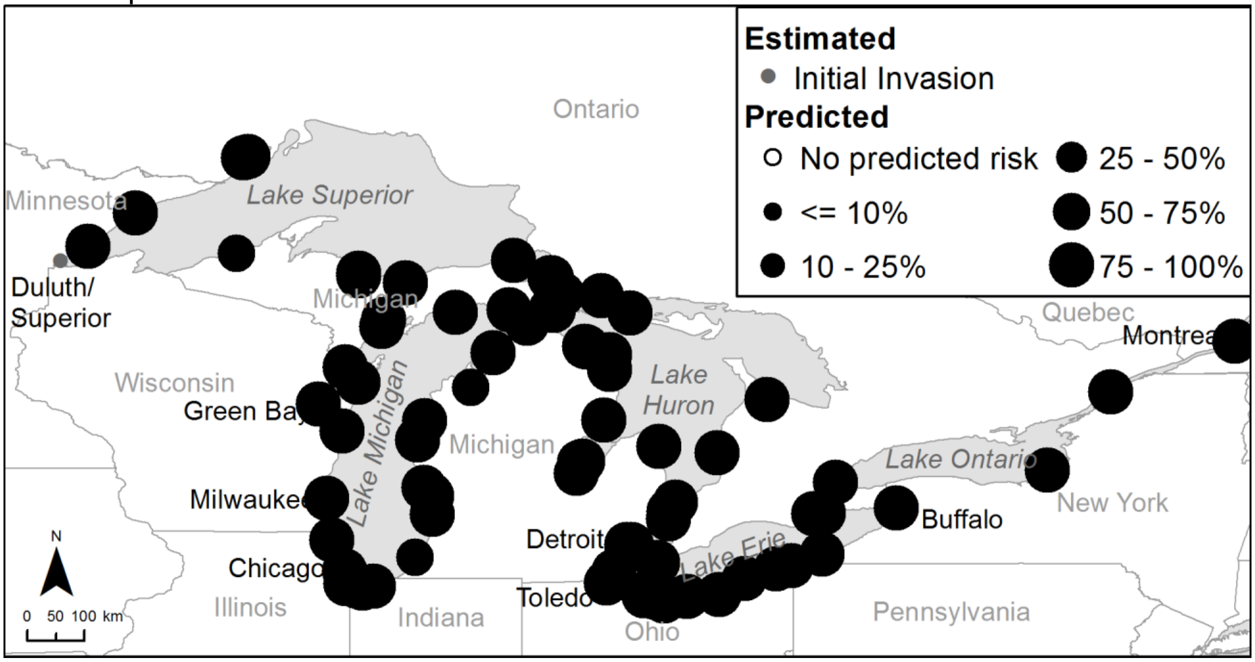

Time-step 5

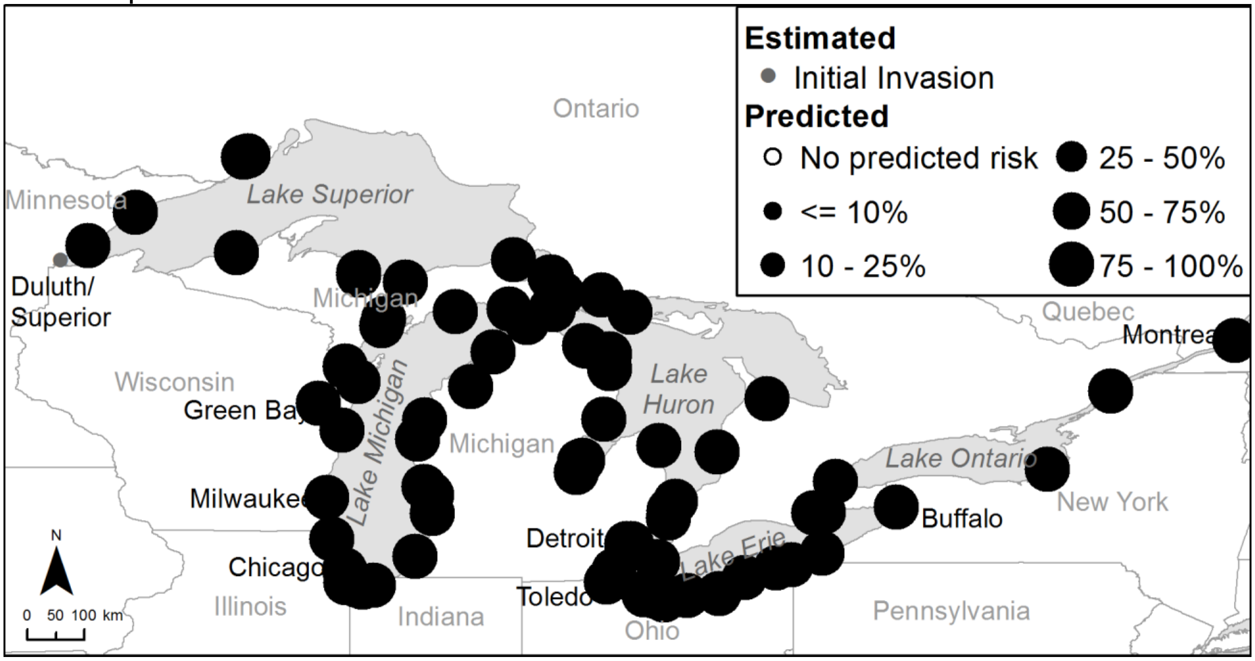

Time-step 6

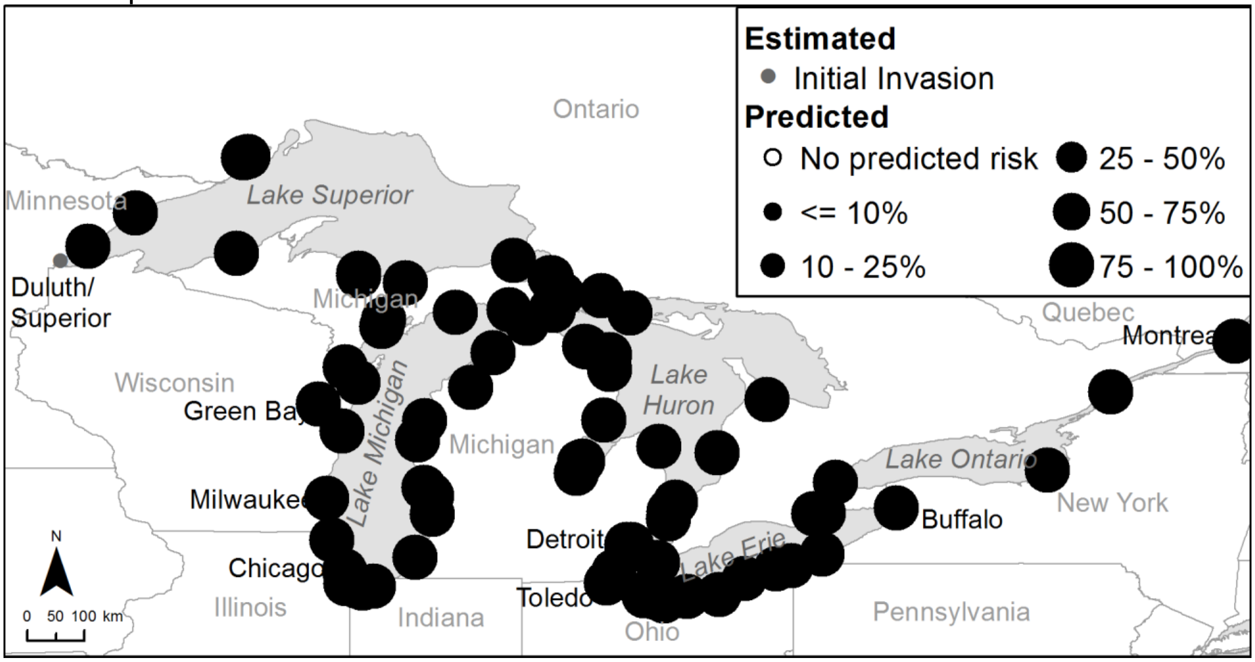

Time-step 7

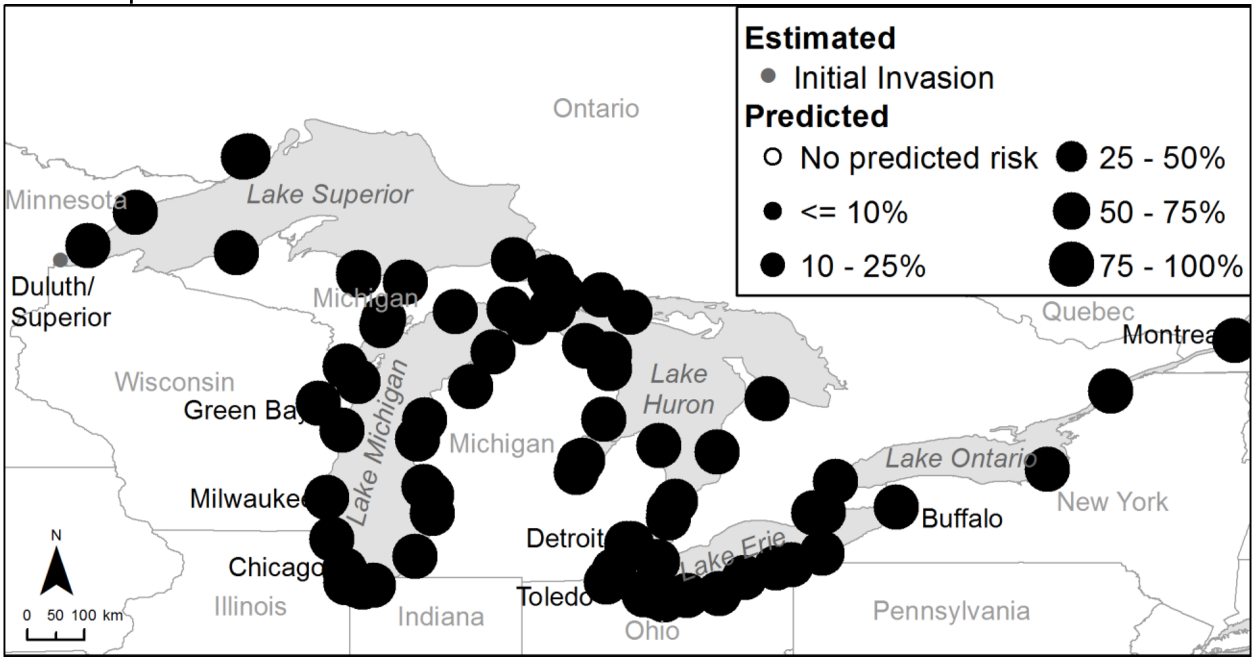

Time-step 8

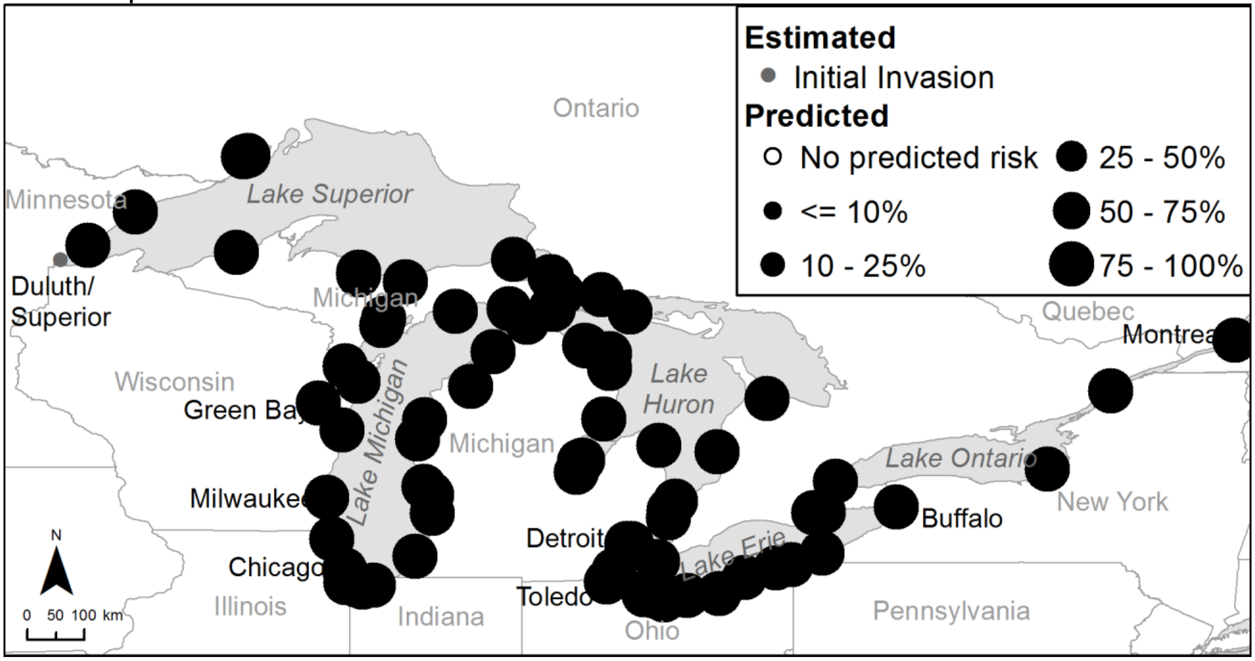

Time-step 9

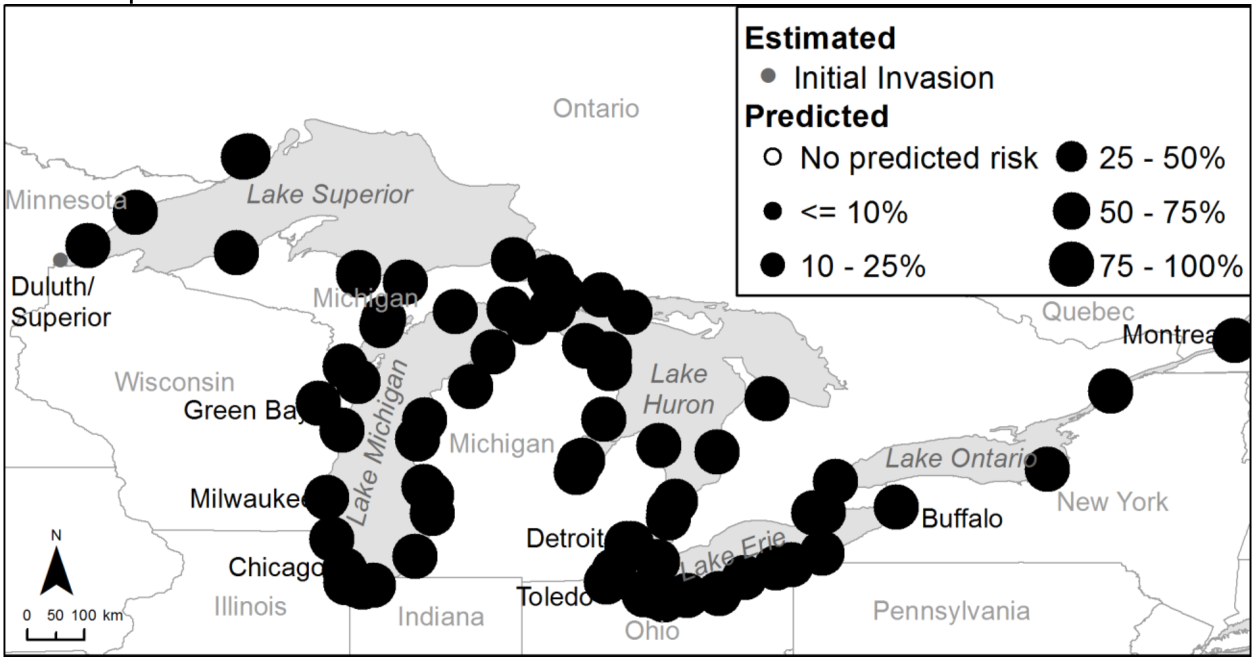

Time-step 10

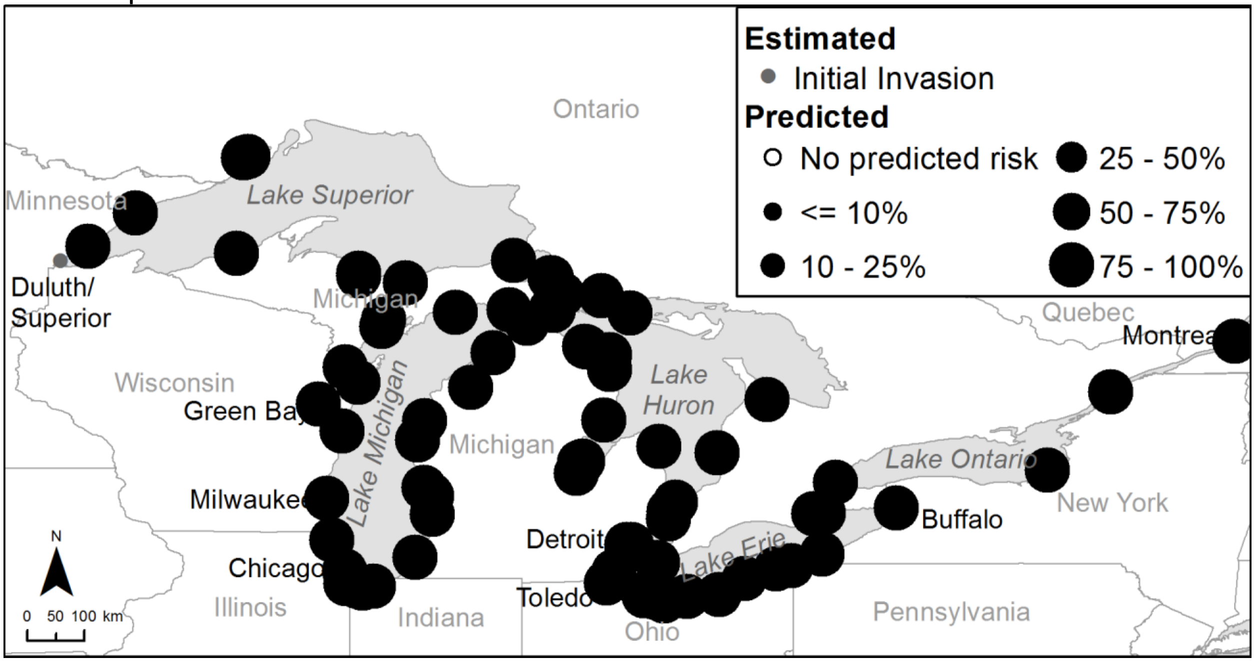

Supplement: Information S2 — Prediction Maps. Included are the resulting predictions modeled for Eurasian Ruffe, killer shrimp, and golden mussel. Ten time-steps were modeled from each of the invasion start locations for each species. Results are also included for both sets of parameter values used to predict the future spread of Eurasian Ruffe. Killer shrimp spread predictions were not modeled from Superior, Wisconsin, USA due to its proximity to Duluth, Minnesota, USA. (PDF) [file pone.0114217.s002.pdf]
